# Structure-guided design of an Hsp90β N-terminal isoform-selective inhibitor

**DOI:** 10.1038/s41467-017-02013-1

**Published:** 2018-01-30

**Authors:** Anuj Khandelwal, Caitlin N. Kent, Maurie Balch, Shuxia Peng, Sanket J. Mishra, Junpeng Deng, Victor W. Day, Weiya Liu, Chitra Subramanian, Mark Cohen, Jeffery M. Holzbeierlein, Robert Matts, Brian S. J. Blagg

**Affiliations:** 1 Department of Medicinal Chemistry, The University of Kansas, 1251 Wescoe Hall Drive, Malott Hall 4048, Lawrence, KS 66045 USA; 2 Department of Chemistry and Biochemistry, The University of Notre Dame, 305 McCourtney Hall, Notre Dame, IN 46556 USA; 3 Department of Biochemistry and Molecular Biology, 246C Noble Research Center, Oklahoma State University, Stillwater, OK 74078 USA; 4 Department of Chemistry, The University of Kansas, 1251 Wescoe Hall Drive, Malott Hall 2010, Lawrence, KS 66045 USA; 5 Department of Urologic Oncology, University of Kansas Medical Center, 3901 Rainbow Boulevard, Kansas City, KS 66160 USA; 6 Department of Pharmacology, University of Michigan School of Medicine, 1150 W. Medical Center Dr., Ann Arbor, MI 48109 USA

## Abstract

The 90 kDa heat shock protein (Hsp90) is a molecular chaperone responsible for folding proteins that are directly associated with cancer progression. Consequently, inhibition of the Hsp90 protein folding machinery results in a combinatorial attack on numerous oncogenic pathways. Seventeen small-molecule inhibitors of Hsp90 have entered clinical trials, all of which bind the Hsp90 N-terminus and exhibit pan-inhibitory activity against all four Hsp90 isoforms. pan-Inhibition of Hsp90 appears to be detrimental as toxicities have been reported alongside induction of the pro-survival heat shock response. The development of Hsp90 isoform-selective inhibitors represents an alternative approach towards the treatment of cancer that may limit some of the detriments. Described herein is a structure-based approach to design isoform-selective inhibitors of Hsp90β, which induces the degradation of select Hsp90 clients without concomitant induction of Hsp90 levels. Together, these initial studies support the development of Hsp90β-selective inhibitors as a method to overcome the detriments associated with pan-inhibition.

## Abstract (web summary)

The molecular chaperone Hsp90 oversees the folding of many proteins associated with cancer progression but existing small-molecule inhibitors of this pathway are not isoform-selective. Here, the authors rationally design an Hsp90 inhibitor that displays high selectivity for the Hsp90β isoform.

## Introduction

The Hsp90 family of proteins consist of four isoforms. Hsp90β is constitutively expressed in the cytoplasm, Hsp90α is expressed in the cytosol in response to cellular stress, Grp94 resides in the endoplasmic reticulum, and Trap-1 is localized to the mitochondria^1–3^. Together, these molecular chaperones are responsible for the conformational maturation, activation, and/or trafficking of ~300 Hsp90-dependent substrates^4–9^. Many of the proteins dependent upon Hsp90 are essential to the growth and proliferation of cancer cells. In fact, proteins associated with all 10 hallmarks of cancer are dependent upon the Hsp90 protein folding machinery^10^. Consequently, Hsp90 has emerged as a promising target for the development of anti-cancer chemotherapeutics^11–13^.

Seventeen small molecule inhibitors of Hsp90 have entered clinical trials, all of which exhibit pan Hsp90 inhibitory activity against all four isoforms^14–17^. Many of the compounds have produced cardiotoxicity, gastrointestinal toxicity, and/or ocular toxicity amongst other side effects^18–22^. Recent studies have determined that maturation of the hERG channel is also Hsp90 dependent, and specifically depends upon the Hsp90α isoform^23^. In addition, pan Hsp90 inhibition induces the pro-survival heat shock response, which leads to induction of Hsp27, Hsp40, Hsp70, and Hsp90, requiring the escalation of doses to overcome increased Hsp90 expression^24–26^. Among all four isoforms, specific roles for Grp94 and the consequences of selective Grp94-inhibition have been deconvoluted. Selective Grp94 inhibition has emerged as a promising approach for the treatment of glaucoma, multiple myeloma and metastasis. Recently, Patel and co-workers showed that Grp94 inhibition represents a non-toxic approach to treat Her2 positive cancers. Collectively, these findings highlight the advantages of isoform-selective Hsp90 inhibition and warrant a better understanding played by the specific roles of individual isoforms.

Hydrolysis of ATP by the N-terminal nucleoside binding pocket is required for the maturation of client protein substrates, and all four Hsp90’s share >70% identity in this region and 21 out of the 29 residues are totally conserved and the remaining 8 share high similarity^27–29^. Consequently, the discovery of isoform-selective inhibitors has been challenging^30,31^. Since Grp94 exhibits the lowest similarity with other Hsp90 isoforms, three scaffolds manifesting Grp94-selective inhibition were recently reported^32–34^. However, Hsp90α and Hsp90β share ~95% identity in this binding site and only two amino acids differ between these isoforms, making the development of Hsp90α- or Hsp90β-selective inhibitors most challenging. Based on differences exhibited between these two amino acids in the Hsp90α and Hsp90β crystal structures, perturbation of the conserved water molecules that mediate interactions with inhibitory ligands were carefully analyzed, and a scaffold was developed that selectively inhibits the Hsp90β isoform with > 50-fold selectivity. The design and development of an Hsp90β-selective N-terminal scaffold is reported herein.

## Results

### A water-mediated network of hydrogen bonds

**Fig. 1:**
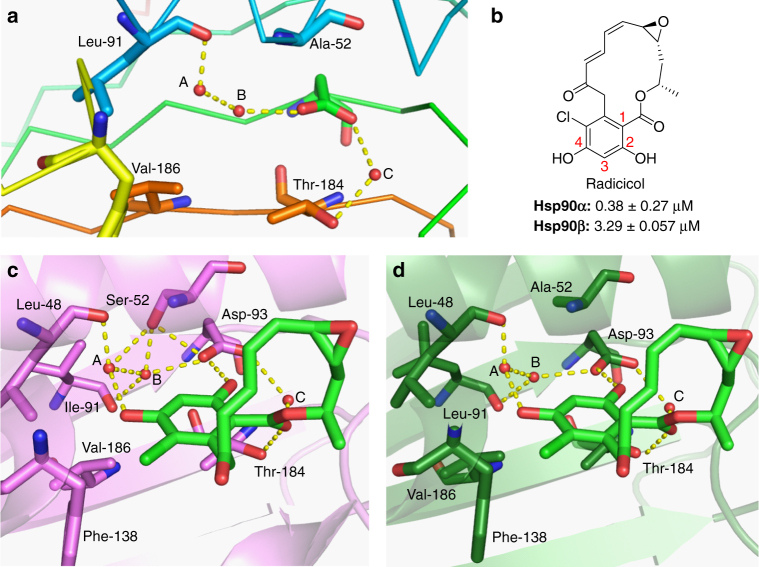
A water-mediated network of hydrogen bonds. **a** Three conserved water molecules at the bottom of the N-terminal ATP-binding site. **b** Structure of the known Hsp90 N-terminal inhibitor radicicol, which has been numbered for clarity. **c** Modeling of radicicol into the N-terminal ATP-binding site of Hsp90α (PDB code: 2XAB), and **d** Hsp90β (PDB code: 1UYM)

Sequence alignment of the N-terminal ATP-binding domain of Hsp90α and Hsp90β reveals that Hsp90β contains Ala52 and Leu91 residues in lieu of Ser52 and Ile91, which are present in Hsp90α (Supplementary Fig. 1). As shown in Fig. 1a, there is a water-mediated network of hydrogen bonds that align at the bottom of the pocket surrounding the resorcinol ring of radicicol bound to each Hsp90 isoform (Fig. 1c, d, and Supplementary Fig. 2). Similar to other Hsp90 inhibitors, radicicol (Fig. 1b) exhibits pan-inhibitory activity. Thr184 and Asp93 (numbered for Hsp90β) produce hydrogen bonds with the carbonyl and 4-phenol of radicicol through three conserved water molecules. Overlay of the Hsp90α and Hsp90β co-crystal structures suggest these water molecules play different roles in each isoform as a consequence of the replacement of Ser52 with Ala52 in Hsp90β^31^. Therefore, modification to the 4-position of the resorcinol ring was sought to evaluate these subtle differences about the 3- and 4-positions of the resorcinol ring, as substituents at the 4-position of the resorcinol ring would create unfavorable steric interactions with the bulkier side chains conserved in Hsp90α (Ser52, Ile91) Grp94 (Val147), and Trap-1 (Ile156) binding pockets (Supplementary Fig. 2).

### Structure-activity relationships

**Fig. 2:**
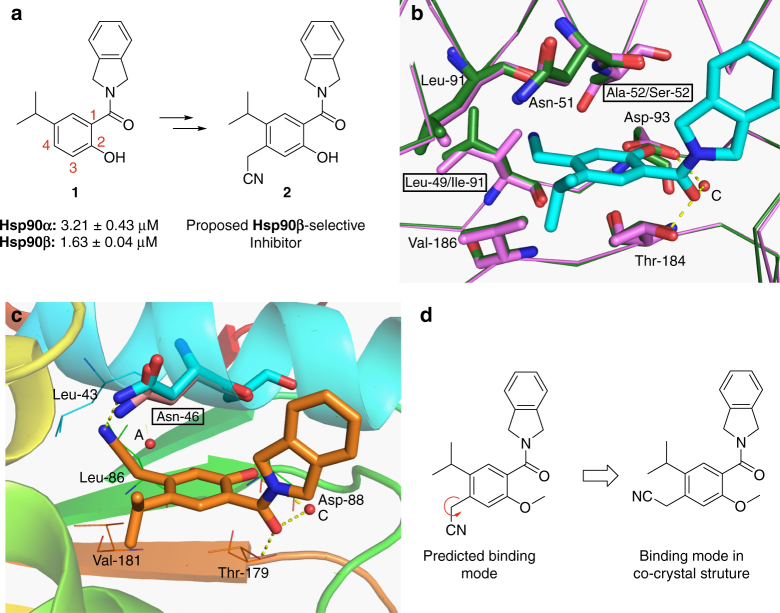
Proposed binding modes and co-crystal structures of Hsp90β-selective molecules. **a** A new scaffold for Hsp90β-selective inhibition. **b** Overlay of **2** docked into Hsp90α (PDB code 1UYM, colored green) with Hsp90β (PDB code: 2XAB). **c** Co-crystal structure of **2** bound to Hsp90β. **d** Compound **2** in 2-D binding mode. Movement of Asn46 allows for backward bending of nitrile; the previous position of Asn46 is represented in pink. Asn46 in co-crystal structure of **2** is represented in blue

**Fig. 3:**
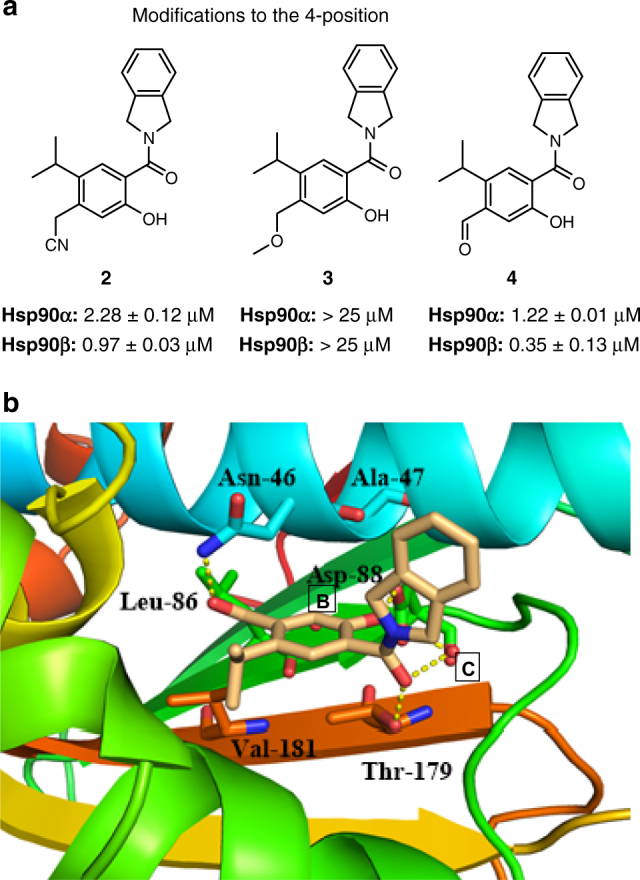
Modifications to the 4-position. **a** Modifications to the 4-position of the resorcinol ring and the corresponding *Kd* values. **b** Co-crystal structure of **4** bound to Hsp90β

Initial investigations began with compound **1** (Fig. 2a), which lacks the 4-phenol. Compound **1** (Supplementary Fig. 7) is derived from a known Hsp90 inhibitor and its Hsp90 inhibitory activity was confirmed via a fluorescence polarization assay (*Kd* = 1.63 μM for Hsp90β and 3.21 μM for Hsp90α, ~2-fold selective)^35,36^. The 2-phenol and the amide moiety of **1** mimic interactions manifested by the 2-phenol and lactone moiety present in the natural product, radicicol. The 5-isopropyl appendage serves to produce hydrophobic interactions with Phe138 and Val186 (Supplementary Fig. 3). Based on this data, substituents at the 4-position were investigated to determine selectivity and affinity against Hsp90β in silico. The cyanomethylene substituent was the first selected modification, and, as shown in Fig. 2b, the 4-cyanomethylene (**2**) was proposed to bind within the ATP-binding pocket of Hsp90β (green), but due to unfavorable steric interactions would not bind Hsp90α (magenta), Grp94, or Trap1 (Supplementary Fig. 4). Additionally, the nitrile was predicted to displace one of the conserved water molecules (water molecule A in Fig. 1a) in the binding pocket and increase the entropy of binding, while simultaneously establishing itself within the hydrogen bond network. Once prepared (Supplementary Fig. 8), compound **2** was evaluated in a fluorescence polarization assay to determine binding affinity^37^. Compound **2** exhibited a *Kd* of 2.27 and 0.97 μM against Hsp90α and Hsp90β, respectively, reflecting a (~2.5) 3-fold selectivity for Hsp90β. Additional derivatives of the 4-position were then prepared and included the 4-methoxymethylene and 4-formyl moieties (Fig. 3a). Introduction of the smaller 4-formyl group (**4**) led to both improved selectivity and affinity for Hsp90β (*Kd* = 310 nM versus 1.55 μM, ~4-fold selectivity); however, the 4-methoxymethylene-containing compound (**3**) did not bind either Hsp90 isoform at 10 μM (Fig. 3a). The binding modes of **2** and **4** were revealed by solution of the co-crystal structures bound to Hsp90β at 1.9 and 2.4 Å resolution, respectively. Examination of the co-crystal structures revealed an alternative binding mode for appendages at the 4-position, as the formyl and cyanomethylene appendages adopted a conformation wherein these moieties orient toward the back of the pocket, instead of the predicted forward orientation (Fig. 2b). In fact, Asn46 shifted 0.6 Å to accommodate the back-pointing cyanomethylene group (Fig. 2c, d), which established a new binding mode and resulted in hydrogen bonding interactions between the nitrile and Asn46 upon displacement of the conserved water molecule, B. The carbonyl of **4** bound in the expected conformation and produced hydrogen bonding interactions with Asn46, while simultaneously displacing conserved water molecule A (Fig. 3b).

**Fig. 4:**
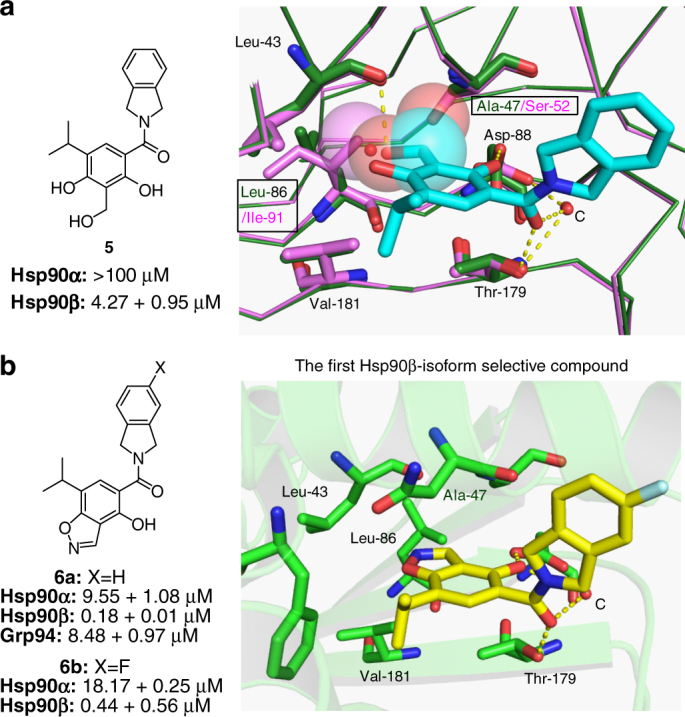
Identification of an Hsp90β-selective inhibitor. **a** Proposed binding of **5** to Hsp90α (magenta) and Hsp90β (green). **b** Apparent *Kd* of **6a** and **b** determined by fluorescence polarization and the co-crystal structure of **6b** bound to Hsp90β

While an increase in affinity was observed, modifications at the 4-position did not produce increased selectivity. Therefore, substituents were introduced at the 3-position to probe the subtle differences between Hsp90α (Ser52, Ile91) and Hsp90β (Ala52, Leu91). Molecular modeling studies suggested that the inclusion of a hydroxymethylene group at the 3-position would enhance selectivity for Hsp90β (Fig. 4a), as the methylene and hydroxyl groups would produce detrimental steric interactions with Ser52 and Ile91 in Hsp90α. Similarly, introduction of a benzyl alcohol would produce unfavorable interactions with Val148 and prohibit Grp94 binding (Supplementary Fig. 5). Furthermore, the hydroxyl group could displace one or both conserved water molecules in this region (water molecules A and B in Fig. 1a) and provide an entropic driving force. Upon preparation of **5** (Supplementary Fig. 10), it was evaluated in a fluorescence polarization assay and found to manifest an apparent *Kd* of 4.27 μM for Hsp90β. More importantly, it manifested selectivity (>25-fold) over the other isoforms. The co-crystal structure of **5** bound to Hsp90β was solved (Supplementary Fig. 6), which confirmed that the benzyl alcohol displaced both conserved water molecules, A and B, and participated in hydrogen bonding interactions with the backbone of Leu43.

Since flexibility associated with the hydroxymethylene group present in **5** is entropically disfavored, a ring-constrained variant was sought to minimize the entropic penalty, while simultaneously enhancing affinity. Using the co-crystal structure of **5** bound to Hsp90β, it was envisioned that the introduction of a heterocyclic ring system that joined both the 3- and 4-positions of the resorcinol ring in the form of benzoisoxazole would serve to illicit the desired interactions, while continuing to displace both of the conserved water molecules, A and B, as observed with **5** (Supplementary Fig. 6). Therefore, benzoisoxazole **6a** and the fluoroisoindoline analog **6b** were synthesized (Supplementary Figs. 11 and 12) and found to manifest an apparent *Kd* of 180 nM against Hsp90β, while exhibiting ~50-fold selectivity over Hsp90α and Grp94. The co-crystal structure of **6b** confirmed our hypothesis, as both water molecules A and B were displaced upon binding (Fig. 4b).

### In vitro evaluation

Once the Hsp90β-selective inhibitor, **6a** and herein referred to as **KUNB31**, was identified, cellular studies commenced to evaluate the effect of Hsp90β inhibition on cancer cell lines. The anti-proliferative activity manifested by **KUNB31** was evaluated against the cancer cell lines NCI H23 (non-small cell lung cancer), UC3 (bladder cancer), HT-29 (colon adenocarcinoma) cells, as well as non-cancerous HEK 293 (human embryonic kidney) cells. **KUNB31** manifested an IC_50_ of 6.74 ± 1.10 µM, 3.01 ± 0.56 µM, and 3.72 ± 0.34 µM against NCI H23, UC3, and HT-29 cancer cell lines, respectively, while requiring more than 100 µM against HEK-293 cells. NCI H23 and HT29 cells were then evaluated via Western blot analyses of known Hsp90α- and Hsp90β-dependent client proteins following treatment with **KUNB31** for 24 h. Prior studies identified CXCR4 and CDK-4/6 as Hsp90β-dependent client proteins, while the hERG channel, Erk-5, c-Raf and survivin represent Hsp90α-dependent substrates^38,39^.

**Fig. 5:**
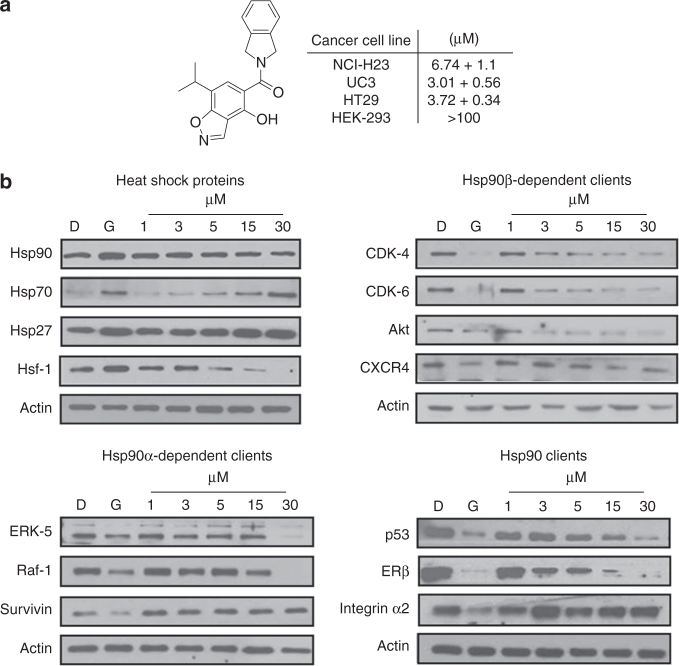
*In vitro* *evaluation of KUNB31*. **a** Anti-proliferation values of **6a** in immortalized cancerous (NCI-H23, UC3, HT29) and noncancerous (HEK-293) cells. **b** Representative Western blot analyses 24h after treatment with **6a** in HT29 cells at concentrations of 1, 3, 5, 15 and 30 μM. Negative and positive controls include DMSO (D) and geldanamycin (G) at 500 nM, respectively

Since Hsp90 inhibition induces the degradation of Hsp90-dependent substrates via the ubiquitin-proteosome pathway, the levels of both kinase and non-kinase Hsp90 clients were assessed via Western blot analysis. Known Hsp90 clients EGFR, HER2, CDK4, CDK6, CXCR_4_, Akt-1, c-Raf, Survivin, ERK-5 and Integrin α2 were analyzed following the administration of **KUNB31** to HT29 (colon adenocarcinoma grade II) cells. After a 24-h incubation with **KUNB31**, Hsp90β-dependent client proteins were reduced at concentrations that mirrored the cellular IC_50_ value, clearly linking cell viability to Hsp90β inhibition (Fig. 5a). In contrast, the level of Hsp90α-dependent clients, Raf-1, ERK-5, and survivin remained unaffected until higher concentrations. No client protein degradation was observed for the Grp94-dependent client, Integrin α2. Levels of clients like HER-2 and EGFR, which do not appear to be isoform dependent, decrease around 5 μM. Interestingly, levels of both Hsf-1 and Hsp90 decreased, which provides evidence that selective inhibition of Hsp90β does not increase Hsp90 levels as observed with the pan-Hsp90 N-terminal inhibitor, geldanamycin. However, levels of Hsp27 and Hsp70 were induced at higher concentrations.

Hsp90 inhibitors that have undergone clinical evaluation bind the Hsp90 N-terminal ATP-binding pocket and exhibit pan-inhibitory activity. Therefore, the development of Hsp90 isoform-selective inhibitors represents a new paradigm for cancer treatment and provides a mechanism by which isoform-dependent substrates can be elucidated and then selectively targeted for degradation. On-target toxicities that result from pan-inhibition of Hsp90 are not well understood, but may be overcome through inhibition of individual isoforms. Therefore, the development of isoform-selective inhibitors such as those disclosed herein can elucidate the roles played by each isoform as well as identify isoform-dependent substrates that contribute to various diseases while simultaneously diminishing the liabilities associated with pan-inhibition. Since the development of isoform-selective inhibitors of enzymes that are > 95% identical represents one of the greatest challenges in modern medicinal chemistry, the discovery of such compounds represents a significant advancement and lays the foundation for new therapeutic opportunities. Only a few successful examples of isoform selective inhibitors have been reported in the literature and, generally, those rely upon the presence of amino acids that contain nucleophilic side chains^40^. In contrast, by utilizing co-crystal structures of the known Hsp90 inhibitor, radicicol, bound to both Hsp90α and Hsp90β, a platform was developed on which the first Hsp90β isoform-selective inhibitor was rationally designed. Although only two amino acids differ between Hsp90α and Hsp90β, perturbation of the conserved water molecules that reside within this region led to the discovery of a small molecule that selectively inhibits the Hsp90β isoform. Compound **KUNB31**, which manifests 50-fold selectivity for Hsp90β, also manifests selective activity against cancer cells. The data presented herein provide the first evidence that selective inhibition of Hsp90β alters the heat shock response and prevents Hsp90 induction, which represents a serious detriment associated with pan-inhibitors of Hsp90. Furthermore, compound **KUNB31** can be used to validate isoform-selective inhibition as a viable approach toward cancer treatment while enabling the identification of specific roles played by Hsp90β in other diseases.

Traditional approaches to Hsp90 inhibition have relied upon pan-inhibition of all four Hsp90 isoforms. In an effort to advance new paradigms for Hsp90 inhibition, small-molecule inhibitors that manifest selective inhibition against individual Hsp90 isoforms were sought. The first N-terminal isoform-selective inhibitors of Hsp90β, particularly **KUNB31**, have been identified and shown to exhibit low micromolar anti-proliferative activity. Furthermore, compound **KUNB31** induced the degradation of select Hsp90β-dependent clients without concomitant induction of Hsp90 levels, providing a distinct and alternative mechanism for the treatment of cancer. In conclusion, the first Hsp90β N-terminal isoform-selective inhibitor has been discovered and preliminary biological studies indicate an opportunity to circumvent some of the challenges associated with the pan-Hsp90 inhibitors undergoing clinical evaluation.

## Methods

### Florescence polarization

Assay was performed in 96-well format in black, flat-bottom plates (Santa Cruz Biotechnology) with a final volume of 100 μL. Twenty-five microliters of assay buffer (20 mM HEPES, pH 7.3, 50 mM KCl, 5 mM MgCl_2_, 20 mM Na_2_MoO_4_, 2 mM DTT, 0.1 mg/mL BGG, and 0.01% NP-40) containing 6 nM FITC-GDA (fluorescent tracer, stock in DMSO and diluted in assay buffer) and 50 μL of assay buffer containing 10 nM of either Grp94 or Hsp90α were added to each well. Compounds were tested in triplicate wells (1% DMSO final concentration). For each plate, wells containing buffer only (background), tracer in buffer only (low polarization control), and protein and tracer in buffer with 1% DMSO (high polarization control) were included. Plates were incubated at 4 °C with rocking for 24 h. Polarization values (in mP units) were measured at 37 °C with an excitation filter at 485 nm and an emission filter at 528 nm. Polarization values were correlated to % tracer bound and compound concentrations. The concentration at which the tracer was 50% displaced by the inhibitor was determined using Graphpad Prism.

### Anti-proliferation assay for HEK-293, HT29 and NCI-H23 cells

Cells were grown to confluence and seeded at 2000 cells/well/0.1 mL in a 96-well plate and placed back in the incubator for 24 h. Compounds or vehicle were administered at the desired concentrations (1% DMSO final concentration) and incubated for 72 h. The percentage of viable cells was determined using the MTS/PMS cell proliferation kit (Promega) per the manufacturer’s instructions. Cells treated with vehicle were normalized to 100% proliferation and values adjusted accordingly.

HT29 (ATCC® HTB-38™) cells are human colorectal adenocarcinoma isolated from a 44-year-old, female, Caucasian patient and purchased from ATCC in April 2016. NCI-HT23 (ATCC® CRL-5800™) are non-small-cell, human lung adenocarcinoma isolated from a 51-year-old, male, Black patient and purchased from ATCC in April 2016. No characterization or mycoplasma testing was conducted on these cell lines following their purchase.

### Anti-proliferation assay for UM-UC-3 cells

UM**-**UC-3 cells were grown to confluence and seeded at 2000 cells/well/0.1 mL in a 96-well plate and placed back in the incubator for 24 h. Compounds or vehicle was administered at the desired concentrations (1% DMSO final concentration) and incubated for 72 h. The percentage of viable cells was determined using the Cell-Titer-Glo Luminescent Cell Viability Kit (Promega) per the manufacturer’s instructions. Cells treated with vehicle were normalized to 100% proliferation and values adjusted accordingly.

UM-UC-3 cells (ATCC® CRL-1749™) are bladder adenocarcinoma isolated from a male patient. No characterization or mycoplasma testing was conducted on these cell lines following their purchase.

### Western blot for UM-UC-3 cells

UM**-**UC-3 cells were harvested in cold PBS and lysed with RIPA buffer: 50 mM Tris-HCl pH 7.5, 150 mM NaCl, containing 0.1% SDS, 1% Igepal, 1% sodium deoxycholate, protease and phosphatase inhibitor cocktail (Sigma-Aldrich, Inc., St. Louis, MO) by three freeze-thaw cycles using liquid nitrogen and a 37 °C water bath. Protein concentration was determined using DC Protein Assay (Bio-Rad Laboratories, Hercules, CA). Equal amounts of protein (20 μg) were loaded on a Novex E-PAGETM 8% protein gel (Life Technologies), transferred to a nitrocellulose membrane by Novex iBlotR Gel Transfer system (Invitrogen, Carlsbad, CA), blocked in TBS-T containing 5% milk, and probed with primary antibodies (1:1000 dilution). Membranes were incubated with a horseradish peroxidase-conjugated secondary antibody, developed and visualized with Li-COR Odyssey Image system. All Western blots were probed for the loading control β-actin.

### Western blot for NCI-H23 cells

The NCI-H23 cells were harvested in cold PBS and lysed with mammalian protein extraction reagent (MPER, Pierce) lysis buffer containing protease and phosphatase inhibitors (Roche) on ice for 1 h. Lysates were clarified at 15,000 g for 20 min at 4 °C. Protein concentrations were determined using the Qubit protein quantification assay kit per the manufacturer’s instructions (ThermoFisher). Equal amounts of protein (2.5–20 μg) were electrophoresed under reducing conditions (10% acrylamide gel), transferred to a polyvinylidene fluoride membrane (PVDF), and immunoblotted with the corresponding specific antibodies. Membranes were incubated with an appropriate horseradish peroxidase-labeled secondary antibody, developed with a chemiluminescent substrate, and visualized. Data were first converted to 8-bit images in ImageJ, then Image Studio Lite Ver. 5.2 or Li-COR Odyssey Image Studio Ver 4.0 was used to perform densitometry. All proteins were normalized to actin and then DMSO, and the relative densities were reported.

### Co-crystal structure

His6-tagged human Hsp90β N-terminal domain (amino acids 1–218) was cloned into a modified pET vector, over-expressed in *Escherichia coli* BL21 DE3 cells and purified by Ni-NTA chromatography. The tag was cleaved using TEV protease, followed by a second subtracting Ni-NTA chromatography to remove the TEV and the his-tag moiety. The flow-through containing the cleaved protein was then concentrated and further purified via Superdex 200 size exclusion chromatography in 20 mM Tris-HCl, 150 mM NaCl, pH 7.8. Protein-inhibitor complexes were formed by mixing 15 mg/mL of Hsp90β NT with each inhibitor, at 1.5–2.0 mM final drug concentrations, and incubating at 4 °C for 1 h. Co-crystallization drops were set up at room temperature using 1:1 protein/drug to reservoir buffer of 30% PEG 8,000, 0.2 M sodium acetate, and 0.1 sodium cacodylate pH 6.5. Crystals appeared in 1–2 days and were harvested in a cryo-buffer containing 20% glycerol added to the reservoir buffer with each respective inhibitor at 2 mM.

### Co-crystal structure determination

Data collection was done at the beamline 19-ID at the Advanced Photon Source (APS), Argonne National Laboratory. The structure was solved by molecular replacement method using Phaser (28) with the structure of Hsp90-beta (PDB code 1UYM) as the model template. PHENIX program (29) was used for the refinement, and Coot (30) was used for the iterative manual model building. Translation, libration and screw-rotation displacement (TLS) groups used in the refinement were defined by the TLMSD server (31). The current models are of good geometry and refinement statistics (Supplementary Table 1). All structure factors and pdbs were deposited with RCSB.org with pdb accession codes 5UC4, 5UCH, 5UCI and 5UCJ.

### X-ray crystallography study for C19H18N2O3, v87c(1)

A set of unique diffraction data (4438 0.5°-wide ω- or ϕ-scan frames with scan times of 3–6 s) were collected (Supplementary Fig. 1) at 100(2)K for a single-domain crystal using monochromated CuKa radiation (*l* = 1.54178 Å) on a Bruker Proteum Single Crystal Diffraction System equipped with dual CCD area detectors. Data collection utilized a Platinum 135 CCD detector with a crystal-to-detector distance of 8.0 cm and Helios high-brilliance multilayer optics. X-rays were provided with a Bruker MicroStar microfocus Cu rotating anode X-ray source operating at 45 kV and 60 mA. The integrated data (Supplementary Fig. 2) were corrected empirically for variable absorption effects using equivalent reflections. The Bruker software package SHELXTL was used to solve the structure using “direct methods” techniques. All stages of weighted full-matrix least-squares refinement were conducted using Fo2 data with the SHELXTL XL v2014 software package (Supplementary Fig. 3). All hydrogen atoms were located from a difference Fourier and refined in least-square refinement cycles as independent isotropic atoms. All non-hydrogen atoms were included in the structural model with anisotropic thermal parameters. Final crystallographic details are summarized in Supplementary Table 1.

### Data availability

The data that support the findings of this study are available from the corresponding author upon reasonable request. All structure factors and PDBs were deposited with RCSB.org with PDB accession codes 5UC4, 5UCH, 5UCI and 5UCJ.

## Electronic supplementary material

Supplementary Information
