# Supplementary material for: Structure-guided design of an Hsp90β N-terminal isoform-selective inhibitor
Source: Nat Commun. 2018 Jan 30;9:425. doi: 10.1038/s41467-017-02013-1 (PMC5789826; doi:10.1038/s41467-017-02013-1)
Supplement: Supplementary file 1 — Supplementary Information [file 41467_2017_2013_MOESM1_ESM.pdf]

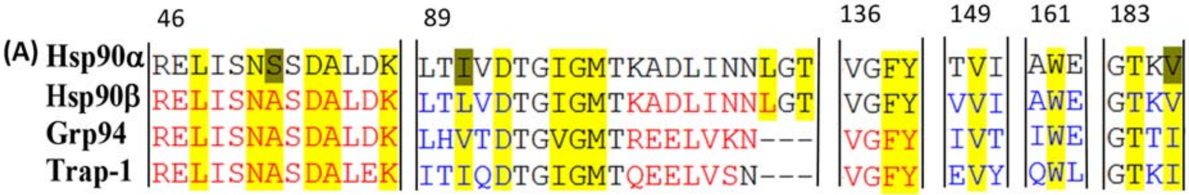

Supplementary Figure 1: (A) Sequence alignment of Hsp90 isoforms. Residues highlighted in yellow comprise N-terminal ATP binding site. Residues in blue boxes indicate the amino acid difference between Hsp90 isoforms.

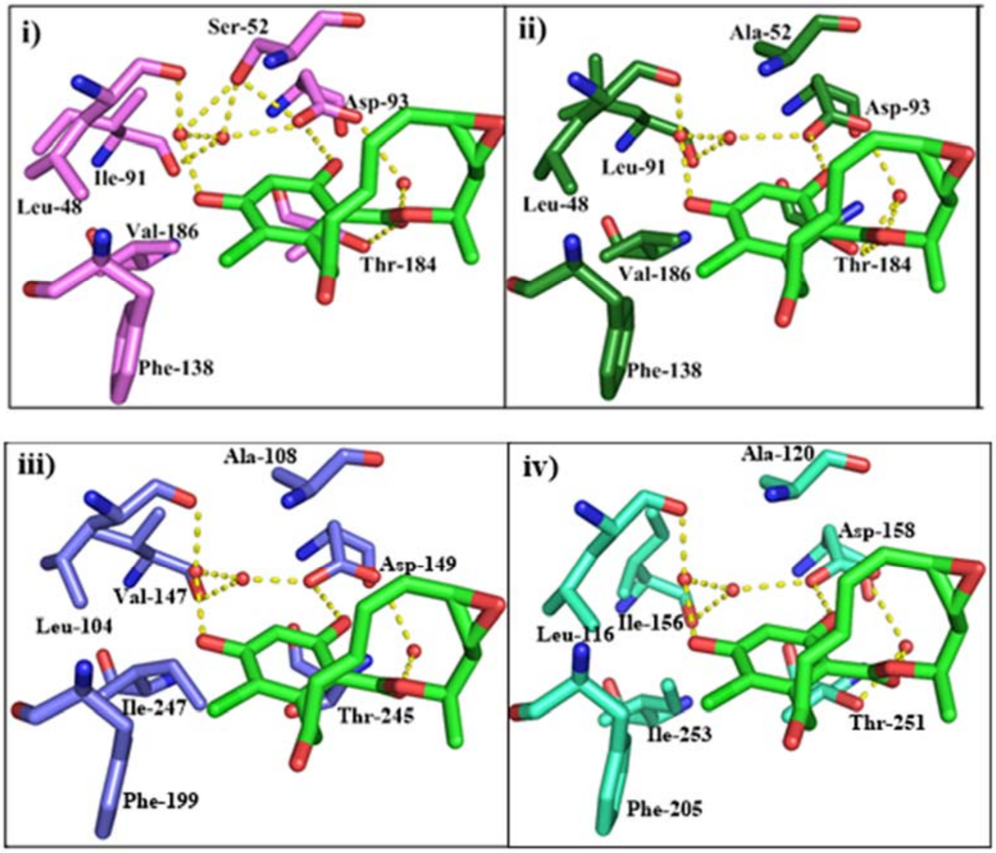

Supplementary Figure 2: Modeling of Radicicol into N-terminal ATP binding site of Hsp90 isoforms, i) Hsp90α (PDB code: 2XAB) ii) Hsp90β (PDB code: 1UYM) iii) Grp94 (PDB code: 4NH9) iv) Trap-1(PDB code: 4Z1F)

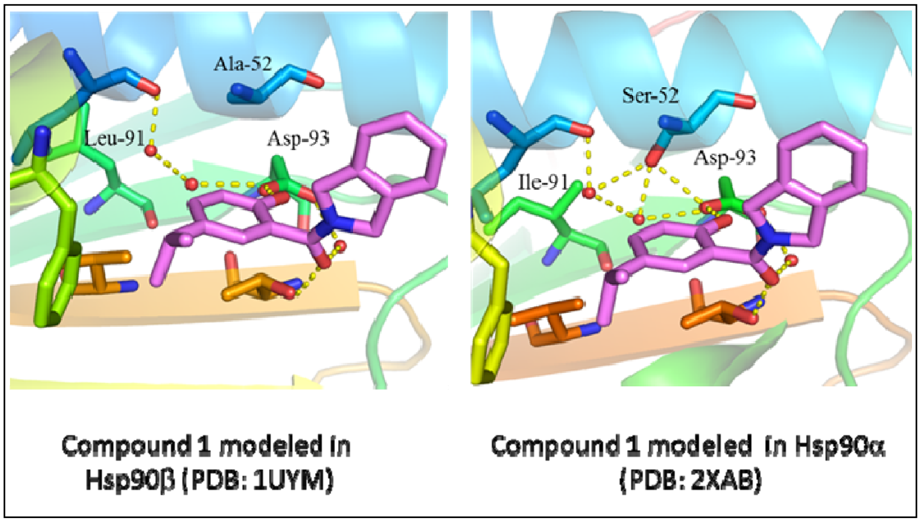

Supplementary Figure 3: Modeling of compound 1 in Hsp90β (PDB code: 1UYM) and Hsp90α binding pocket (PDB code: 2XAB).

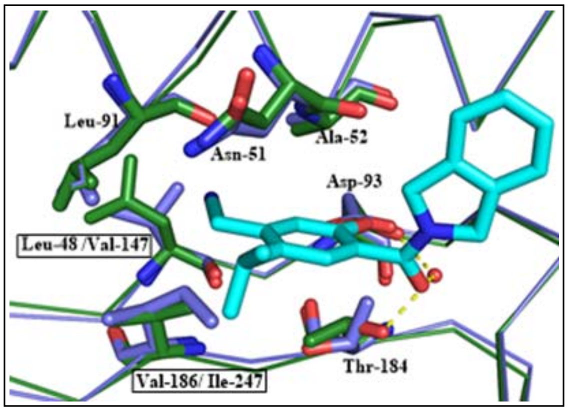

Supplementary Figure 4: Overlay of 2 docked into Hsp90β (PDB code 1UYM, colored green) with N-terminal ATP binding site of Grp94 (PDB code: 2GFD;

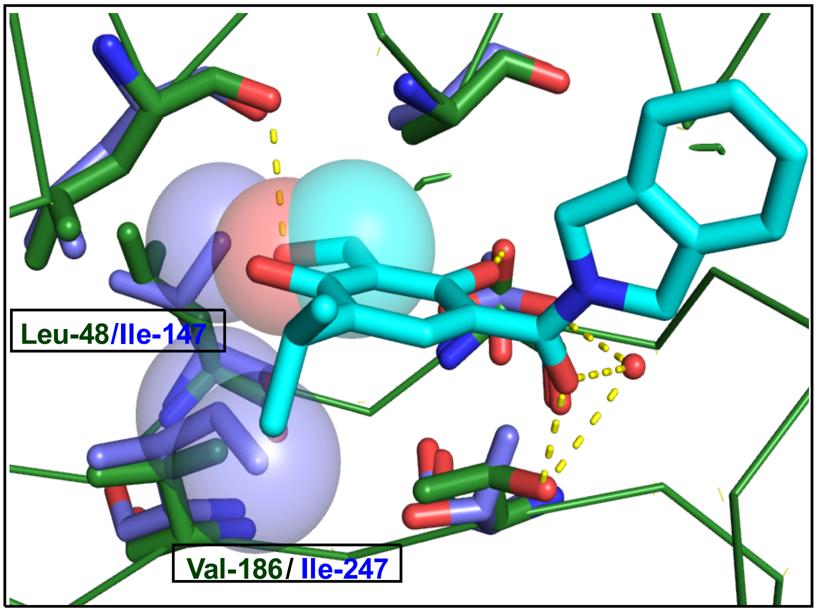

Supplementary Figure 5: Proposed binding of 5 in an overlay of Grp94 (magenta) and Hsp90β (green)

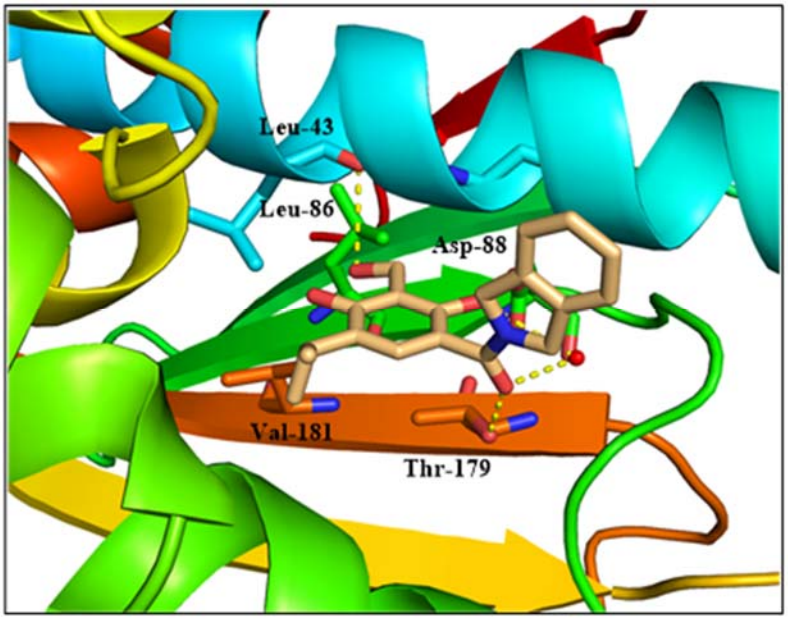

Supplementary Figure 6: Co-crystal structure of compound 5 bound to Hsp90β.

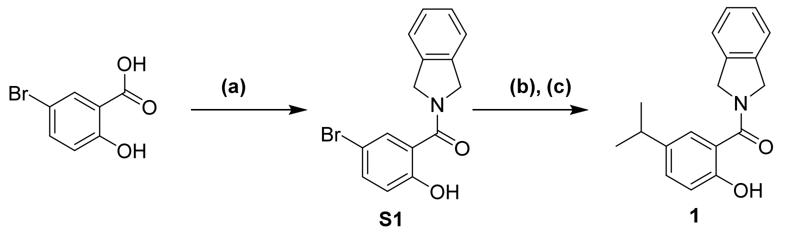

Reagents: (a) isoindoline hydrochloride, EDCI, HOBt, N,N-diisopropylethylamine; (b) potassium isopropenyltrifluoroborate, $Pd(PPh_3)$, Cs_2CO_3; (c) Pd/C-H_2

Supplementary Figure 7: Synthetic scheme for compound 1.

(5-Bromo-2-hydroxyphenyl)(isoindolin-2-yl)methanone (S1):[4-5] 1-Ethyl-3-(3-dimethylaminopropyl)carbodiimide (5.20 g, 27.64 mmol, 2.0 eq.) was added to a stirred solution of 83 (3.0 g, 13.82 mmol, 1 eq.), isoindoline hydrochloride (3.22 g, 20.73 mmol, 1.5 eq.), 1-hydroxybenzotriazole (3.73 g, 27.64 mmol, 2 eq.) *N,N*-diisopropylethylamine (4.81 mL, 27.64 mmol, 2.0 eq.) in dichloromethane (150 mL) at 0 °C. The resulting solution was stirred at rt for 14 h before quenching with saturated sodium bicarbonate solution (120 mL). The organic layer was washed with 1 M hydrochloric acid solution (120 mL) and saturated sodium chloride solution (120 mL), dried over sodium sulfate, filtered, and concentrated. The residue was purified by flash chromatography (SiO_2, 1:4 hexanes/ethyl acetate) to afford 84 (3.24 g,77.9 %) as a white amorphous solid. ^{1}H NMR (400 MHz, $CDCl_3$) δ 10.92 (s, 1H), 7.73 (d, J = 2.2 Hz, 1H), 7.48 (dd, J = 8.8, 2.4 Hz, 1H), 7.34 (s, 4H), 6.94 (d, J = 8.8 Hz, 1H), 5.10 (s, 4H). ^{13}C NMR (100MHz, $CDCl_3$) δ 169.4, 159.4, 136.1 (2), 134.8, 130.5, 128.2 (2), 122.8 (2), 120.2, 118.7 110.2, 55.8, 53.3. HRMS (ESI+) *m/z* [M + H^+] calcd for $C_{15}H_{13}BrNO_2$, 318.0130, found 318.0119.

(2-Hydroxy-5-isopropylphenyl)(isoindolin-2-yl)methanone (1): A biotage microwave vial was charged with 5-bromo-2-hydroxybenzoic acid (1.0 g, 3.42 mmol, 1 eq.), Tetrakis(triphenylphosphine)palladium (0) (277 mg, 0.24 mmol, 0.1 eq.), cesium carbonate (2.4 g, 7.4 mmol, 3 eq.), and potassium isopropenyltrifluoroborate (427 mg, 2.88 mmol, 1.2 eq.). The tube

was sealed with a cap lined with a disposable Teflon septum. The tube was evacuated and purged with nitrogen (3 times), before the addition of 2-propanol (17 mL) by syringe. The resulting mixture was heated at 100 °C for 6 h, cooled to rt, and filtered through a small pad of celite (elution with ethyl acetate). Solvent was removed and the residue purified by flash chromatography (SiO_2, 1:4 ethyl acetate/hexanes) to afford (2-hydroxy-5-(prop-1-en-2-yl)phenyl)(isoindolin-2-yl)methanone, which was used further as obtained. Palladium on carbon (10%) was added to a solution of (2-hydroxy-5-(prop-1-en-2-yl)phenyl)(isoindolin-2-yl)methanone in ethyl acetate (25 mL). The suspension was stirred for 16 h under a hydrogen atmosphere before it was filtered through a pad of celite and eluted with EtOAc (20 mL). The eluent was concentrated to afford 1 (652 mg, 67.5 %) as a white amorphous solid. ^{1}H NMR (400 MHz, $CDCl_3$) δ 10.44 (s, 1H), 7.40 (d, J = 2.2 Hz, 1H), 7.28 (s, 4H), 6.92 (d, J = 8.5 Hz, 1H), 5.06 (s, 4H), 2.88 (hept, J = 7.0 Hz, 1H), 1.24 (d, J = 6.9 Hz, 6H). ^{13}C NMR (100 MHz, $CDCl_3$) δ 171.4, 157.9, 138.8, 136.1 (2), 131.5, 128.0 (2), 125.7, 122.8 (2), 118.0, 117.1, 55.8, 53.5, 33.6, 24.4 (2). HRMS (ESI+) *m/z* [M + H^+] calcd for $C_{18}H_{20}NO_2$, 282.1494, found 282.1483.

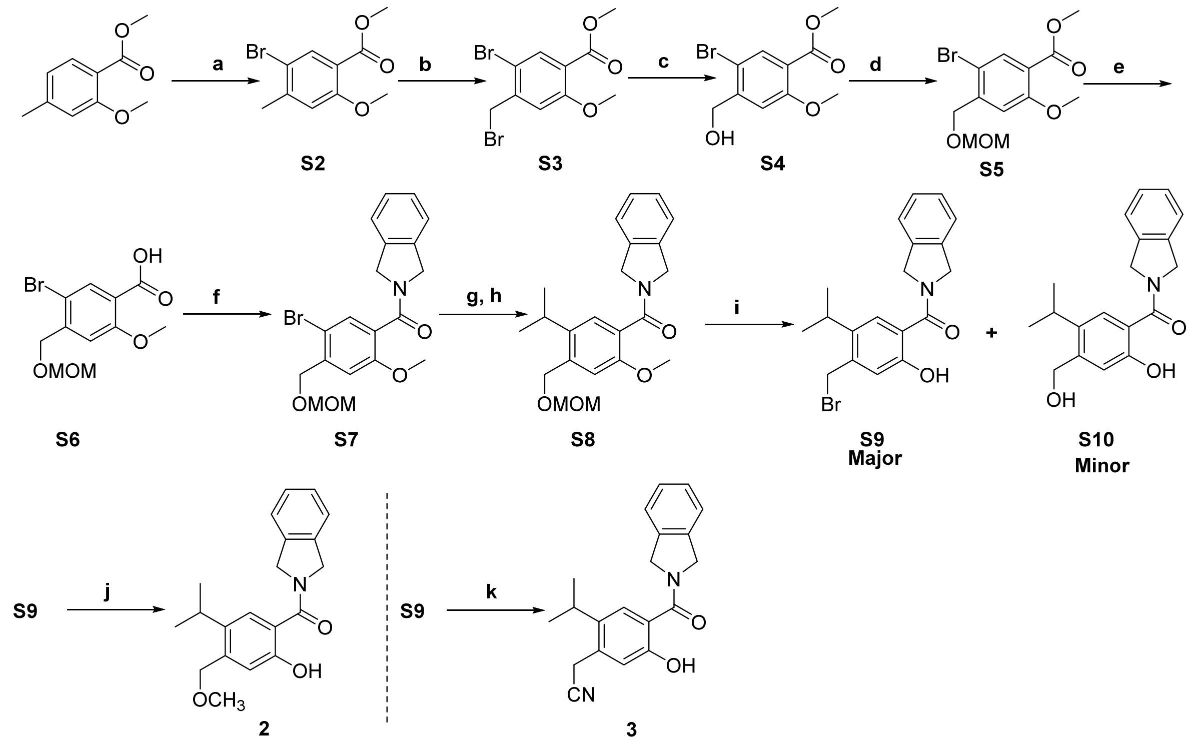

Reagents: (a) Br_2 ; (b) NBS, AIBN; (c) $CaCO_3$; (d) MOMCl, N,N-diisopropylethylamine; (e) LiOH; (f) isoindoline hydrochloride, EDCI, HOBt, N,N-diisopropylethylamine; (g) potassium isopropenyltrifluoroborate, $Pd(PPh_3)_4$, Cs_2CO_3; (h) Pd/C,H_2; (i) BBr_3; (j) CH_3OH; (k) KCN, 18-crown 6.

Supplementary Figure 8: Synthetic scheme of compound 2 and 3. Detailed synthetic methods and characterization provided below:

Methyl 5-bromo-2-methoxy-4-methylbenzoate (S2): A solution of bromine (3.12 mL, 60.9 mmol, 1.05 eq.) in chloroform (27 mL) was added dropwise to a solution of methyl 2-methoxy-4-methylbenzoate (10.0 g, 55.4 mmol, 1.0 eq.) in chloroform at 0 °C (250 mL). The resulting mixture was stirred at rt for 4 h before quenching with 10% aqueous solution of sodium thiosulfate (200 mL). The organic layer was washed with saturated sodium bicarbonate solution (2 × 200 mL) and saturated sodium chloride solution (200 mL), dried over anhydrous sodium sulfate, filtered, and concentrated. The residue was purified by flash chromatography (SiO_2, 1:9 ethyl acetate/hexanes) to afford S2 (12.81 g, 89.2 %) as a light brown amorphous solid. ^1H NMR (400 MHz, $CDCl_3$) δ 7.93 (dt, J = 7.1, 4.0 Hz, 1H), 6.82 (t, J = 4.0 Hz, 1H), 3.97 – 3.76 (m, 6H), 2.38 (dt, J = 6.5, 4.0

Hz, 3H). ^{13}C NMR (125 MHz, $CDCl_3$) δ 165.2, 158.5, 144.0, 135.2, 118.9, 114.8, 114.6, 56.3, 52.2, 23.7. HRMS (ESI+) *m/z* [M + H^+] calcd for $C_{10}H_{12}BrO_3$, 258.9969, found 258.9973.

Methyl 5-bromo-4-(bromomethyl)-2-methoxybenzoate (S3): A solution of S2 (12.8g, 48.8 mmol), N-bromosuccinimide (9.68 g, 54.78 mmol, 1.1 eq.), azobisisobutyronitrile (1.64 g, 9.96 mmol, 0.2 eq.) in carbon tetrachloride was heated at 70 ºC. After 14 h, solvent was removed and the residue purified by flash chromatography (SiO_2, 1:9 ethyl acetate/hexanes) to afford S3 (13.92 g, 71.3 %) as a white amorphous solid. ^1H NMR (500 MHz, $CDCl_3$) δ 7.91 (s, 1H), 7.60 (s, 1H), 7.00 (s, 1H), 3.99 (s, 3H), 3.90 (s, 3H). ^{13}C NMR (125 MHz, $CDCl_3$) δ 164.7, 159.0, 144.8, 135.5, 123.0, 114.6, 109.5, 56.7, 52.7, 39.3. HRMS (ESI+) *m/z* [M + H^+] calcd for $C_{10}H_{11}Br_2O_3$, 336.9075, found 336.9079.

Methyl 5-bromo-4-(hydroxymethyl)-2-methoxybenzoate (S4):[6] Calcium carbonate (11.3 g, 113.4 mmol, 3 eq.) was added to a solution of S3 (12.8 g, 37.8 mmol, 1 eq.) in dioxane (100 mL) and water (100 mL). The resulting mixture was heated at 120 °C in a sealed tube for 16 h. The reaction mixture was cooled to rt, filtered, and concentrated. The residue was purified by flash chromatography (SiO_2, 1:5 ethyl acetate/hexanes) to afford S4 (8.2 g, 79.2%) as a colorless amorphous solid. ^1H NMR (500 MHz, $CDCl_3$) δ 7.9 (s, 1H), 7.2 (d, J = 0.9 Hz, 1H), 4.7 (dd, J = 5.7, 1.0 Hz, 2H), 3.9 (s, 3H), 3.9 (s, 3H). ^{13}C NMR (125 MHz, $CDCl_3$) δ 165.5, 159.0, 145.7, 135.3, 120.0, 111.9, 111.3, 64.8, 56.5, 52.5. HRMS (ESI+) *m/z* [M + H^+] calcd for $C_{10}H_{12}BrO_4$, 274.9919, found 274.9923.

Methyl 5-bromo-2-methoxy-4-((methoxymethoxy)methyl)benzoate (S5): A solution of S4 (3.2 g, 11.63 mmol, 1.0 eq.) in dichloromethane (116 mL) was cooled to 0 °C before the addition of *N,N*-diisopropylethylamine (12.13 mL, 69.79 mmol, 6.0 eq.) and 6M solution of chloromethoxymethyl ether (11.8 ml, 69.79 mmol, 6.0 eq.). The reaction was allowed to reach at rt and stirred for 14 h

before quenching with saturated sodium bicarbonate solution (60 mL). The aqueous layer was extracted with dichloromethane (2 × 60 mL) and the combined organic layers were washed with saturated sodium chloride solution (150 ml), dried over anhydrous sodium sulfate, filtered, and concentrated. The residue was purified by flash chromatography (SiO_2, 1:49 acetone/dichloromethane) to afford S5 (2.82 g, 76.1%) as a colorless oil. ^{1}H NMR (400 MHz, $CDCl_3$) δ 7.97 (s, 1H), 7.18 (d, J = 0.9 Hz, 1H), 4.80 (s, 2H), 4.64 (d, J = 0.9 Hz, 2H), 3.93 (s, 3H), 3.89 (s, 3H), 3.44 (s, 3H). ^{13}C NMR (100 MHz, $CDCl_3$) δ 165.4, 158.9, 143.4, 135.4, 120.3, 112.4, 111.8, 96.6, 68.9, 56.6, 55.9, 52.4. HRMS (ESI+) *m/z* [M + H^+] calcd for $C_{12}H_{16}BrO_5$, 319.0181, found 319.0187.

5-Bromo-2-methoxy-4-((methoxymethoxy)methyl)benzoic acid (S6): Lithium hydroxide monohydrate (5.37 g, 128.0 mmol, 10.0 eq.) was added to a solution of S5 (4.08 g, 12.8 mmol, 1 eq.) in a solvent mixture of tetrahydrofuran (43 mL), water (43 mL), methanol (43 mL). The resulting mixture was stirred at rt for 16 h and concentrated. The residue was treated with 1 M hydrochloric acid and pH was adjusted to 2. The resulting suspension was extracted with ethyl acetate (3 × 100 mL), the combined organic layers were washed with saturated sodium chloride solution, dried over anhydrous sodium sulfate, filtered and concentrated to afford S6 (3.26 g, 83.2 %) as a light brown solid. ^{1}H NMR (500 MHz, $CDCl_3$) δ 10.59 (s, 1H), 8.33 (s, 1H), 7.30 (s, 1H), 4.83 (s, 2H), 4.67 (d, J = 0.9 Hz, 2H), 4.12 (s, 3H), 3.46 (s, 3H). ^{13}C NMR (125 MHz, $CDCl_3$) δ 164.1, 157.5, 145.5, 137.1, 117.8, 113.9, 111.6, 96.7, 68.7, 57.3, 56.0. HRMS (ESI+) *m/z* [M + H^+] calcd for $C_{11}H_{14}BrO_5$, 305.0025, found 305.0028.

(5-Bromo-2-methoxy-4-((methoxymethoxy)methyl)phenyl)(isoindolin-2-yl)methanone (S7): 1-Ethyl-3-(3-dimethylaminopropyl)carbodiimide (1.25g, 6.54 mmol, 2.0 eq.) was added to a stirred solution of S6 (1.0 g, 3.27 mmol, 1 eq.), isoindoline hydrochloride (663 mg, 4.26 mmol, 1.3 eq.),

1-hydroxybenzotriazole (1.0 g, 6.54 mmol, 2 eq.) N-,N-diisopropylethylamine (1.72 mL, 9.81 mmol, 3.0 eq.) in dichloromethane (33 mL) at 0 °C. The resulting solution was stirred at rt for 14 h before quenching with saturated sodium bicarbonate solution (30 mL). The organic layer was washed with 1 M hydrochloric acid solution (30 mL) and saturated sodium chloride solution (30 mL), dried over sodium sulfate, filtered and concentrated. The residue residue was purified by flash chromatography (SiO_2, 1:3 hexanes/ethylacetate) to afford S7 (1.22 g, 91.8%) as a colorless oil. ^{1}H NMR (400 MHz, $CDCl_3$) δ 7.50 (s, 1H), 7.37 – 7.26 (m, 3H), 7.19 – 7.12 (m, 2H), 4.99 (s, 2H), 4.82 (s, 2H), 4.68 (s, 2H), 4.62 (s, 2H), 3.88 (s, 3H), 3.48 (s, 3H). HRMS (ESI+) *m/z* [M + H^+] calcd for $C_{11}H_{14}BrO_5$, 452.1072, found 452.1066.

Isoindolin-2-yl(5-isopropyl-2-methoxy-4-((methoxymethoxy)methyl)phenyl)methanone (S8): A biotage microwave vial was charged with S7 (1.0 g, 2.46 mmol, 1 eq.), Tetrakis(triphenylphosphine)palladium (0) (277 mg, 0.24 mmol, 0.1 eq.), cesium carbonate (2.4 g, 7.4 mmol, 3 eq.), and potassium isopropenyltrifluoroborate (427 mg, 2.88 mmol, 1.2 eq.). The tube was sealed with a cap lined with a disposable Teflon septum. The tube was evacuated and purged with nitrogen (3 times), before the addition of tetrahydrofuran (10.8 mL) and water (1.2 mL) by syringe. The resulting mixture was heated at 100 °C for 24 h, cooled to rt, and filtered through a small pad of celite (elution with ethyl acetate). Solvent was removed and the residue purified by flash chromatography (SiO_2, 1:3 ethyl acetate/hexanes) to afford isoindolin-2-yl(2-methoxy-4-((methoxymethoxy)methyl)-5-(prop-1-en-2-yl)phenyl)methanone, which was used further as obtained. Palladium on carbon (10%) was added to a solution of isoindolin-2-yl(2-methoxy-4-((methoxymethoxy)methyl)-5-(prop-1-en-2-yl)phenyl)methanone in ethyl acetate (25 mL). The suspension was stirred for 16 h under a hydrogen atmosphere before it was filtered through a pad of celite and eluted with EtOAc (20 mL). The eluent was concentrated to afford S8 (530 mg, 58.3

%) as a light brown amorphous solid. ^{1}H NMR (400 MHz, $CDCl_3$) δ 7.76 (d, *J* = 7.6 Hz, 1H), 7.60 (td, *J* = 7.6, 1.1 Hz, 1H), 7.48 (d, *J* = 7.7 Hz, 1H), 7.41 (t, *J* = 7.5 Hz, 1H), 7.21 (s, 1H), 6.94 (s, 1H), 4.94 (s, 2H), 4.67 (s, 2H), 4.63 (s, 2H), 3.70 (s, 3H), 3.37 (s, 3H), 3.05 (h, *J* = 6.8 Hz, 1H), 1.15 (s, 3H). ^{13}C NMR (100 MHz, $CDCl_3$) δ 168.7, 166.3, 155.0, 141.6, 139.0, 138.4, 134.1, 131.6, 128.7, 125.7, 125.4, 125.2, 123.7, 111.2, 96.0, 66.8, 56.1, 55.7, 48.7, 28.4, 24.0 (2). HRMS (ESI+) *m/z* [M + Na^+] calcd for $C_{22}H_{27}NO_4Na$, 392.1838, found 392.1838.

(4-(Bromomethyl)-2-hydroxy-5-isopropylphenyl)(isoindolin-2-yl)methanone (S9): 1 M solution of boron tribromide (1.22 mmol, 1.22 mL, 2 eq.), was added to a solution of 92 (200 mg, 0.61 mmol) in anhydrous dichloromethane (6.1 ml) at 0 ºC. The resulting mixture was stirred at rt for 14 h before quenching with saturate sodium bicarbonate solution (5 mL). The aqueous layer was extracted with dichloromethane (2 × 10 mL). The combined organic layers were washed with saturated sodium chloride solution (20 mL), dried over anhydrous sodium sulfate, filtered, and concentrated. The residue was purified by flash chromatography (SiO_2, 1:50 acetone/dichloromethane) to afford S9 (98 mg, 43.7 %) as a white solid. Additionally, compound S10 (38 mg, 20%) was also isolated. ^{1}H NMR (500 MHz, CD_2Cl_2) δ 7.53 (s, 1H), 7.32 (s, 4H), 6.94 (s, 1H), 5.07 (s, 5H), 4.53 (s, 2H), 3.27 (p, *J* = 6.8 Hz, 1H), 1.31 (d, *J* = 6.8 Hz, 5H). ^{13}C NMR (125 MHz, CD_2Cl2) δ 170.5, 157.3, 139.9 (2), 138.1 (2), 128.1, 126.3, 123.0, 119.4 (2), 119.1 (2), 118.5, 55.9 (2), 31.0, 24.3 (2). HRMS (ESI+) *m/z* [M + Na^+] calcd for $C_{19}H_{20}BrNO_2Na$, 374.756, found 374.0769.

(2-Hydroxy-4-(hydroxymethyl)-5-isopropylphenyl)(isoindolin-2-yl)methanone (S10): Obtained as a colorless solid (38 mg, 20%). ^{1}H NMR (400 MHz, $CDCl_3$) δ 10.53 (s, 1H), 7.53 (s, 1H), 7.32 (s, 4H), 7.07 (s, 1H), 5.10 (s, 4H), 4.76 (s, 2H), 3.17 – 3.22 (m, 1H), 1.26 – 1.30 (m, 6H). ^{13}C NMR (125 MHz, $CDCl_3$) δ 171.0, 158.0, 150.2, 143.1, 136.7, 136.4, 129.9, 129.1, 126.8, 126.6, 125.2,

116.9, 116.6, 62.9, 55.8, 53.4, 30.2, 24.5 (2). HRMS (ESI+) *m/z* [M + H$^+$] calcd for $C_{19}H_{22}NO_3$, 312.1600, found 312.1604.

2-(5-Hydroxy-4-(isoindoline-2-carbonyl)-2-isopropylphenyl)acetonitrile (2): Potassium cyanide (43 mg, 0.66 mmol, 5.0 eq.) was added to a solution of S9 (50 mg, 0.14 mmol, 1.0 eq.) and 18-crown-6 (71 mg, 0.27 mmol, 2.0 eq.) in *N,N*-dimethylformamide (2.0 mL) at rt. The reaction was stirred for 2 h, diluted with water (20 mL), extracted with ethyl acetate (2 × 20 mL). The combined organic layers were washed with saturated sodium chloride solution (30 mL), dried over anhydrous sodium sulfate, filtered, and concentrated. The residue was purified by flash chromatography (SiO_2, 1:3 ethyl acetate/hexanes) to afford 2 (39.9 mg, 92.5 %) as colorless oil. ^{1}H NMR (400 MHz, $CDCl_3$) δ 10.53 (s, 1H), 7.54 (s, 1H), 7.32 (d, *J* = 4.6 Hz, 4H), 7.03 (s, 1H), 5.09 (s, 4H), 3.74 (s, 2H), 3.07 (hept, *J* = 6.9 Hz, 1H), 1.32 (d, *J* = 6.8 Hz, 6H). ^{13}C NMR (100 MHz, $CDCl_3$) δ 170.5, 158.0, 136.5, 135.85 (2) 132.4, 128.1, 125.7 (2), 122.8, 118.6, 117.7, 117.5, 56.2 (2), 29.0, 24.1(2), 21.7. HRMS (ESI+) *m/z* [M + H$^+$] calcd for $C_{20}H_{21}N_2O_2$, 321.1603, found 321.1610.

(2-Hydroxy-5-isopropyl-4-(methoxymethyl)phenyl)(isoindolin-2-yl)methanone (3): Sodium methoxide (10.8 mg, 0.20 mmol, 2.5 eq.) was added to a solution of S9 (30 mg, 0.08 mmol, 1 eq.) in anhydrous methanol. The resulting mixture was heated at 60 ºC for 8h, before quenching with 1 M hydrochloric acid (2 mL), extracted with ethyl acetate (3 × 3 mL). The combined organic layers were washed with saturated sodium chloride solution, dried over sodium sulfate, filtered, and concentrated. The residue was purified by flash chromatography (SiO_2, 3:10 ethyl acetate/hexanes) to afford 3 (13 mg, 49.9 %) as a colorless oil. ^{1}H NMR (400 MHz, $CDCl_3$) δ 10.45 (s, 1H), 7.52 (s, 1H), 7.29 (d, *J* = 19.5 Hz, 4H), 7.02 (s, 1H), 5.09 (s, 4H), 4.50 (s, 2H), 3.43 (s, 3H), 3.14 – 3.46 (m, 1H), 1.28 (d, *J* = 6.9 Hz, 7H). ^{13}C NMR (100 MHz, $CDCl_3$) δ 170.8, 157.4,

140.4 (2), 137.0 (2), 127.8, 124.9(2), 122.6, 117.8 (2), 116.6, 72.1, 58.3 (3), 28.0, 24.2 (2). HRMS (ESI+) *m/z* [M + Na$^+$] calcd for $C_{20}H_{23}NO_3Na$, 348.1576, found 346.1567.

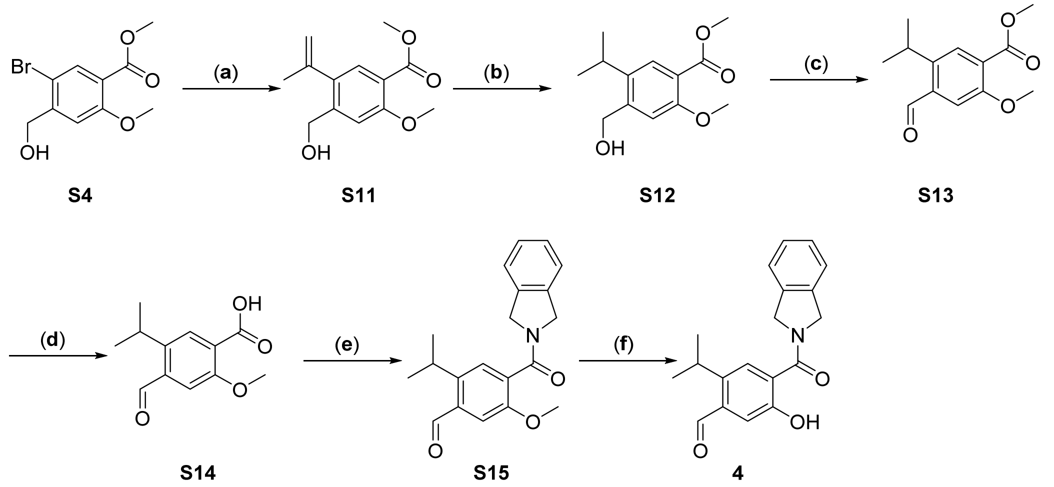

Supplementary Figure 9: Synthetic Scheme for compound 4. Detailed synthetic methods and characterization provided below:

Methyl 4-formyl-2-methoxy-5-(prop-1-en-2-yl)benzoate (S11): A biotage microwave vial was charged with S4 (0.75 g, 2.7 mmol, 1 eq.), tetrakis(triphenylphosphine)palladium(0) (312 mg, 0.27 mmol, 0.1 eq.), cesium carbonate (2.68 g, 8.25 mmol, 3 eq.), and potassium isopropenyltrifluoroborate (440 mg, 2.97 mmol, 1.2 eq.). The tube was sealed with a cap lined with a disposable teflon septum. The tube was evacuated and purged with nitrogen (3 times), before the addition of tetrahydrofuran (21.6 mL) and water (2.4 mL) by syringe. The resulting mixture was heated at 100 °C for 24 h, cooled to rt, and filtered through a small pad of celite (elution with ethyl acetate). Solvent was removed and the residue was purified by flash chromatography (SiO_2, 1:3 ethyl acetate/hexanes) to afford S11 as a colorless amorphous solid (580 mg, 90 %). ^{1}H NMR (400 MHz, $CDCl_3$) δ 7.57 (s, 1H), 7.14 (s, 1H), 5.19 (p, J = 1.7 Hz, 1H),

4.82 – 4.76 (m, 1H), 4.69 (s, 2H), 3.84 (d, *J* = 3.0 Hz, 6H), 2.72 (s, 1H), 2.06 – 1.94 (m, 3H). ^{13}C NMR (100 MHz, $CDCl_3$) δ 166.7, 158.4, 144.0, 143.3, 134.2, 131.3, 118.1, 116.1, 110.9, 62.5, 56.1, 52.1, 24.8. HRMS (ESI+) *m/z* [M + H^+] calcd for $C_{13}H_{17}O_4$, 237.1126, found 237.1122.

Methyl 4-(hydroxymethyl)-5-isopropyl-2-methoxybenzoate (S12): Palladium on carbon (10%,) was added to a solution of S11 (500 mg, 2.11 mmol) in ethyl acetate (6 mL). The suspension was stirred for 16 h under a hydrogen atmosphere before it was filtered through a pad of celite and eluted with EtOAc (20 mL). The eluent was concentrated to afford S12 (476 mg, 94.2 %) as a colorless amorphous solid. ^1H NMR (400 MHz, $CDCl_3$) δ 7.71 (s, 1H), 7.06 (s, 1H), 4.78 (s, 2H), 3.88 (s, 3H), 3.84 (s, 3H), 3.06 (hept, *J* = 6.9 Hz, 1H), 1.22 (d, *J* = 6.8 Hz, 6H). ^{13}C NMR (100 MHz, $CDCl_3$) δ 167.1, 157.5, 143.7, 137.9, 128.9, 118.9, 111.1, 62.5, 56.3, 52.2, 28.0, 23.9 (2). HRMS (ESI+) *m/z* [M + Na^+] calcd for $C_{13}H_{18}O_4Na$, 261.1103, found 261.1091.

Methyl 4-formyl-5-isopropyl-2-methoxybenzoate (S13): Manganese dioxide (1.64 g, 18.8 mmol, 10.0 eq.) was added to a solution of S12 (450 mg, 1.88 mmo0l, 1.0 eq.) in dichloromethane at rt. The resulting mixture was stirred at rt for 16 h, filtered through a small pad of celite, eluted with ethyl acetate, and concentrated. The residue was purified by flash chromatography (SiO_2, 1:6 ethyl acetate/hexanes) to afford S13 (367.3 mg, 82.7 %) as a yellow colorless solid. ^1H NMR (400 MHz, $CDCl_3$) δ 10.44 (s, 1H), 7.77 (s, 1H), 7.40 (s, 1H), 3.91 (d, *J* = 3.5 Hz, 6H), 3.84 – 3.75 (m, 1H), 1.30 (d, *J* = 6.9 Hz, 6H). ^{13}C NMR (100 MHz, $CDCl_3$) δ 190.9, 166.5, 156.8, 143.2, 136.1, 129.6, 125.6, 112.0, 56.4, 52.5, 27.2, 24.2 (2). HRMS (ESI+) m/z [M + H^+] calcd for $C_{13}H_{17}O_4$, 237.1127 , found 237.1117.

4-Formyl-5-isopropyl-2-methoxybenzoic acid (S14): Trimethyltinhydroxide (3.63 g, 20.1 mmol, 4.0 eq.) was added to solution of S13 (1.12 g, 5.03 mmol, 1.0 eq.) in 1,2 dichloroethane (25 mL).

The resulting mixture was heated at 75 °C for 50 h, cooled to rt, and concentrated. The residue was suspended in ethyl acetate (100 mL), washed with 1 M hydrochloric acid (3 × 60 mL) and saturated sodium chloride solution (100 mL). The solvent was removed to afford S14 (952 mg, 85.1 %) as a colorless amorphous solid. ^{1}H NMR (400 MHz, $CDCl_3$) δ 10.74 (br s, 1H), 10.52 (s, 1H), 8.27 (s, 1H), 7.53 (s, 1H), 4.13 (s, 3H), 3.79 – 4.13 (m, 1H), 1.36 (d, J = 6.8 Hz, 6H). ^{13}C NMR (100 MHz, $CDCl_3$) δ 190.4, 164.9, 156.2, 145.1, 137.5, 132.6, 122.3, 111.5, 57.3, 27.4, 24.3. HRMS (ESI-) m/z [M – H^+] calcd for $C_{12}H_{13}O_4$, 221.0814, found 221.0809.

4-(Isoindoline-2-carbonyl)-2-isopropyl-5-methoxybenzaldehyde (S15): 1-Ethyl-3-(3-dimethylaminopropyl)carbodiimide (1.63 g, 8.54 mmol, 2.0 eq.) was added to a stirred solution of S14 (950 mg, 4.27 mmol, 1 eq.), isoindoline hydrochloride (731 mg, 4.70 mmol, 1.1 eq.), 1-hydroxybenzotriazole (635 mg, 4.70 mmol, 1.1 eq.) N-,N-diisopropylethylamine (3.26 mL, 18.8 mmol, 4.4 eq.) in dichloromethane 42 mL) at 0 °C. The resulting solution was stirred at rt for 14 h before quenching with saturated sodium bicarbonate solution (30 mL). The organic layer was washed with 1 M hydrochloric acid solution (30 mL) and saturated sodium chloride solution (30 mL), dried over anhydrous sodium sulfate, filtered and concentrated. The residue was purified by flash chromatography (SiO_2, 3:10 hexanes/ethylacetate) to afford S15 (1.22 g, 91.8%) as a white amorphous solid. ^{1}H NMR (400 MHz, $CDCl_3$) δ 10.47 (s, 1H), 7.48 – 7.21 (m, 5H), 7.15 (d, J = 7.4 Hz, 1H), 5.01 (s, 2H), 4.58 (s, 2H), 4.02 – 3.76 (m, 4H), 1.32 (d, J = 6.8 Hz, 7H). ^{13}C NMR (100 MHz, $CDCl_3$) δ 190.8, 167.6, 153.7, 144.9, 136.4, 136.4, 134.4, 132.5, 128.0, 127.7, 126.1, 123.3, 122.7, 111.2, 56.1, 53.4, 52.3, 27.3, 24.4 (2). HRMS (ESI+) m/z [M + Na^+] calcd for $C_{20}H_{21}NO_3Na$, 346.1419, found 346.1404.

5-Hydroxy-4-(isoindoline-2-carbonyl)-2-isopropylbenzaldehyde (4): 1 M solution of boron tribromide (1.22 mmol, 1.22 mL, 2 eq.), was added to a solution of S15 (200 mg, 0.61 mmol) in

anhydrous dichloromethane (6.1 ml) at 0 ºC. The resulting mixture was stirred at rt for 14 h before quenching with saturate sodium bicarbonate solution (5 mL). The aqueous layer was extracted with dichloromethane (2 × 10 mL). The combined organic layers were washed with saturated sodium chloride solution (20 mL), dried over sodium sulfate, filtered and concentrated. The residue was purified by flash chromatography (SiO_2, 1:50 acetone/dichloromethane) to afford 4 (121.8 mg, 63.7 %) as a colorless solid. ^{1}H NMR (400 MHz, $CDCl_3$) δ 10.31 (s, 1H), 10.11 (s, 1H), 7.64 (s, 1H), 7.43 (s, 1H), 7.37 – 7.29 (m, 4H), 5.08 (s, 5H), 3.93 (h, J = 6.9 Hz, 1H), 1.34 (d, J = 6.7 Hz, 6H). HRMS (ESI-) m/z [M – H^+] calcd for $C_{19}H_{18}NO_3$, 308.1287, found 308.1282.

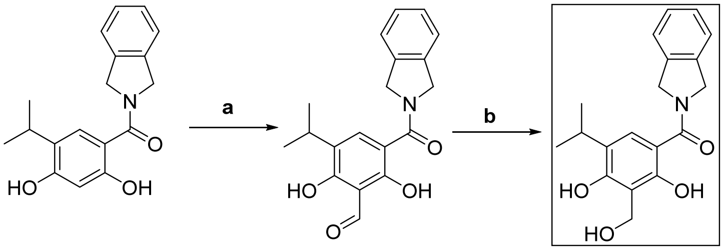

Supplementary Figure 10: Synthetic scheme for compound 5. Detailed synthetic methods and characterization provided below:

2,6-Dihydroxy-3-(isoindoline-2-carbonyl)-5-isopropylbenzaldehyde (S17): S16 was prepared following a literature procedure. A solution of S16 (400 mg, 1.34 mmol, 1 eq.), hexamethylenetetramine (376 mg, 2.68 mmol, 2.0 eq.) in trifluoroacetic acid was heated at 100 ºC for 14 h in a sealed tube, cooled to rt, and solvent was removed. The residue was treated with 3 M hydrochloric acid and the resulting mixture was heated at 60 ºC for 3 h. The mixture was cooled to rt, diluted with water (20 ml), extracted with ethyl acetate (2 × 20 mL). The organic layer was

washed with water (30 ml), saturated sodium bicarbonate solution (30 mL), and saturated sodium chloride solution (30 mL), dried over anhydrous sodium sulfate, filtered, and concentrated. The residue was purified by flash chromatography (SiO_2, 1:9 ethyl acetate/hexanes) to afford S17 (312.2 mg, 71.8 %) as a pale yellow amorphous solid. ^{1}H NMR (500 MHz, $CDCl_3$) δ 13.06 (s, 1H), 12.74 (s, 1H), 10.44 (s, 1H), 7.74 (s, 1H), 7.34 (d, J = 2.2 Hz, 4H), 5.12 (s, 4H), 3.36 – 3.27 (m, 1H), 1.28 (d, J = 6.9 Hz, 6H). ^{13}C NMR (125 MHz, $CDCl_3$) δ 195.1, 170.7, 165.0, 164.0, 135.7, 133.5 (2), 128.2 (2), 126.2, 122.8 (2), 110.1, 106.9, 54.8 (2), 26.1, 22.7 (2). HRMS (ESI+) *m/z* [M + H^+] calcd for $C_{19}H_{20}NO_4$, 338.2120, found 338.2117.

(2,4-Dihydroxy-3-(hydroxymethyl)-5-isopropylphenyl)(isoindolin-2-yl)methanone (5): Sodium borohydride (2.8 mg, 0.06 mmol) was added to a solution of S17 (20 mg, 0.07 mmol, 2.0 eq.) in a solvent mixture of tetrahydrofuran (1.5 mL) and methanol (0.5 mL) at ºC. The resulting mixture was stirred at rt for 1 h before the addition of 1 M hydrochloric acid (2 mL). The aqueous layer was extracted with ethyl acetate (2 × 5 mL), and the combined organic layers washed with saturated sodium chloride solution, dried over anhydrous sodium sulfate, filtered, and concentrated. The residue was purified by preparatory TLC (SiO_2, 1:3 ethyl acetate/hexanes) to give 5 as a colorless amorphous solid (14.2 mg, 72.2 %). ^{1}H NMR (500 MHz, $CDCl_3$) δ 11.72 (s, 1H), 8.76 (s, 1H), 7.43 (s, 1H), 7.32 (s, 4H), 5.11 (s, 6H), 3.34 – 3.24 (m, 1H), 2.31 (s, 1H), 1.28 (d, J = 6.9 Hz, 6H). ^{13}C NMR (125 MHz, $CDCl_3$) δ 171.7, 158.6, 157.1, 136.2 (2), 128.0, 126.3 (2), 125.5, 122.8 (2), 111.4, 108.3, 59.3, 54.2 (2), 26.6, 23.1 (2). HRMS (ESI+) m/z [M + H^+] calcd for $C_{19}H_{22}NO_4$, 328.1549, found 328.1543.

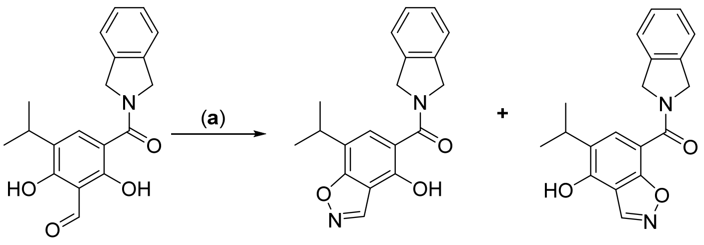

S17 **6a** **S18**

Supplementary Figure 11: Synthetic scheme for compound 6a. Detailed synthetic methods and characterization provided below:

(4-Hydroxy-7-isopropylbenzo[d]isoxazol-5-yl)(isoindolin-2-yl)methanone (6a):[7] Triflic acid (79 μL, 0.90 mmol, 6.0 eq.) and sodium azide (15 mg, 0.23 mmol, 1.5 eq.) were added to a solution of S17 (50 mg, 0.15 mmol, 1.0 eq.) in acetonitrile (1.5 mL) at rt. The resulting mixture was stirred for 5 min and concentrated. The residue was treated with water (2 mL) and ethyl acetate (3 mL). The aqueous layer was extracted with ethyl acetate (2 × 3 mL) and the combined organic layers washed with saturated sodium chloride solution (6 mL), dried over anhydrous sodium sulfate, filtered, and concentrated. The residue was purified using preparatory TLC (SiO_2, 1:3 ethyl acetate/hexanes) to afford 6a (18.3 mg, 37.8 %) and S18 (13.1, 27.1 %). ^{1}H NMR (400 MHz, $CDCl_3$) δ 12.42 (s, 1H), 8.86 (d, *J* = 1.3 Hz, 1H), 7.65 (s, 1H), 7.34 (s, 4H), 5.14 (s, 4H), 3.40 (hept, *J* = 7.0 Hz, 1H), 1.45 (d, *J* = 7.1 Hz, 6H). ^{13}C NMR (100 MHz, $CDCl_3$) δ 171.1, 163.7, 156.1, 145.2, 135.9, 128.2 (2), 126.5 (2), 122.8 (2), 121.6, 112.9, 110.4, 54.2 (2), 29.4, 22.7 (2). HRMS (ESI+) m/z [M + H$^+$] calcd for $C_{19}H_{19}N_2O_3$, 323.1396, found 323.1392.

(4-Hydroxy-5-isopropylbenzo[d]isoxazol-7-yl)(isoindolin-2-yl)methanone (S18): ^{1}H NMR (400 MHz, $CDCl_3$; δ 9.59 (s, 1H), 9.10 (s, 1H), 7.60 (s, 1H), 7.45 – 7.26 (m, 3H), 7.16 (d, *J* = 7.4 Hz, 1H), 5.17 (s, 2H), 4.91 (s, 2H), 3.18 (h, *J* = 7.0 Hz, 1H), 1.06 (d, *J* = 6.9 Hz, 6H). ^{13}C NMR (101 MHz, $CDCl_3$) δ 167.9, 157.9, 151.0, 145.2, 136.5, 135.8, 130.0, 129.4, 128.1, 127.9, 123.1, 122.8,

112.6, 109.5, 53.9, 53.3, 26.4, 22.9 (2). HRMS (ESI+) m/z [M + H$^+$] calcd for $C_{19}H_{19}N_2O_3$, 323.1396, found 323.1399.

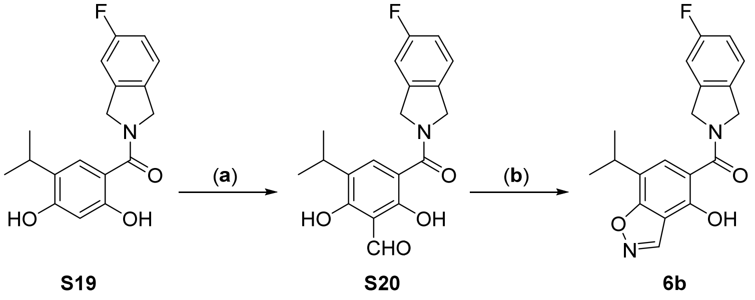

Supplementary Figure 12: Synthetic scheme for compound 6b. Detailed synthetic methods and characterization provided below:

3-(5-fluoroisoindoline-2-carbonyl)-2,6-dihydroxy-5-isopropylbenzaldehyde (S20):

S20 was prepared following a reported procedure.[5-6] A solution of S19 (590 mg, 1.87 mmol, 1 eq.), hexamethylenetetramine (524 mg, 3.74 mmol, 2.0 eq.) in trifluoroacetic acid was heated at 100 ºC for 14 h in a sealed tube, cooled to rt, and solvent was removed. The residue was treated with 3 M hydrochloric acid (12 mL) and the resulting mixture was heated at 60 ºC for 3 h. The mixture was cooled to rt, diluted with water (30 ml), extracted with ethyl acetate (2 × 30 mL). The organic layer was washed with water (45 ml), saturated sodium bicarbonate solution (45 mL), and saturated sodium chloride solution (45 mL), dried over anhydrous sodium sulfate, filtered, and concentrated. The residue was purified by flash chromatography (SiO_2, 1:9 ethyl acetate/hexanes) to afford S17 (410 mg, 63.8 %) as a pale yellow amorphous solid which was used further as obtained. ^{1}H NMR (400 MHz, $CDCl_3$) δ 10.3 (s, 1H), 10.1 (s, 1H), 7.6 (s, 1H), 7.4 (s, 1H), 7.4 – 7.3 (m, 3H), 5.1 (s, 4H), 3.9 (hept, J = 6.8 Hz, 1H), 1.3 (d, J = 6.8 Hz, 6H). HRMS (ESI+) m/z [M + H$^+$] calcd for $C_{19}H_{19}FNO_4$, 344.1298, found 344.1293.

(5-fluoroisoindolin-2-yl)(4-hydroxy-5-isopropylbenzo[d]isoxazol-7-yl)methanone (6b): Triflic acid (131 µL, 1.47 mmol, 6.0 eq.) and sodium azide (24mg, 0.37 mmol, 1.5 eq.) were added to a solution of S19 (80 mg, 0.25 mmol, 1.0 eq.) in acetonitrile (2.5 mL) at rt. The resulting mixture was stirred for 5 min and concentrated. The residue was treated with water (3 mL) and ethyl acetate (5 mL). The aqueous layer was extracted with ethyl acetate (2 × 4 mL) and the combined organic layers washed with saturated sodium chloride solution (8 mL), dried over anhydrous sodium sulfate, filtered, and concentrated. The residue was purified using preparatory TLC (SiO_2, 1:3 ethyl acetate/hexanes) to afford 6b (27.3 mg, 34.5%). ^{1}H NMR (400 MHz, $CDCl_3$) δ 12.29 (s, 1H), 8.85 (s, 1H), 7.61 (s, 1H), 7.29–7.26 (m, 1H), 7.06–7.02 (m, 2H), 5.11 (d, *J* = 9.0 Hz, 4H), 3.38 (h, *J* = 6.9 Hz, 1H), 1.44 (d, *J* = 6.9 Hz, 6H). ^{13}C NMR (100 MHz, $CDCl_3$) δ 170.9, 163.5, 162.8, 155.8, 144.9, 137.6, 131.2, 126.2, 124.0, 121.6, 115.3, 112.7, 110.0, 109.8, 54.3, 53.4, 29.2, 22.5 (2). HRMS (ESI+) m/z [M + H^+] calcd for $C_{19}H_{18}FN_2O_3$, 341.1301 found 341.1304.

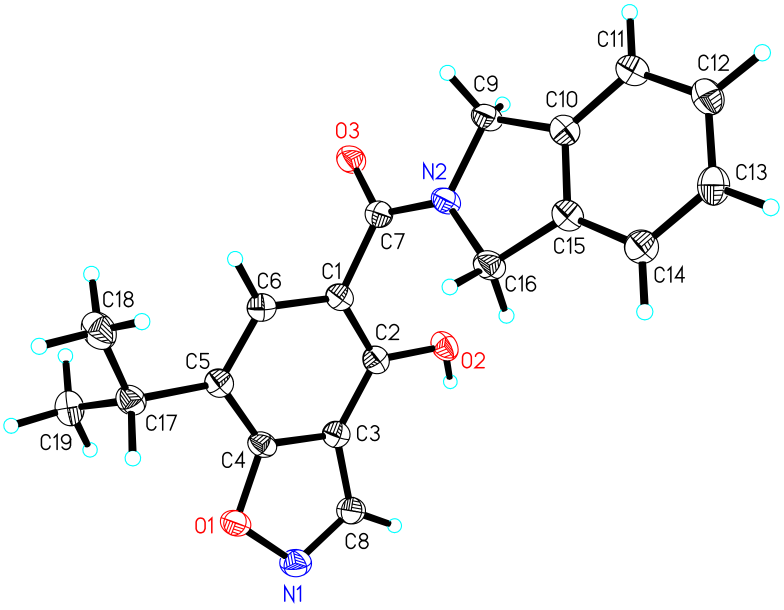

Supplementary Figure 13: X-ray crystal structure of KUNB31 (compound 6a).

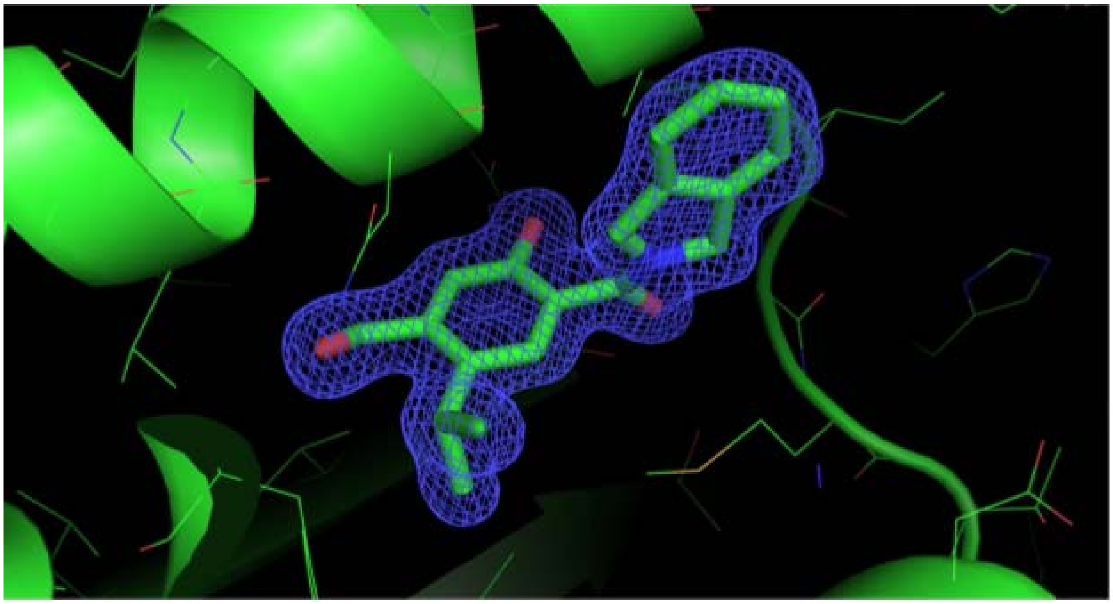

Supplementary Figure 14: Electron density map of compound 2.

Supplementary Table 1: Crystal Data and Structure Refinement for compound 6a.

Empirical formula	$C_{19}H_{18}N_2O_3$
Formula weight	322.35
Temperature	100(2) K
Wavelength	1.54178 Å
Crystal system	Monoclinic
Space group	$P2_1/n$ (an alternate setting of $P2_1/c$ – C_{2h}^5, No. 14)
Unit cell dimensions	a = 9.5496(9) Å α = 90.00°
	b = 9.0349(8) Å β = 98.115(2)°
	c = 18.935(2) Å γ = 90.00°
Volume	1617.3(3) $Å^3$
Z	4
Density (calculated)	1.324 g/cm^3
Absorption coefficient	0.74 mm^{-1}
F(000)	680
Crystal size	0.34 x 0.11 x 0.04 mm^3
Theta range for data collection	4.72° to 67.73°
Index ranges	$-11 \leq h \leq 11$, $-10 \leq k \leq 10$, $-22 \leq l \leq 20$
Reflections collected	10098
Independent reflections	2843 [R_{int} = 0.022]
Completeness to theta = 66.000°	99.2 %
Absorption correction	Multi-scan
Max. and min. transmission	1.000 and 0.883
Refinement method	Full-matrix least-squares on F^2
Data / restraints / parameters	2843 / 0 / 290
Goodness-of-fit on F^2	1.089
Final R indices [I>2σ(I)]	R_1 = 0.033, wR_2 = 0.088
R indices (all data)	R_1 = 0.035, wR_2 = 0.097
Extinction coefficient	0.0025(3)
Largest difference peak and hole	0.22 and -0.22 $e^-/Å^{-3}$

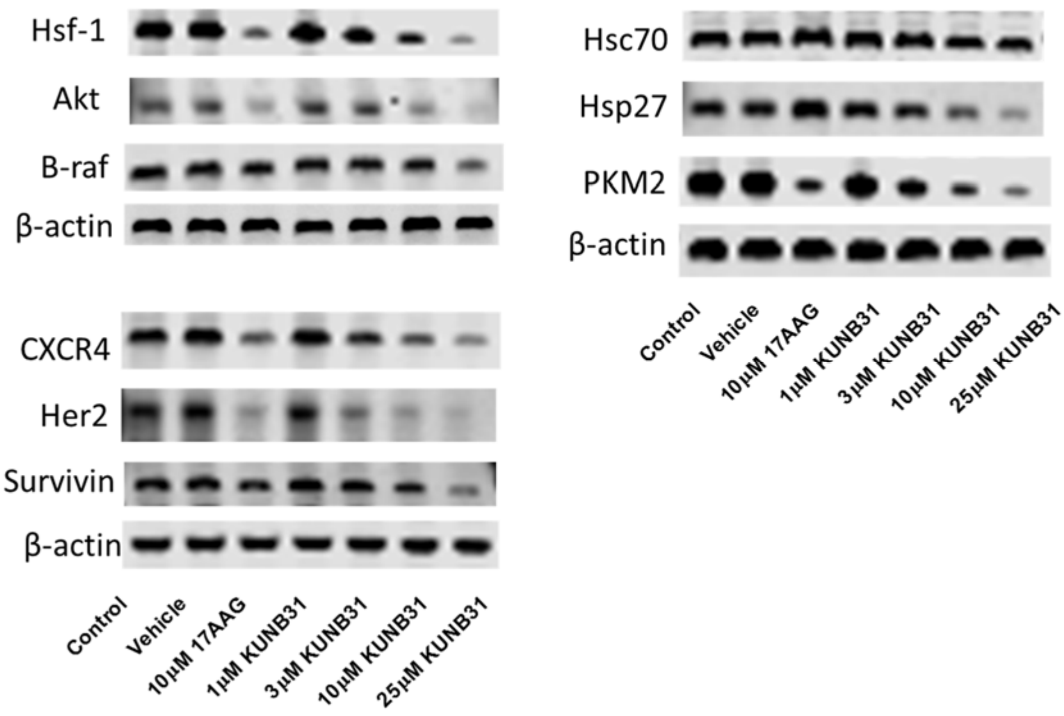

Supplementary Figure 15: Representative western blot analyses 24h after treatment with 6a in UC3 cells, in a dose dependent manor (at concentrations of 1, 3, 5, 15 and 30μM). Negative and positive controls include DMSO (D), and geldanamycin (G) at 500 nM, respectively

Membrane 1

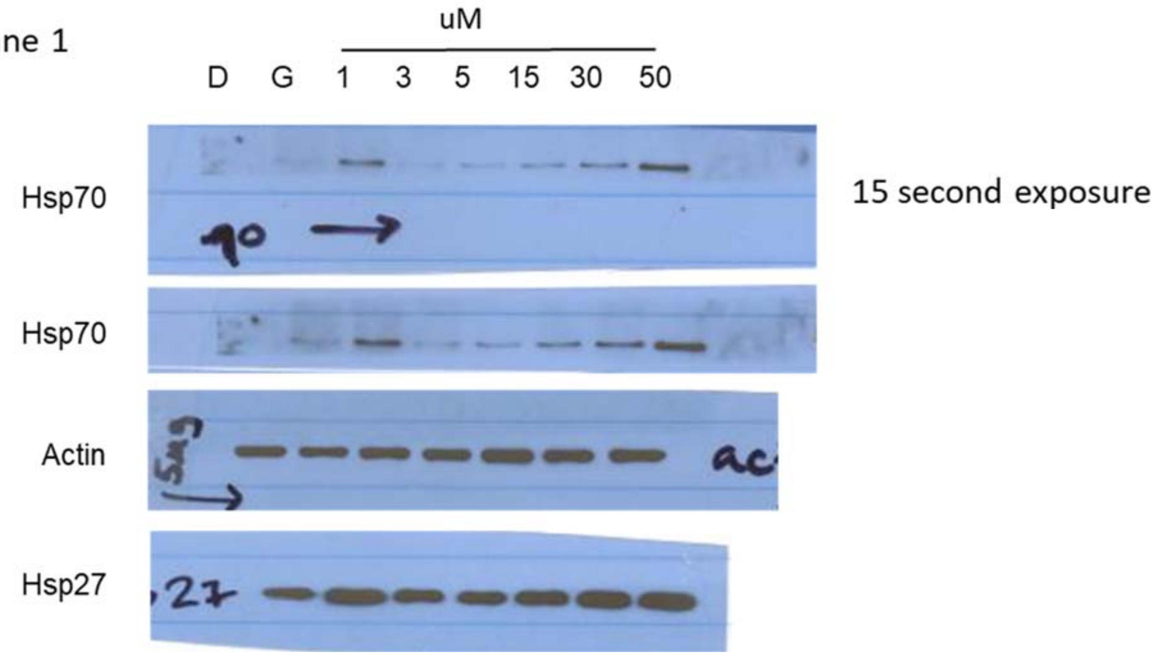

Membrane 2

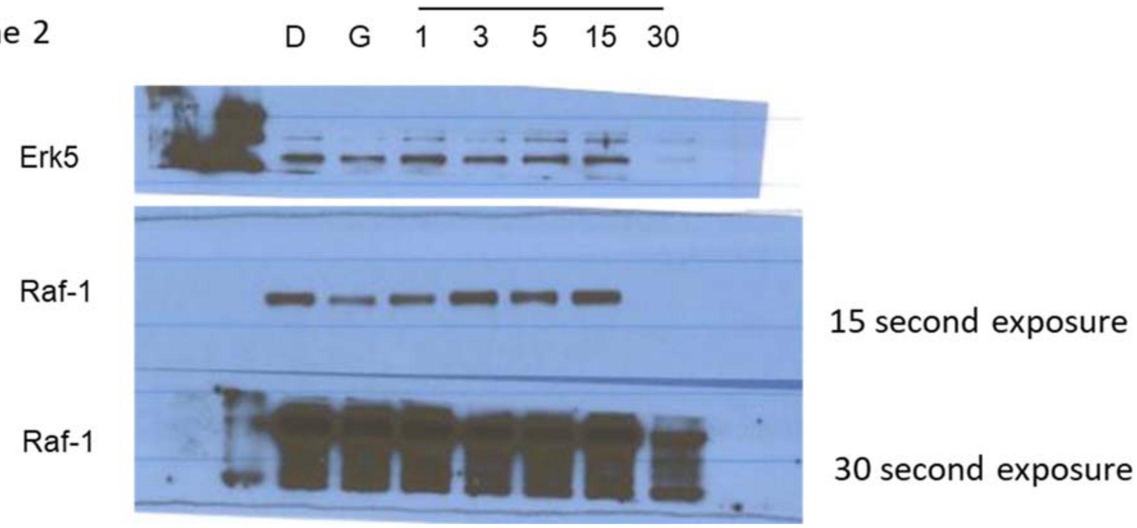

Membrane 3

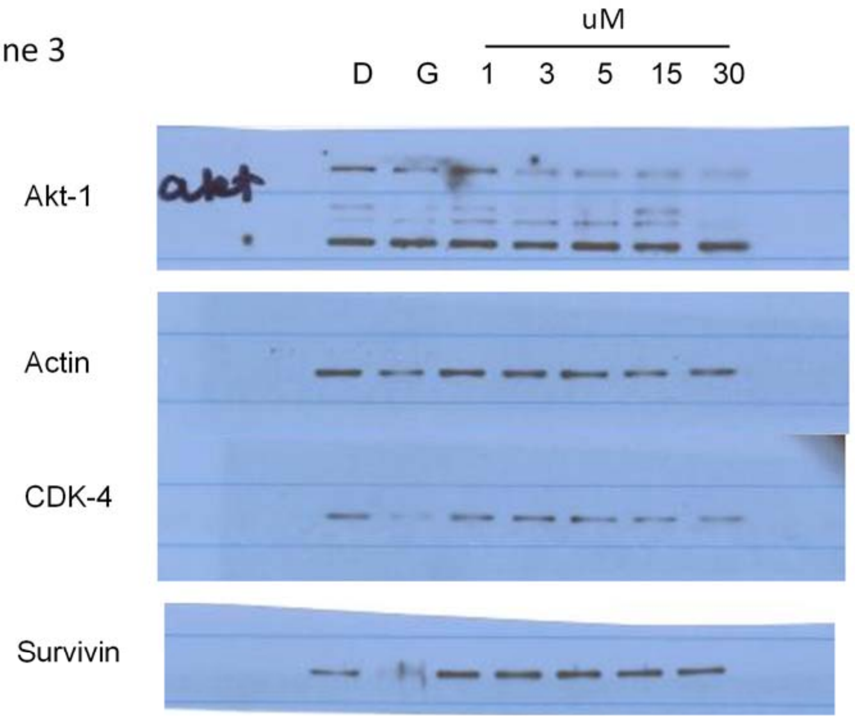

Membrane 4

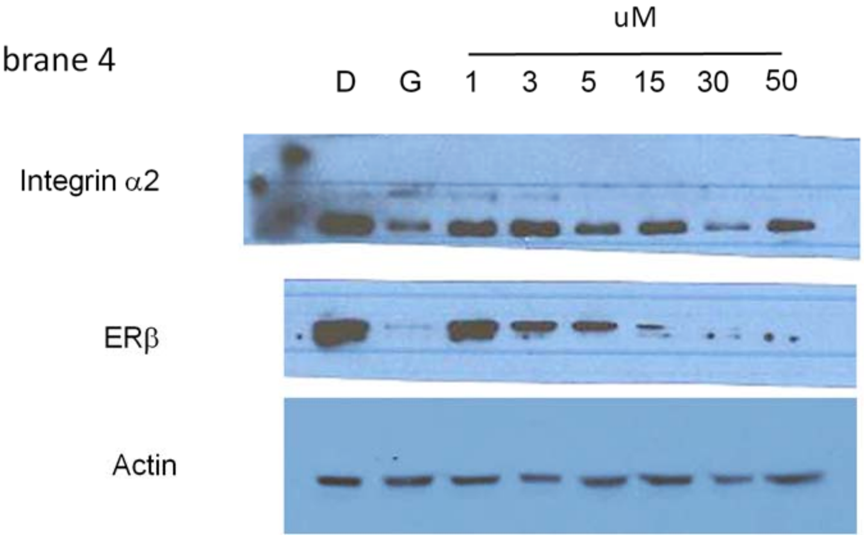

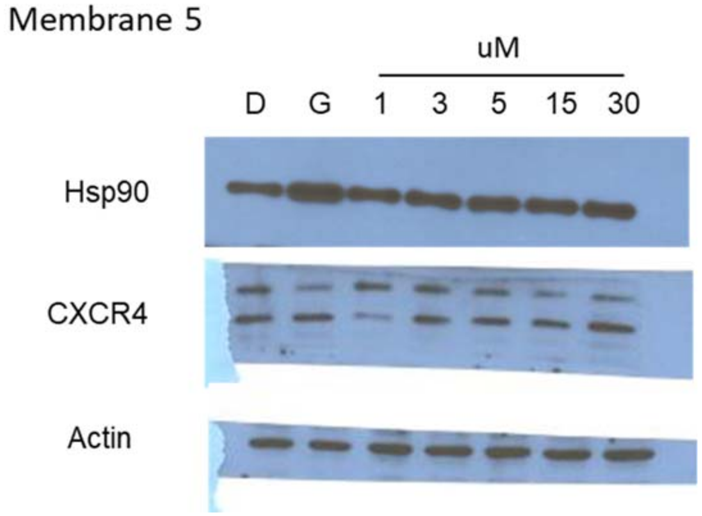

Supplementary Figure 16 (Membranes 1-5): Raw, unprocessed, representative Western blot data for KUNB31 in NCI-H23 cells after 24 h in a dose dependent manor (at concentrations of 1, 3, 5, 15, 30 and 50 μM) as pictured in Figure 5B. Negative and positive controls include DMSO (D), and geldanamycin (G) at 500 nM, respectively.

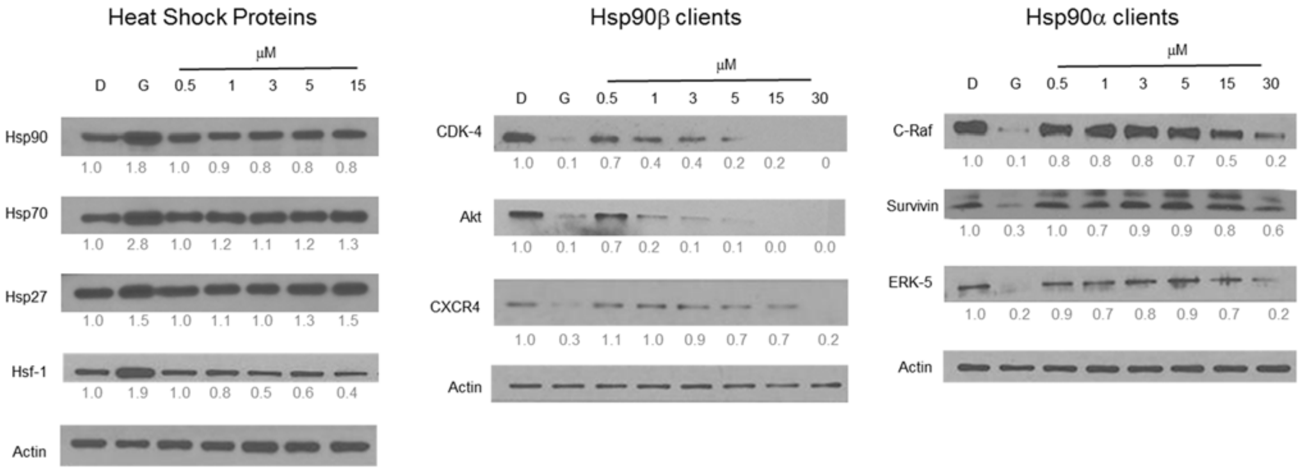

Supplementary Figure 17: Representative western blot analyses 24h after treatment with 6a in HT-29 cells with relative densities, in a dose dependent manor (at concentrations of 1, 3, 5, 15 and 30μM). Negative and positive controls include DMSO (D), and geldanamycin (G) at 500 nM, respectively.

Supplementary Table 2: Crystallographic data and statistics

Data collection	Hsp90βN-KUNB32	Hsp90βN-AK13	Hsp90βN-AK14	Hsp90βN-AK30
Beamline	19-ID, APS	19-ID, APS	19-ID, APS	19-ID, APS
Wavelength, Å	0.97921	0.97922	0.97922	0.97922
Space group	$P6_5$	$P6_5$	$P6_5$	$P6_5$
Cell parameters a, b, c, Å	129.5, 129.5, 106.4	129.3, 129.3, 106.7	128.9, 128.9, 106.7	129.3, 129.3, 107.3
Resolution, Å	50.00–1.70 (1.76–1.70)	50.00–1.90 (1.97–1.90)	50.00–2.40 (2.49–2.40)	50.0–2.5 (2.59–2.50)
Total reflections	635,452	489,542	439,416	195,582
Unique reflections	112,035 (11,154)	79,338 (7,906)	39,226 (3,898)	35,340 (3,532)
Redundancy	5.7 (5.9)	6.2 (5.2)	11.2 (11.4)	5.5 (5.6)
Completeness, %	100.0 (100.0)	99.8 (100.0)	99.8 (99.8)	99.9 (100)
I/σ	23.0 (1.85)	25.6 (1.64)	28.0 (2.37)	15.2 (1.75)
Rsym, %	9.1(67.7)	13.1 (176.5)	17.4 (227.8)	19.3 (185.1)
Refinement statistics				
Resolution range used, Å	42.38–1.69 (1.71–1.69)	29.81–1.90 (1.97–1.90)	30.97–2.40 (2.46–2.40)	32.32–2.50 (2.57–2.50)
No. reflections used	111,987 (3,215)	79,359 (7,529)	39,183 (2,636)	35,199 (2,550)
*R*work/*R*free	17.37/19.66	17.59/22.00	17.01/23.33	17.64/23.92
Rmsd bond lengths, Å	0.008	0.014	0.008	0.008
Rmsd bond angles, °	1.004	1.305	0.996	1.049
Number of atoms (average B, $Å^2$)				
Protein	6,702 (43.7)	6,725 (39.8)	6,721 (57.1)	6,720 (54.2)
Ligand	100 (35.4)	92 (25.7)	96 (39.4)	96 (36.8)
Water	661 (48.8)	517 (43.3)	224 (53.1)	135 (48.1)
Ramachandran values				
Preferred regions, %	96.9	96.6	96.5	95.9
Allowed regions, %	3.1	34	3.5	4.1

Values in parentheses are for the highest-resolution shell. $R_{sym} = \Sigma |I_{obs} - I_{avg}| / \Sigma I_{avg}$; $R_{work} = \Sigma || F_{obs} | - |F_{calc}|| / \Sigma F_{obs}$. R_{free} was calculated using 5% of data.

Supplementary Table 3: Raw FP for compounds 6a against Hsp90α.

	Recorded mP (Hsp90α)								% Polarization to DMSO control		
6a (μM)	6a				DMSO	Tracer		6a (μM)	Hsp90α		
25	188	175	182		335.71	130.33		25	27.92	21.91	25.32
10	222	235	232					10	44.79	51.12	49.66
5	250	252	257					5	58.43	59.08	61.84
2.5	278	274	289					2.5	72.06	70.11	77.42
1	305	310	309					1	85.21	87.64	87.16
0.5	314	326	321					0.5	89.59	95.27	93.00
0.25	319	328	330					0.25	92.02	96.41	97.06
0.1	331	326	329					0.1	97.70	95.43	96.57

Supplementary Table 4: Raw FP for compounds 6a against Hsp90β.

	Recorded mP (Hsp90β)								% Polarization to DMSO control		
6a (μM)	6a				DMSO	Tracer		6a (μM)	Hsp90β		
25	123	132	118		318.29	124.38		25	-0.71	3.76	-3.29
10	127	127	122					10	1.52	1.35	-1.06
5	124	115	135					5	-0.02	-4.84	5.30
2.5	138	120	134					2.5	7.02	-2.09	4.79
1	154	155	149					1	15.10	15.79	12.70
0.5	168	172	167					0.5	22.67	24.73	22.15
0.25	204	198	207					0.25	41.06	37.79	42.44
0.1	241	240	242					0.1	60.31	59.63	60.83

Supplementary Table 5: Raw FP for compounds 5 against Hsp90β.

	Recorded mP (Hsp90β)								% Polarization to DMSO control		
5 (μM)	5				DMSO	Tracer		5 (μM)	Hsp90β		
50	167	167	166		318.29	124.38		50	21.98	21.98	21.29
25	150	165	158					25	13.21	21.12	17.17
10	178	183	185					10	27.82	30.40	31.26
5	218	211	215					5	48.11	44.50	46.91
2.5	245	247	247					2.5	62.38	63.06	63.24
1	268	273	267					1	73.89	76.47	73.55

Supplementary Table 6: Raw FP data for compound 4 against Hsp90α.

	Recorded mP (Hsp90α)							% Polarization to DMSO control		
4 (μM)	4			DMSO	Tracer		4 (μM)	Hsp90α		
10	166	169	166	319.86	120.9		10	22.83	24.34	22.83
5	178	186	181				5	28.53	32.72	30.37
2.5	190	196	192				2.5	34.56	37.91	35.90
1	224	222	231				1	51.99	50.81	55.34
0.5	262	257	254				0.5	70.75	68.57	66.90
0.25	277	269	280				0.25	78.29	74.27	80.13

Supplementary Table 7: Raw FP data for compound 4 against Hsp90β.

	Recorded mP (Hsp90β)							% Polarization to DMSO control		
4 (μM)	4			DMSO	Tracer		4 (μM)	Hsp90β		
10	143	135	136	317.29	118.43		10	12.19	8.33	9.00
5	148	149	146				5	14.87	15.54	13.86
2.5	154	150	154				2.5	18.06	15.88	17.89
1	168	176	167				1	24.76	28.95	24.43
0.5	201	202	199				0.5	41.69	42.19	40.35
0.25	228	234	232				0.25	55.10	58.29	57.11

Supplementary Table 8: Raw FP data for compound 2 against Hsp90β.

	Recorded mP (Hsp90β)							% Polarization to DMSO control		
2 (μM)	2			DMSO	Tracer		2 (μM)	Hsp90β		
10	135	143	146	300.29	127.86		10	4.14	8.59	10.52
5	150	157	168				5	12.84	16.90	23.47
2.5	175	182	182				2.5	27.53	31.21	31.59
1	209	215	214				1	47.25	50.54	49.96
0.5	241	247	249				0.5	65.62	68.90	70.45
0.25	262	269	266				0.25	77.99	82.05	79.92
0.1	284	283	286				0.1	90.36	90.17	91.52

Supplementary Table 9: Raw FP data for compound 2 against Hsp90α.

	Recorded mP (Hsp90α)								% Polarization to DMSO control		
**2 (μM)**	**2**				**DMSO**	**Tracer**		**2 (μM)**	**Hsp90α**		
**10**	174	154	146					**10**	26.46	16.00	12.00
**5**	167	181	181					**5**	22.97	29.94	29.94
**2.5**	208	214	217		314.71	123.38		**2.5**	44.40	47.19	49.10
**1**	250	255	252					**1**	66.18	68.79	67.22
**0.5**	274	273	268					**0.5**	78.89	78.37	75.58
**0.25**	295	293	285					**0.25**	89.52	88.65	84.64
**0.1**	306	311	302					**0.1**	95.62	98.23	93.53

Supplementary Table 10: Raw FP data for compound 1 against Hsp90α.

	Recorded mP (Hsp90α)								% Polarization to DMSO control		
**1 (μM)**	**1**				**DMSO**	**Tracer**		**1 (μM)**	**Hsp90α**		
**100**	140	137	140					**100**	9.47	8.26	9.47
**50**	144	145	138					**50**	11.45	12.05	8.56
**25**	155	153	160					**25**	16.45	15.39	18.58
**10**	169	158	174		338.52	118.86		**10**	22.83	17.67	25.25
**5**	189	193	202					**5**	31.78	33.90	37.85
**2.5**	233	267	237					**2.5**	51.96	67.59	53.78
**1**	274	277	276					**1**	70.63	71.84	71.69
**0.5**	305	302	299					**0.5**	84.59	83.37	82.16

Supplementary Table 11: Raw FP data for compound 1 against Hsp90β.

	Recorded mP (Hsp90β)								% Polarization to DMSO control		
**1 (μM)**	**1**				**DMSO**	**Tracer**		**1 (μM)**	**Hsp90β**		
**100**	140	137	140					**100**	8.22	4.90	9.38
**50**	144	145	138					**50**	5.73	5.90	4.40
**25**	155	153	160					**25**	12.53	4.40	5.73
**10**	169	158	174		318.57	117.48		**10**	15.34	18.00	10.37
**5**	189	193	202					**5**	23.80	23.30	19.99
**2.5**	233	267	237					**2.5**	32.75	31.92	33.25
**1**	274	277	276					**1**	60.93	62.75	64.41
**0.5**	305	302	299					**0.5**	68.22	73.53	68.55

Supplementary Table 12: Raw FP data for compound 6b against Hsp90α.

	Recorded mP (Hsp90α)								% Polarization to DMSO control		
6b (μM)	6b				DMSO	Tracer		6b (μM)	Hsp90α		
75	169	178	170		331.33	128.24		75	20.07	24.50	20.73
25	213	213	214					25	41.57	41.90	42.23
10	264	238	258					10	66.68	53.88	63.89
3	307	295	277					3	87.85	82.27	73.25
1	328	325	324					1	98.19	97.05	96.39
0.5	326	319	328					0.5	97.54	93.76	98.36
0.25	337	333	329					0.25	102.79	100.66	99.02
0.125	335	329	327					0.125	101.64	98.69	98.03

Supplementary Table 13: Raw FP data for compound 6b against Hsp90β.

	Recorded mP (Hsp90β)								% Polarization to DMSO control		
6b (μM)	6b				DMSO	Tracer		6b (μM)	Hsp90β		
3	212	214	218		326.52	131.62		3	10.97	0.88	16.96
1	244	256	247					1	27.05	23.28	31.32
0.5	248	254	251					0.5	44.66	45.17	44.66
0.25	277	277	277					0.25	74.08	66.38	64.33
0.125	269	285	268					0.125	80.92	79.55	79.72

Supplementary Table 14: Raw FP data for Grp-94-selective inhibitor BnIm against Hsp90β.

	Recorded mP (Hsp90β)								% Polarization to DMSO control		
BnIm (μM)	BnIM				DMSO	Tracer		BnIM (μM)	Hsp90β		
25	193	163	197		335.71	130.33		25	35.56	19.74	37.28
10	227	234	236					10	52.75	56.70	57.74
5	259	263	256					5	69.43	71.32	68.05
2.5	279	290	283					2.5	79.91	85.41	81.80
1	295	310	298					1	87.82	95.56	89.37
0.5	303	306	305					0.5	92.12	93.66	92.98
0.25	303	313	309					0.25	92.12	97.45	95.04
0.1	315	313	317					0.1	98.31	97.10	99.17

Supplementary Table 15: Raw FP data for Grp-94-selective inhibitor BnIm against Hsp90α.

	Recorded mP (Grp94)								% Polarization to DMSO control		
BnIm (µM)	BnIM				DMSO	Tracer		BnIM (µM)	Grp94		
25	133	132	138					25	1.97	1.45	4.59
10	149	148	145					10	10.36	9.84	7.92
5	166	165	173					5	19.28	18.75	22.60
2.5	190	188	183		335.71	130.33		2.5	31.51	30.64	27.84
1	228	236	223					1	51.44	55.63	49.16
0.5	249	216	316					0.5	62.45	45.49	97.75
0.25	262	260	254					0.25	69.61	68.56	65.24
0.1	283	288	275					0.1	80.27	83.07	76.25

Supplementary Table 16: Raw FP data for pan-Hsp90 inhibitor geldanamycin (GDA) against Hsp90α.

	Recorded mP (Hsp90α)								% Polarization to DMSO control		
GDA (µM)	GDA				DMSO	Tracer		GDA (µM)	Hsp90α		
1	133	150	155					1	1.08	9.82	12.39
0.5	152	153	155					0.5	10.84	11.02	12.04
0.25	163	154	164					0.25	16.16	11.87	16.67
0.1	176	170	181		335.71	130.33		0.1	23.18	19.93	25.58
0.05	207	209	210					0.05	38.95	40.15	40.32
0.025	267	267	282					0.025	69.62	69.62	77.33
0.01	310	313	310					0.01	91.73	93.44	92.07
0.005	326	329	328					0.005	100.12	101.49	101.32

Supplementary Table 16: Raw FP data for pan-Hsp90 inhibitor 17-allyl-amino-geldanamycin (17-AAG) against Hsp90α.

	Recorded mP (Hsp90α)								% Polarization to DMSO control		
17AAG (µM)	17-AAG				DMSO	Tracer		17AAG (µM)	Hsp90α		
1	161	162	160					1	15.30	15.99	14.96
0.5	180	181	172					0.5	24.90	25.58	20.78
0.25	178	196	190					0.25	24.04	33.46	30.21
0.1	215	213	212		335.71	130.33		0.1	43.23	42.20	41.69
0.05	251	246	250					0.05	61.57	58.82	61.22
0.025	294	303	288					0.025	83.84	88.47	80.42
0.01	315	312	312					0.01	94.30	92.75	92.75
0.005	328	327	322					0.005	101.15	100.64	97.89

Supplemental Figure 18: H[1] NMR spectra of compound S1.

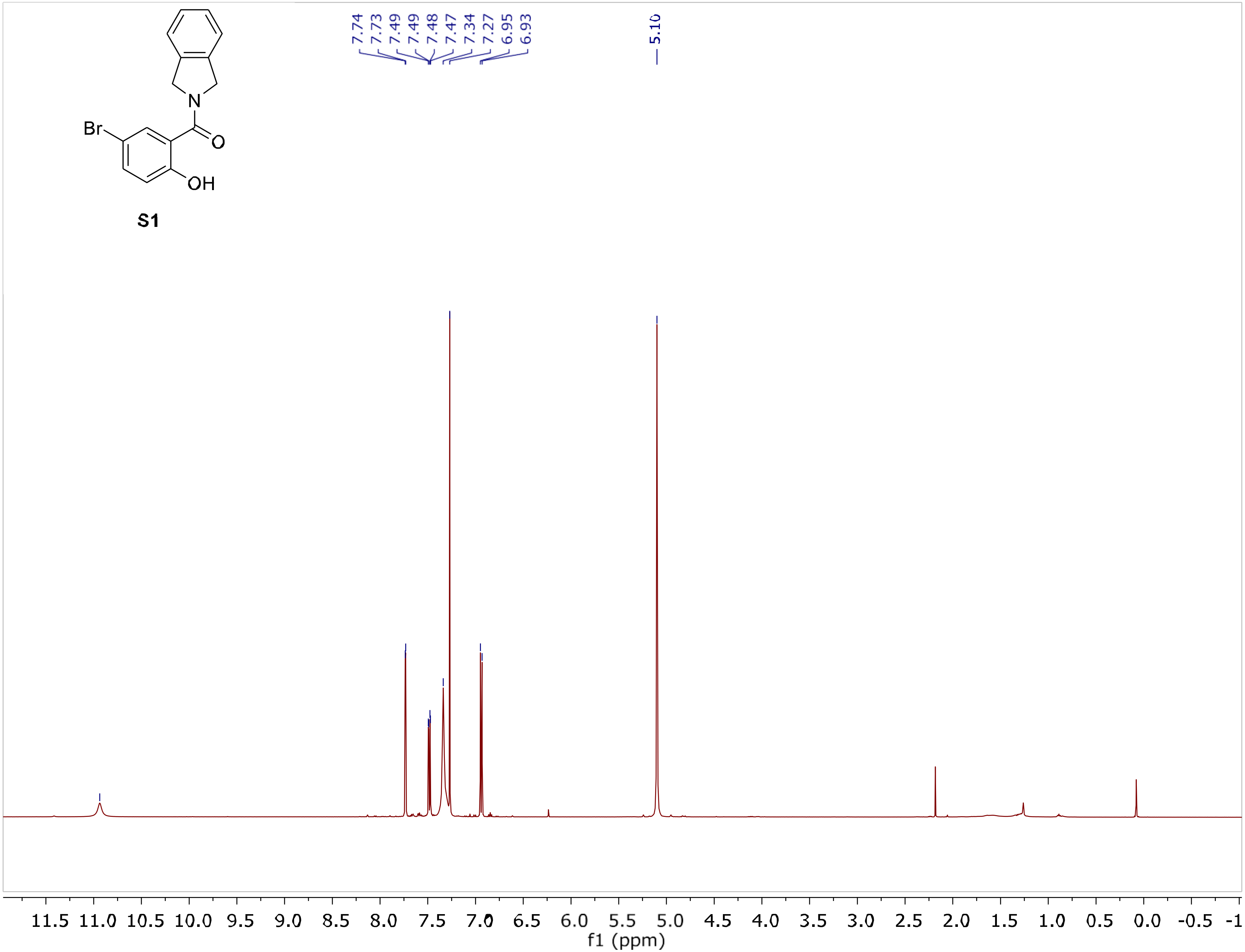

Supplemental Figure 19: C^{13} NMR spectra of compound S1.

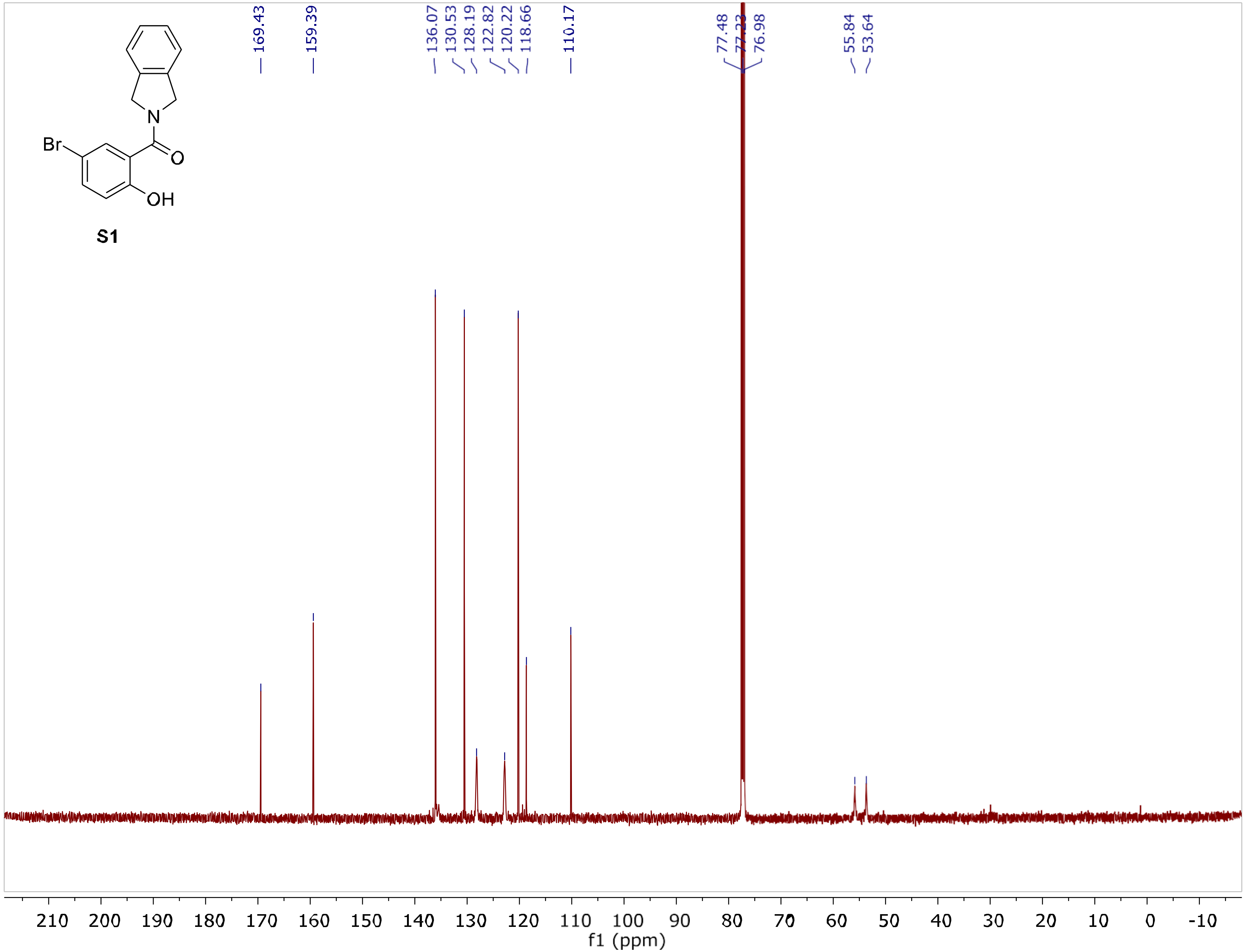

Supplemental Figure 20: H[1] NMR spectra of compound 1.

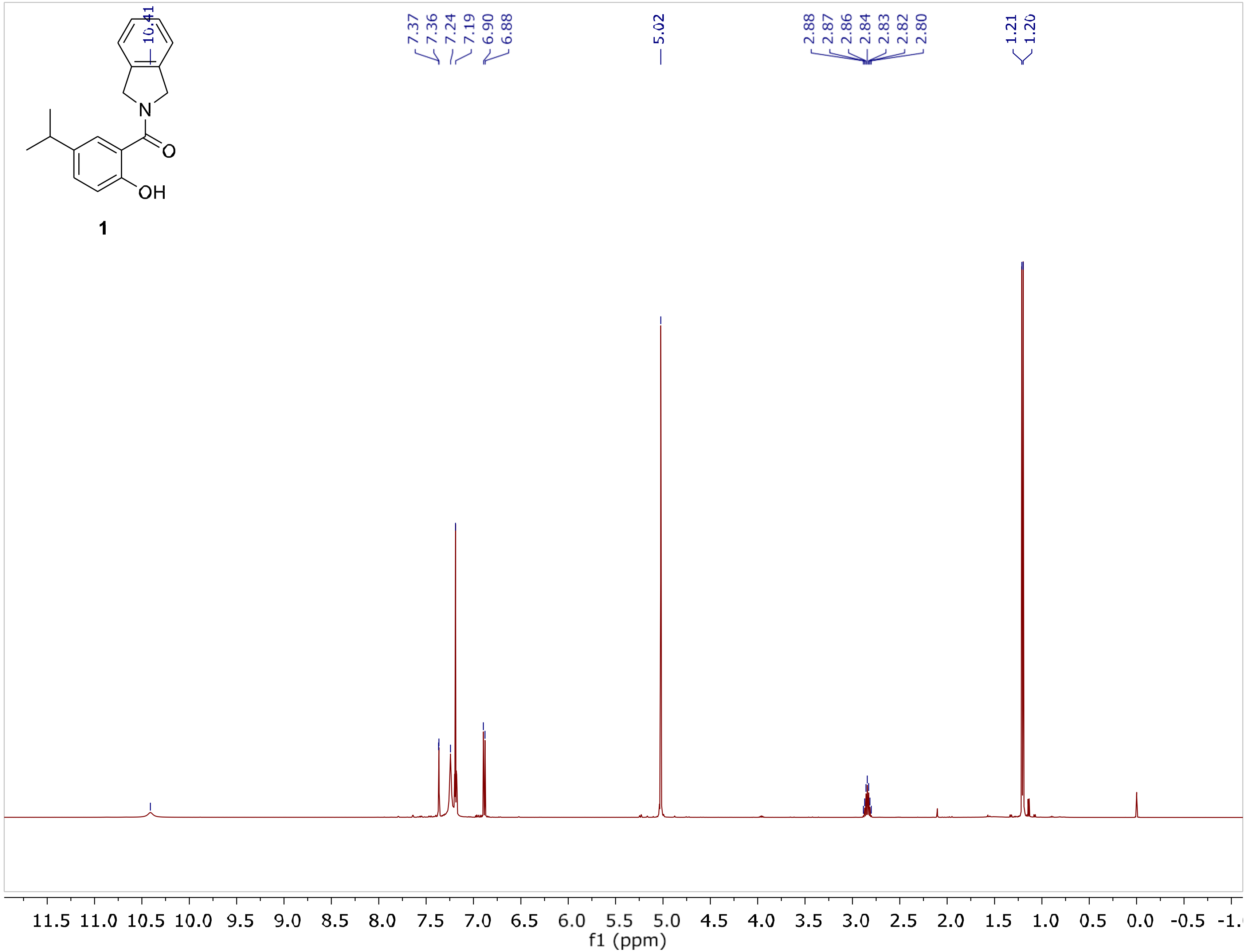

Supplemental Figure 21: C^{13} NMR spectra of compound 1.

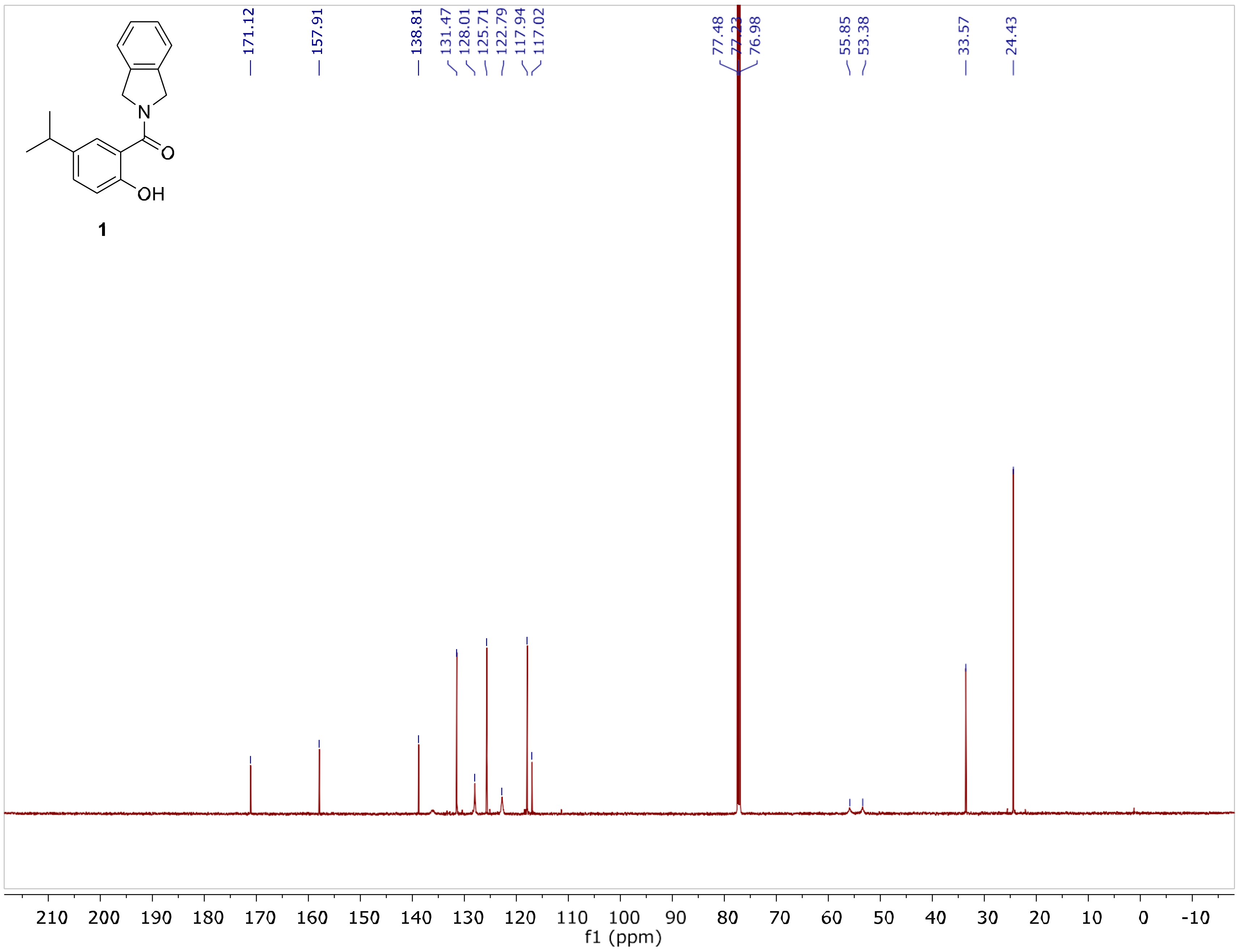

Supplemental Figure 22: H[1] NMR spectra of compound S2.

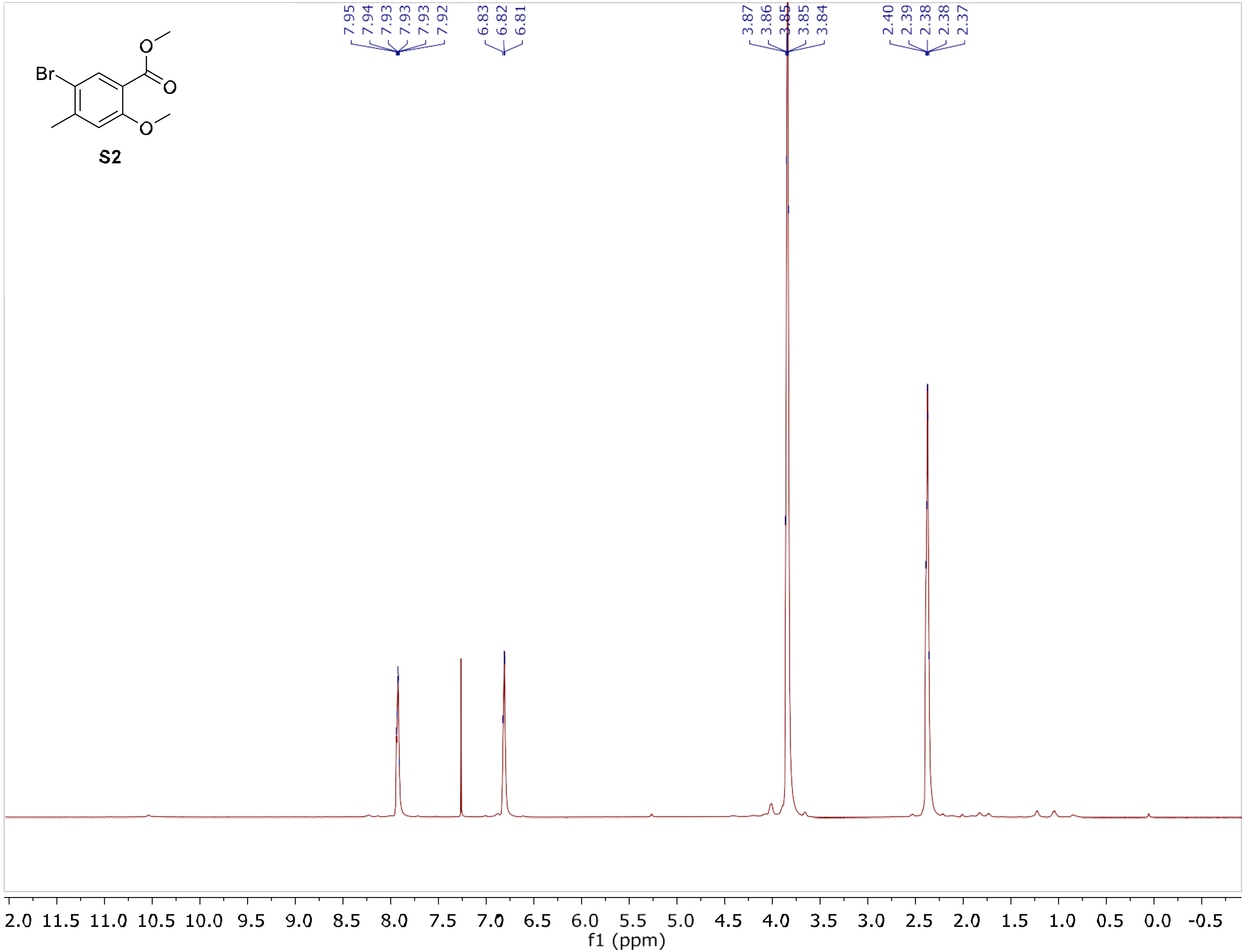

Supplemental Figure 23: C^{13} NMR spectra of compound S1.

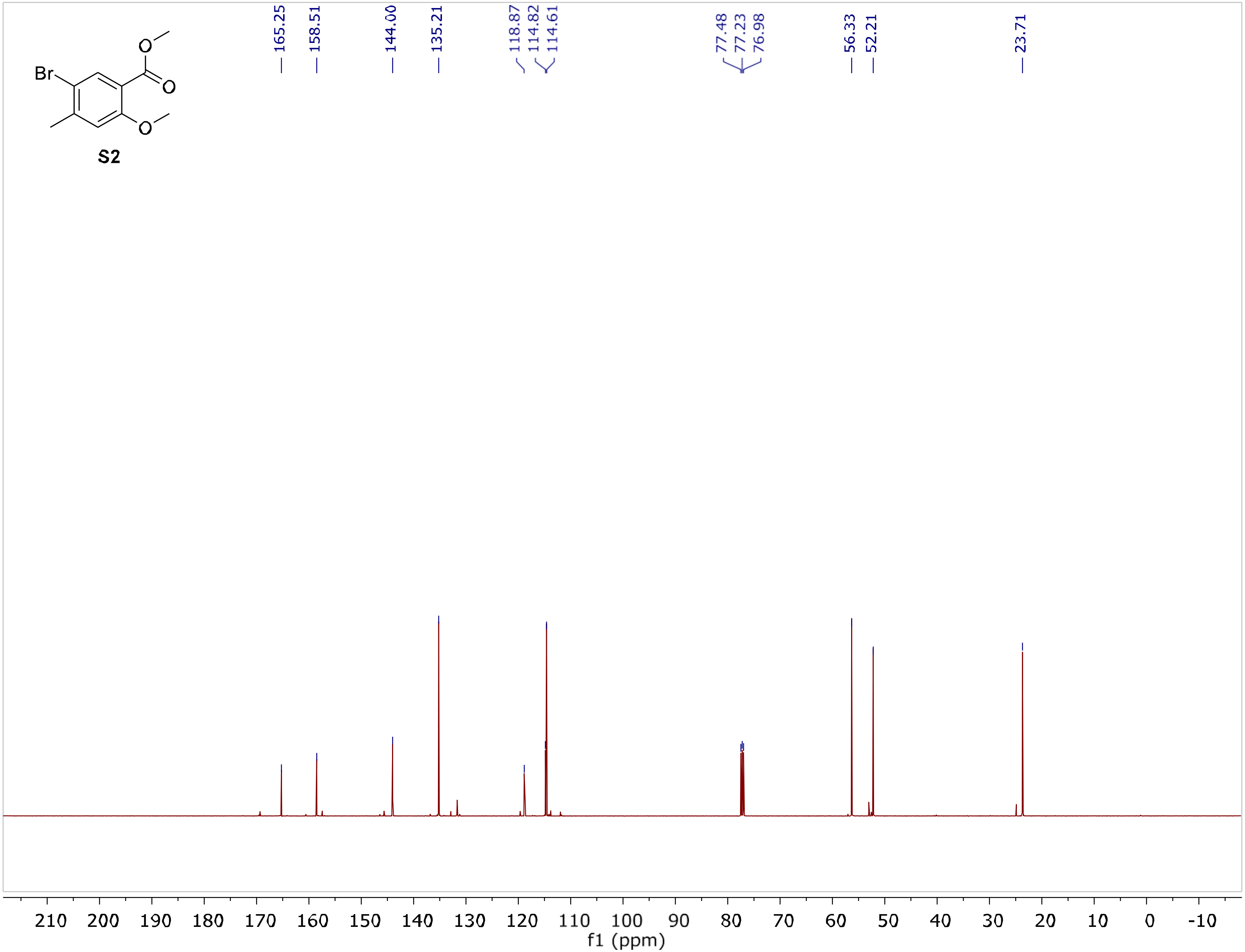

Supplemental Figure 24: H[1] NMR spectra of compound S3.

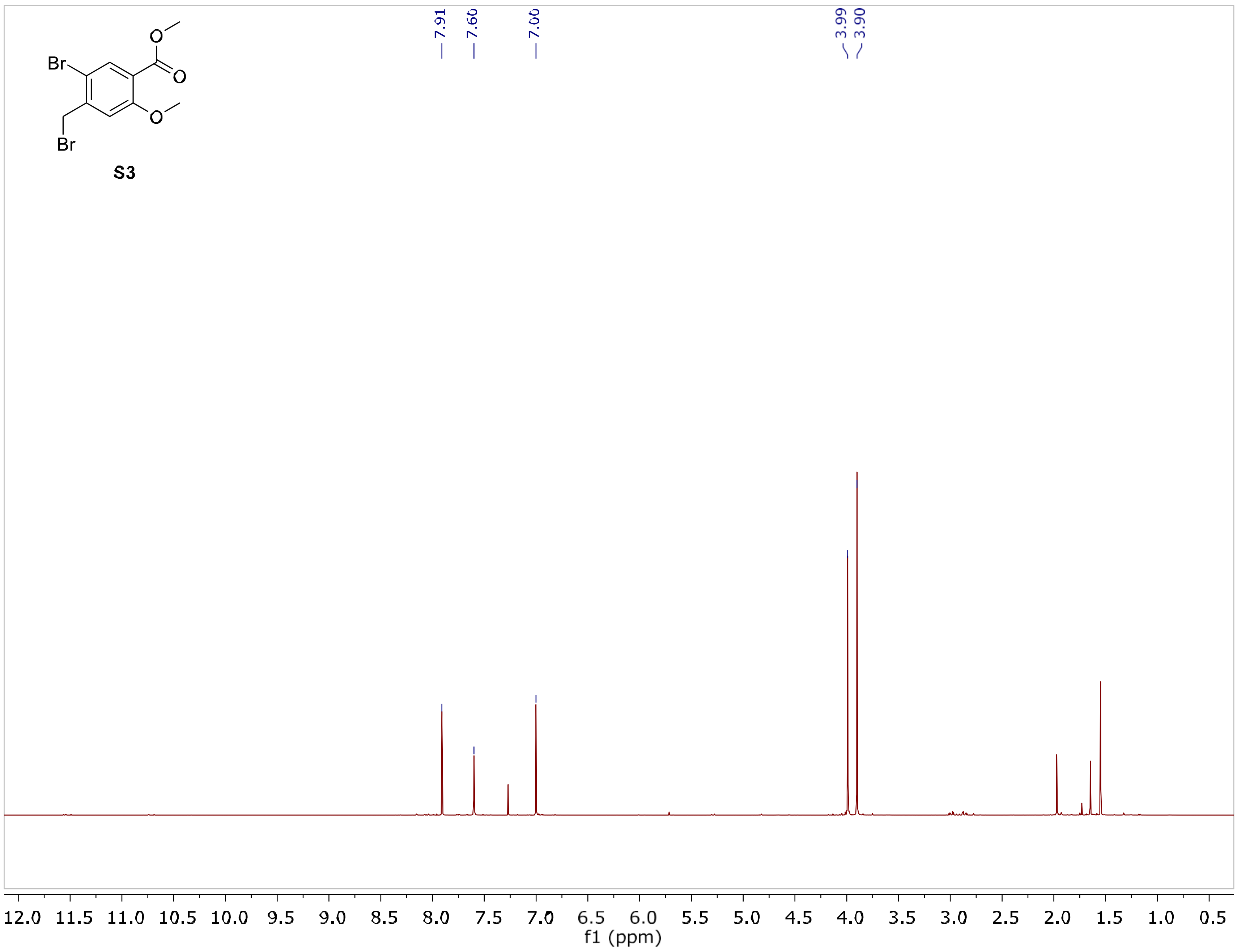

Supplemental Figure 25: C^{13} NMR spectra of compound S3.

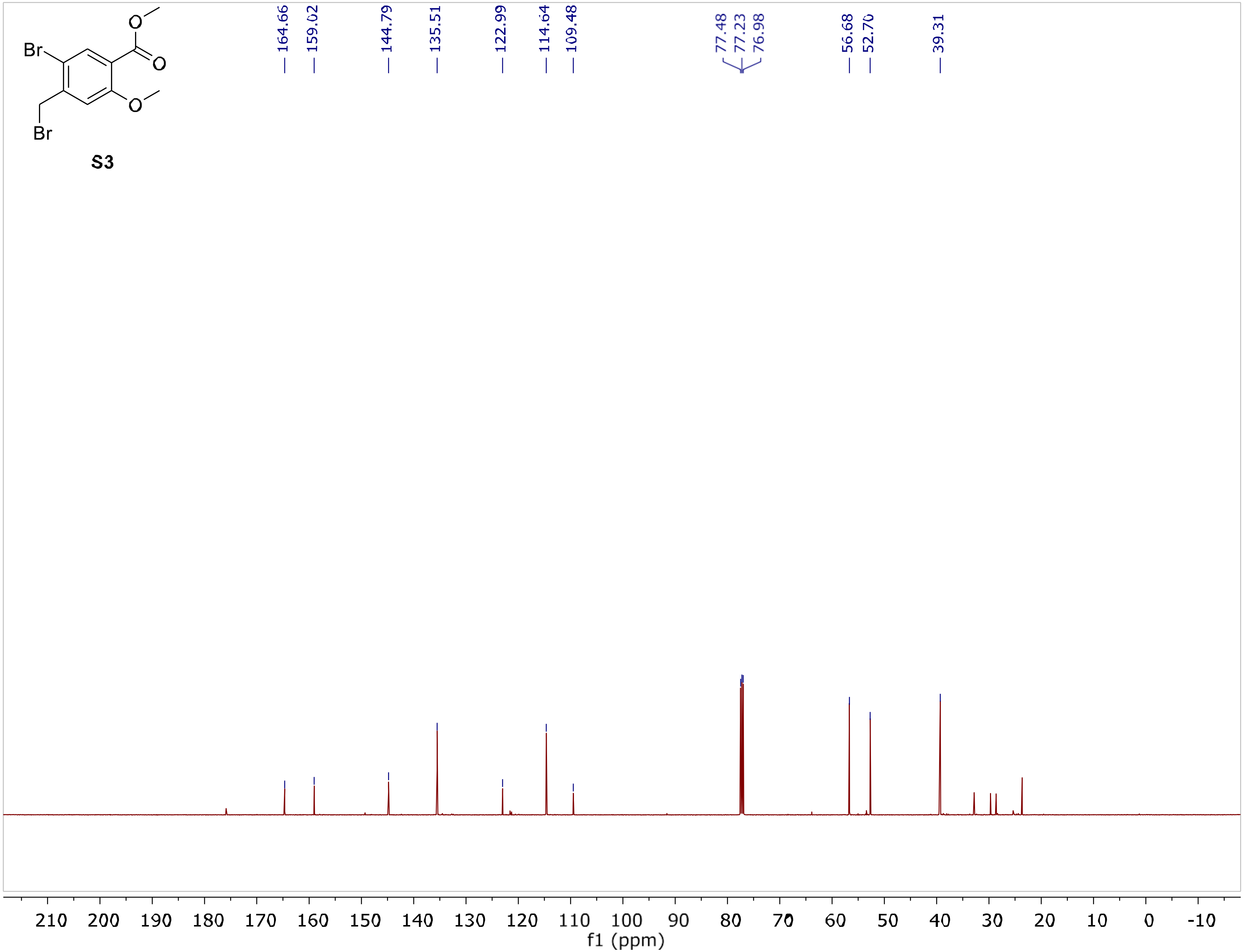

Supplemental Figure 26: H[1] NMR spectra of compound S4.

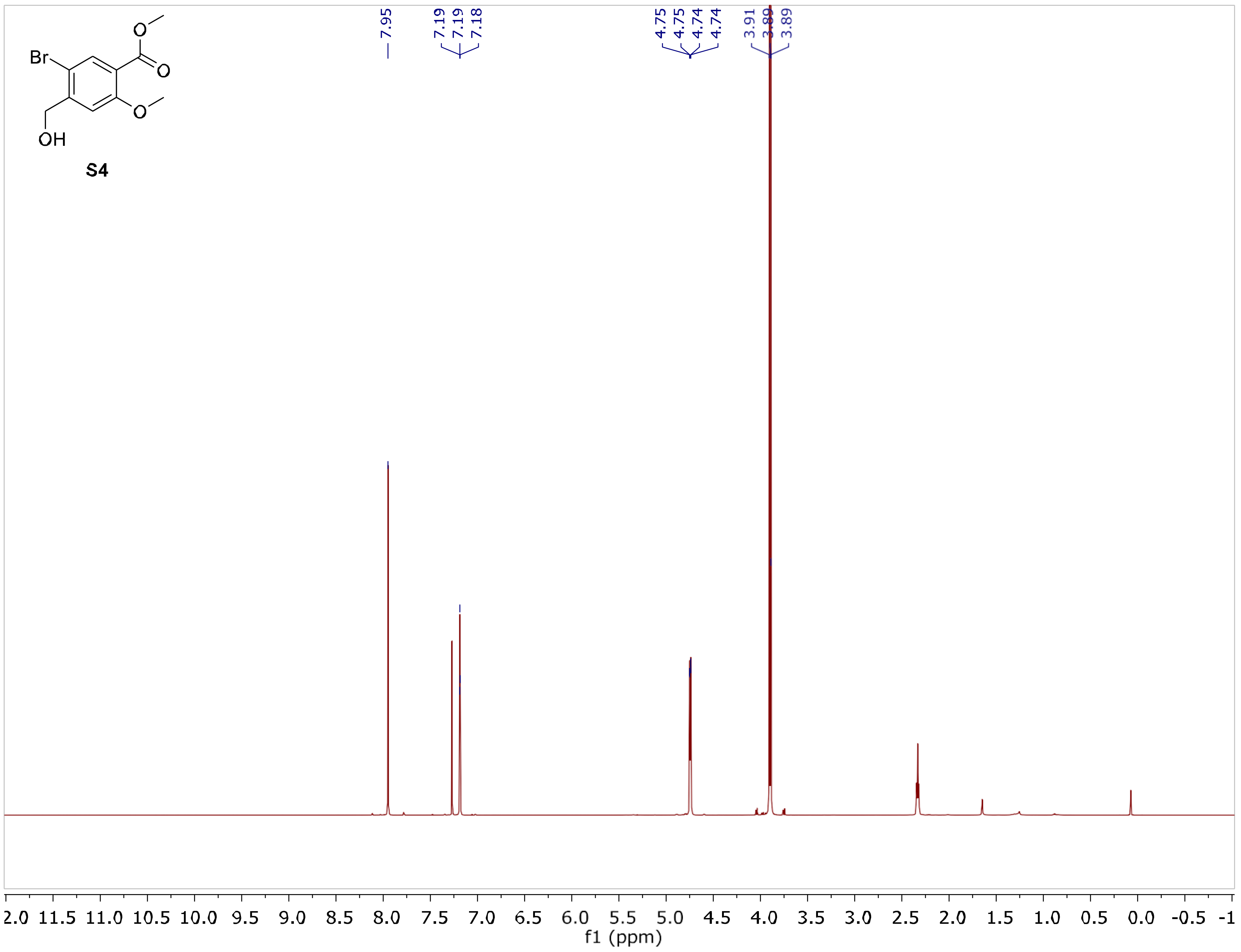

Supplemental Figure 27: C^{13} NMR spectra of compound S4.

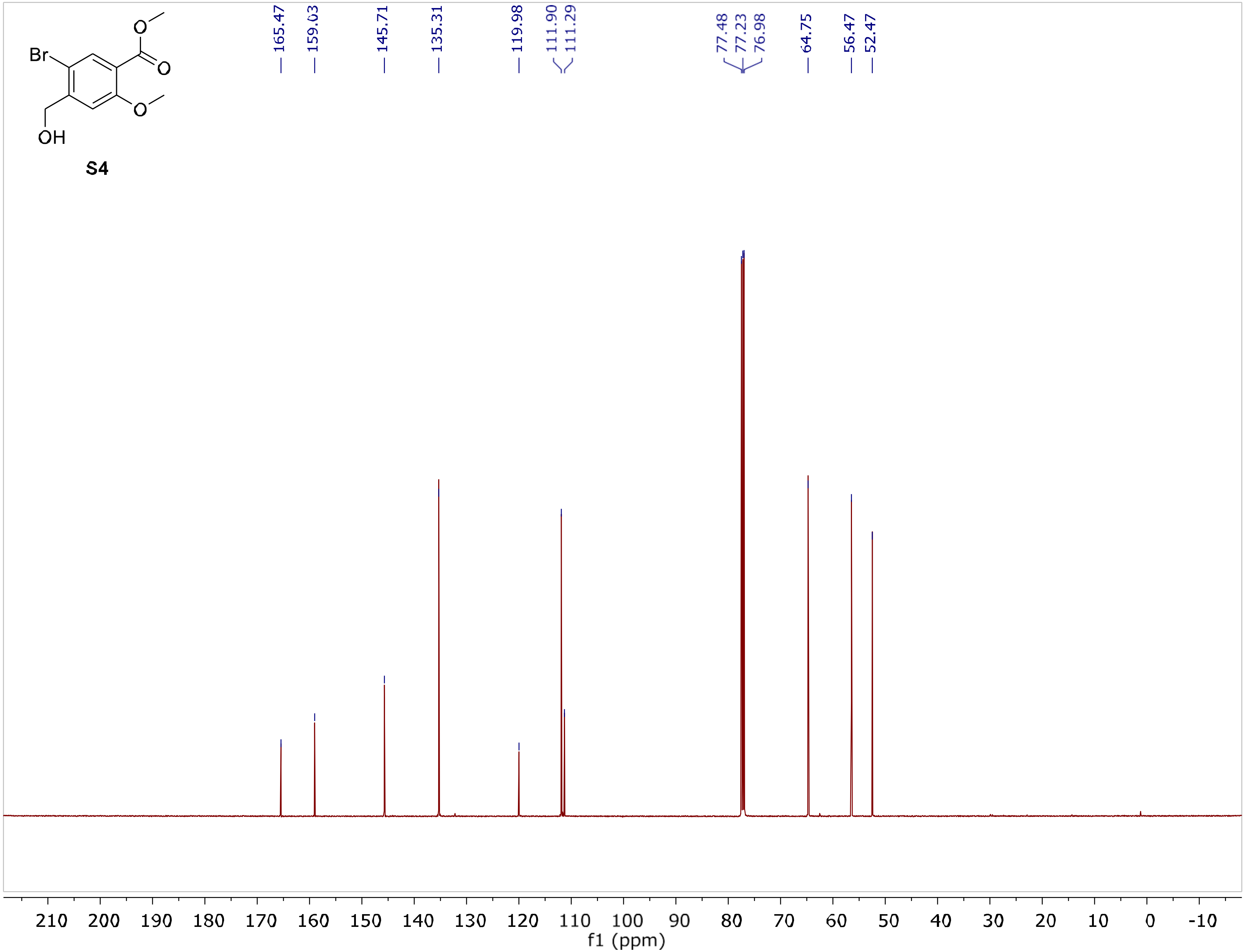

Supplemental Figure 28: H^1 NMR spectra of compound S5.

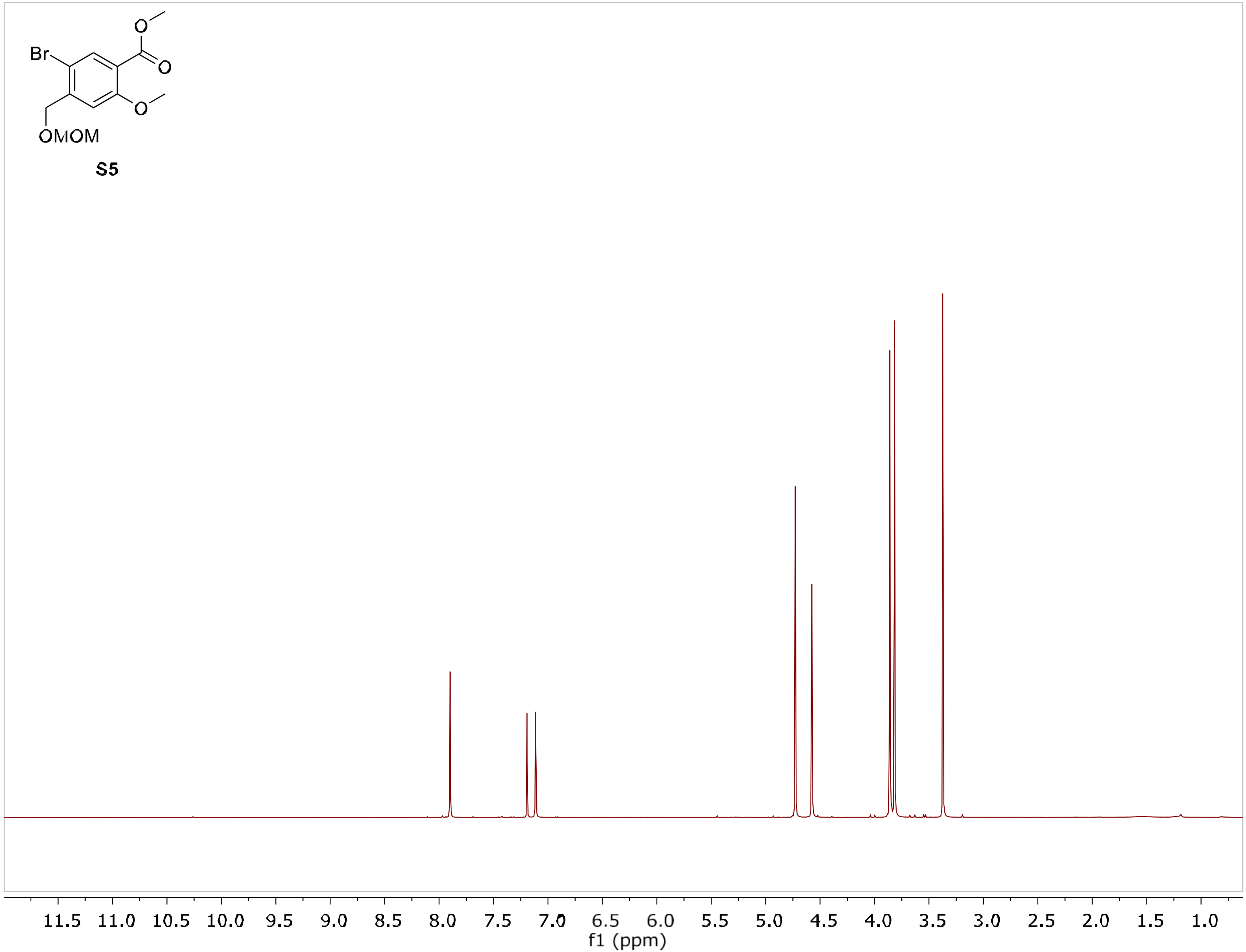

Supplemental Figure 29: C^{13} NMR spectra of compound S5.

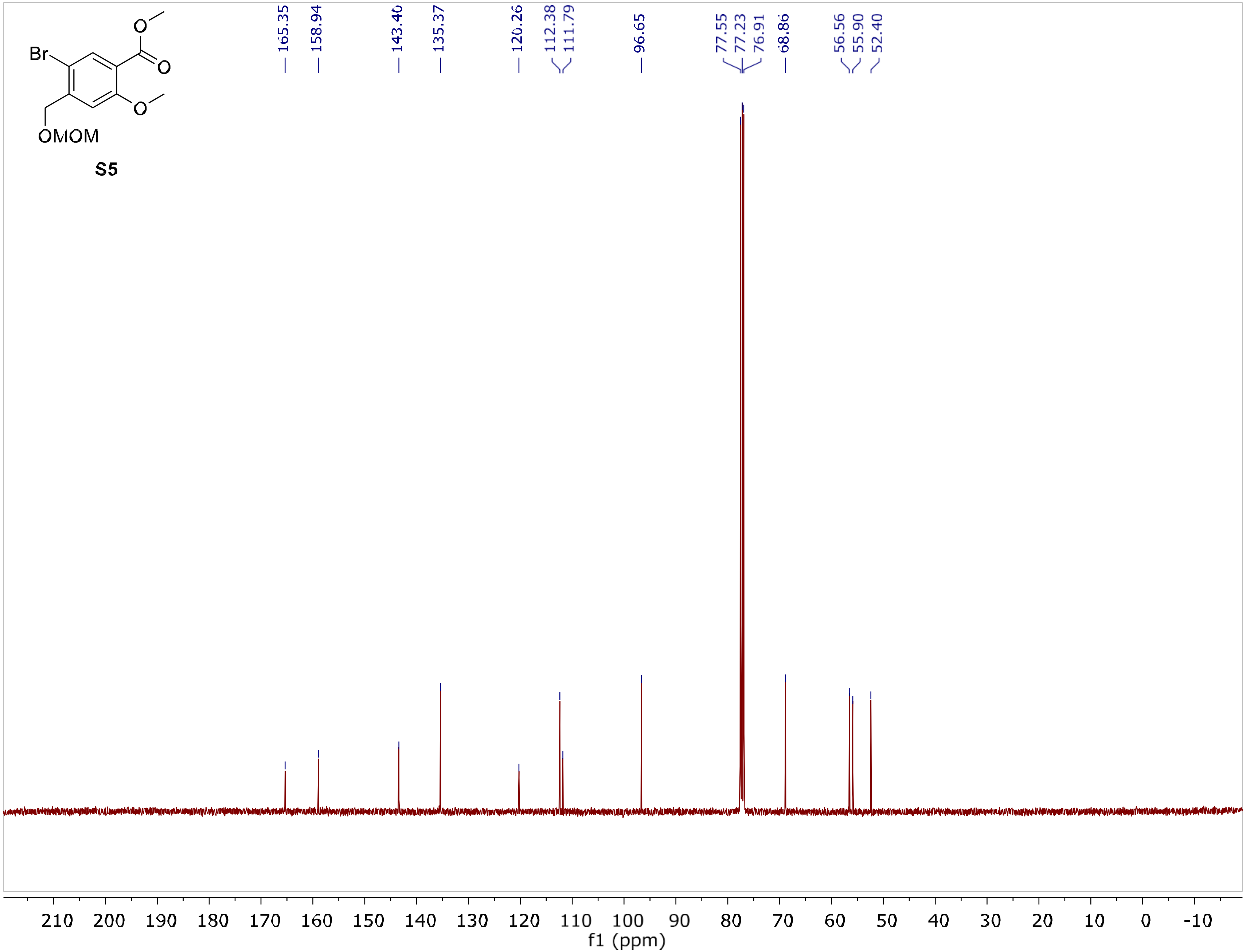

Supplemental Figure 30: H^1 NMR spectra of compound S6.

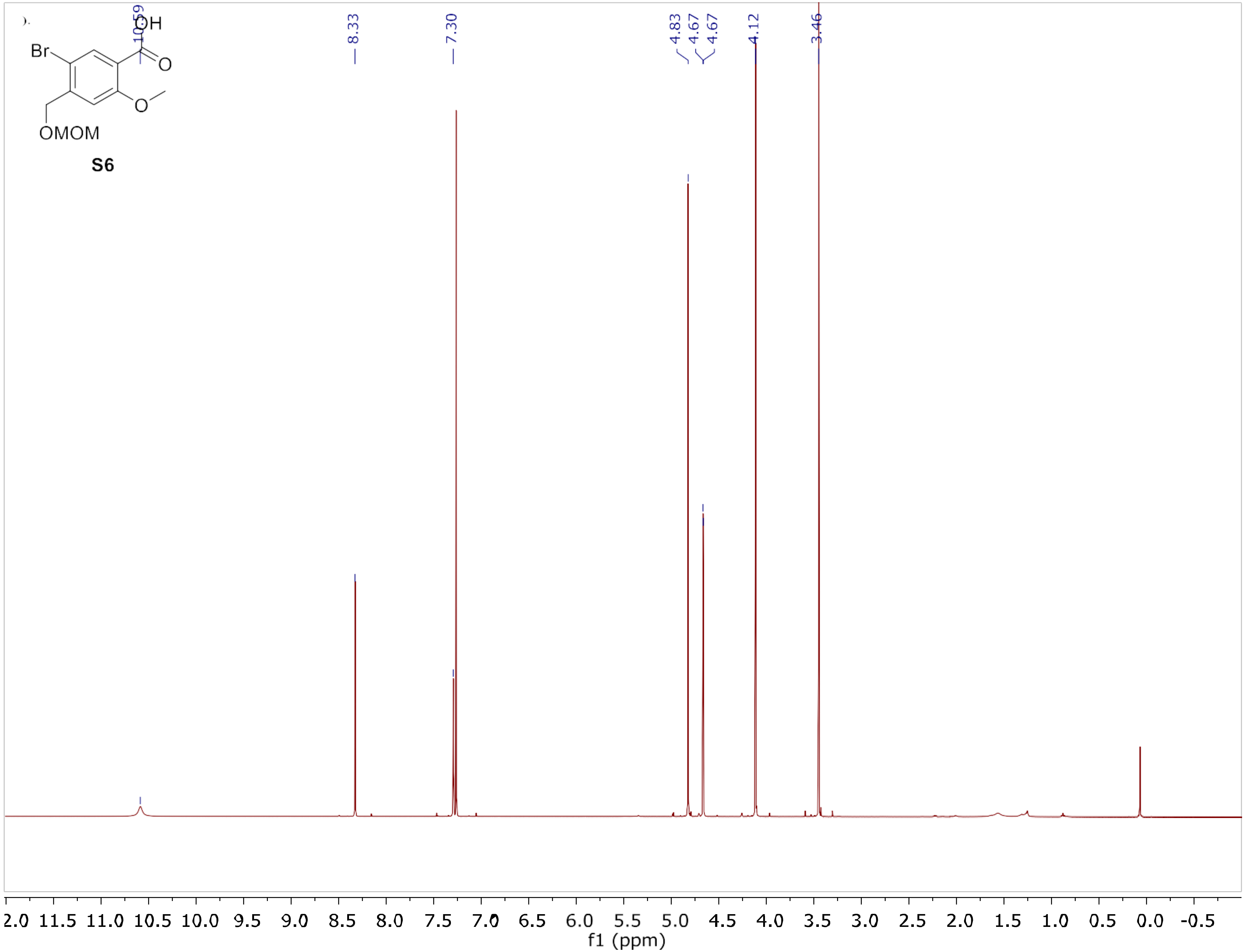

Supplemental Figure 31: C^{13} NMR spectra of compound S6.

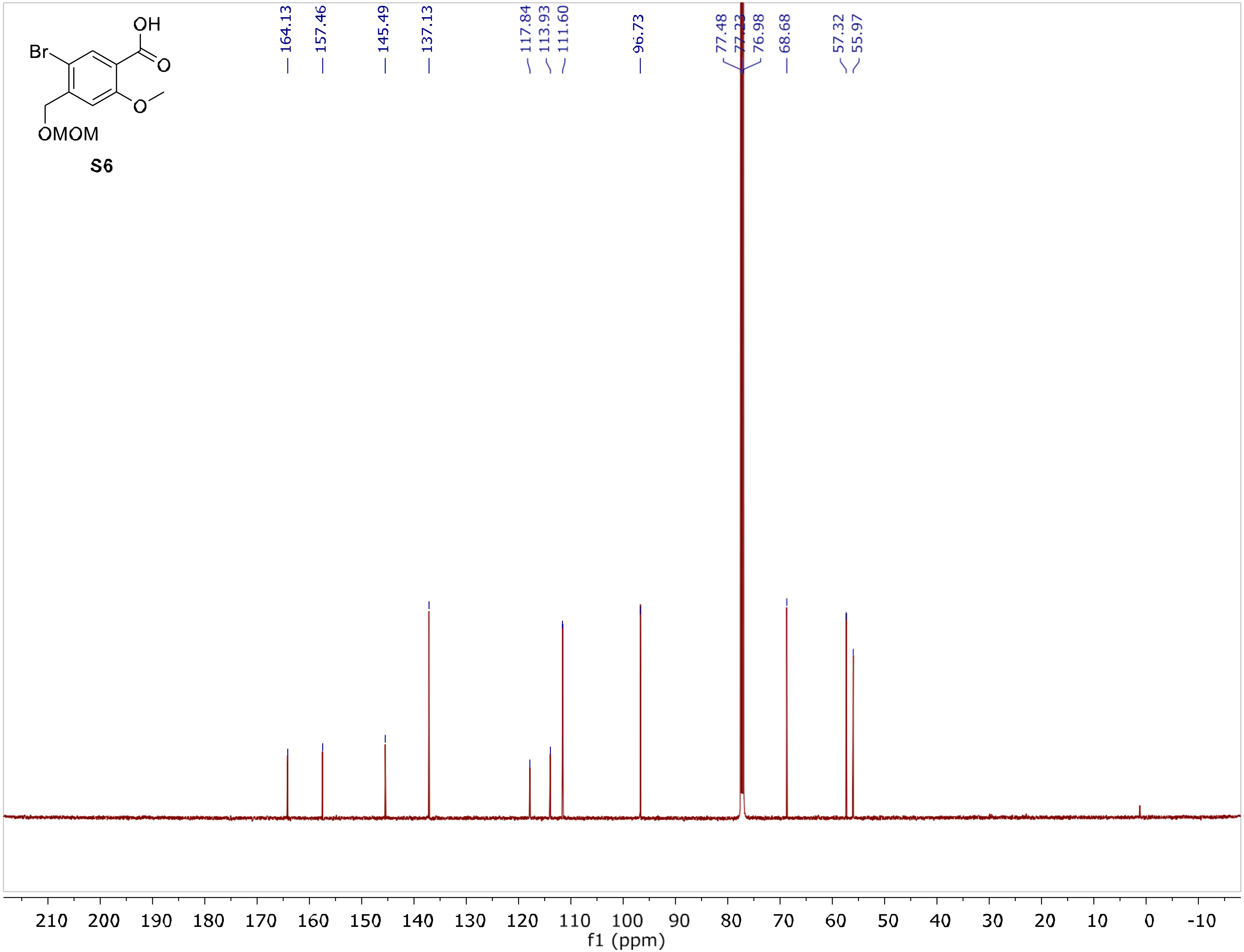

Supplemental Figure 32: H^1 NMR spectra of compound S7.

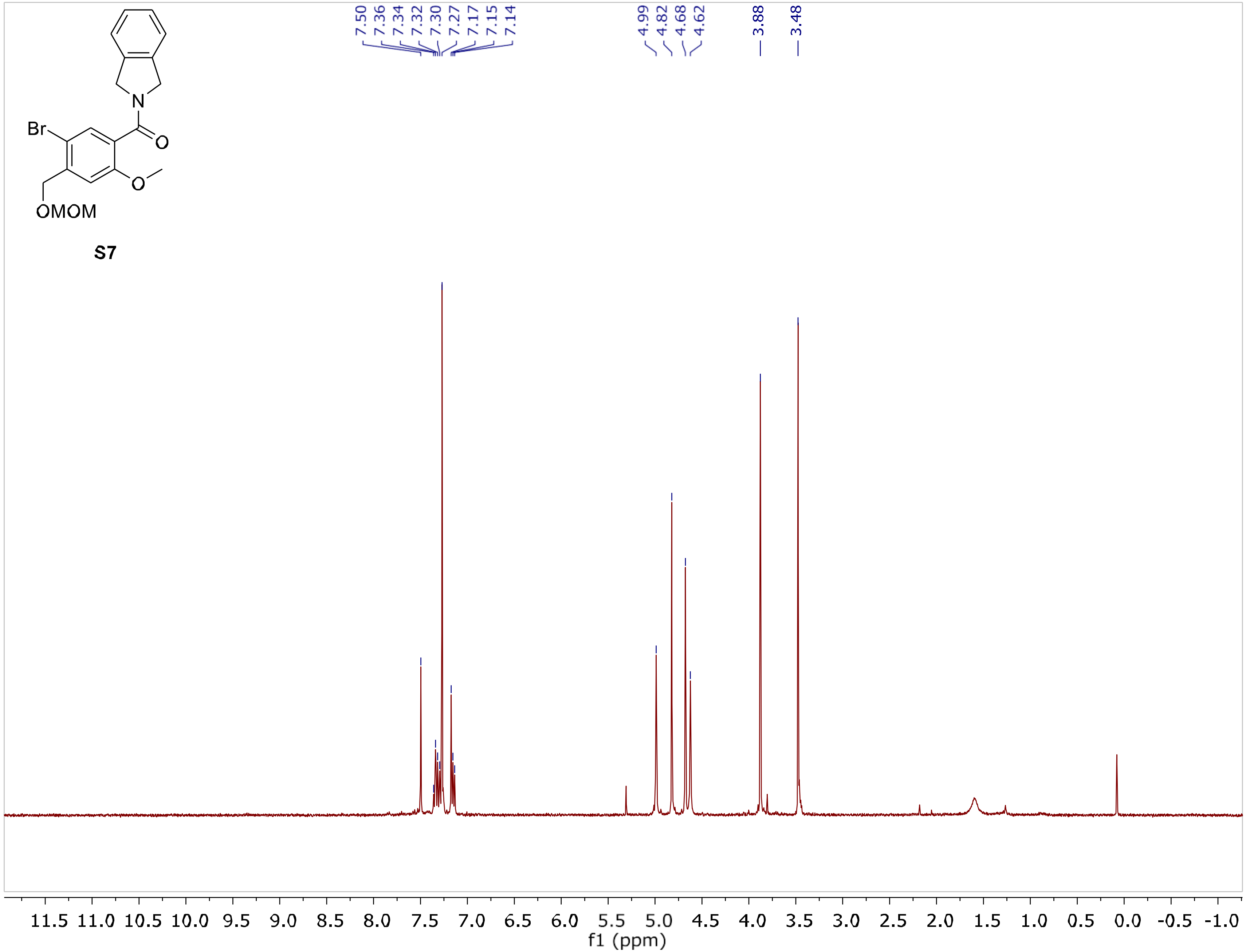

Supplemental Figure 33: C^{13} NMR spectra of compound S7.

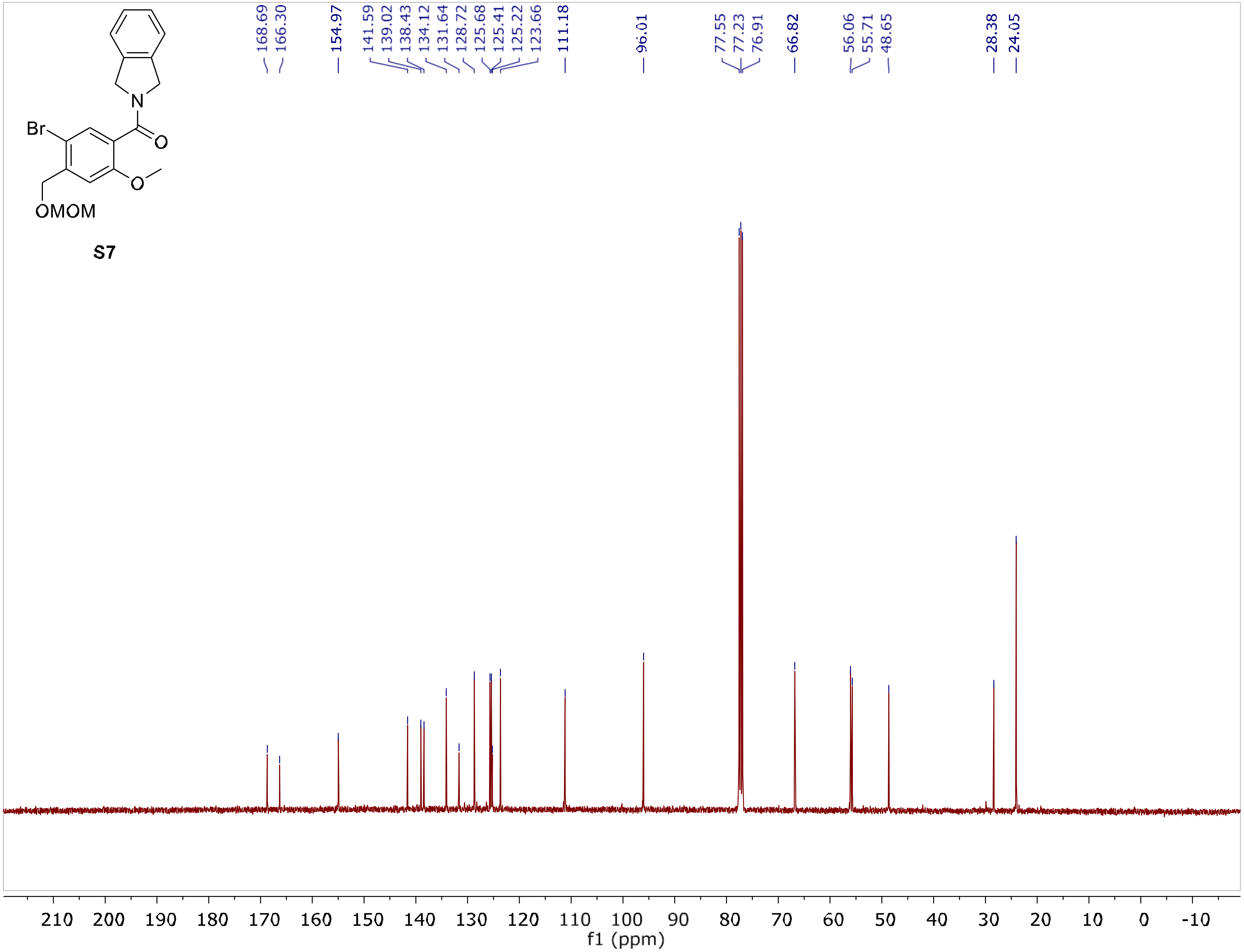

Supplemental Figure 34: H^1 NMR spectra of compound S8.

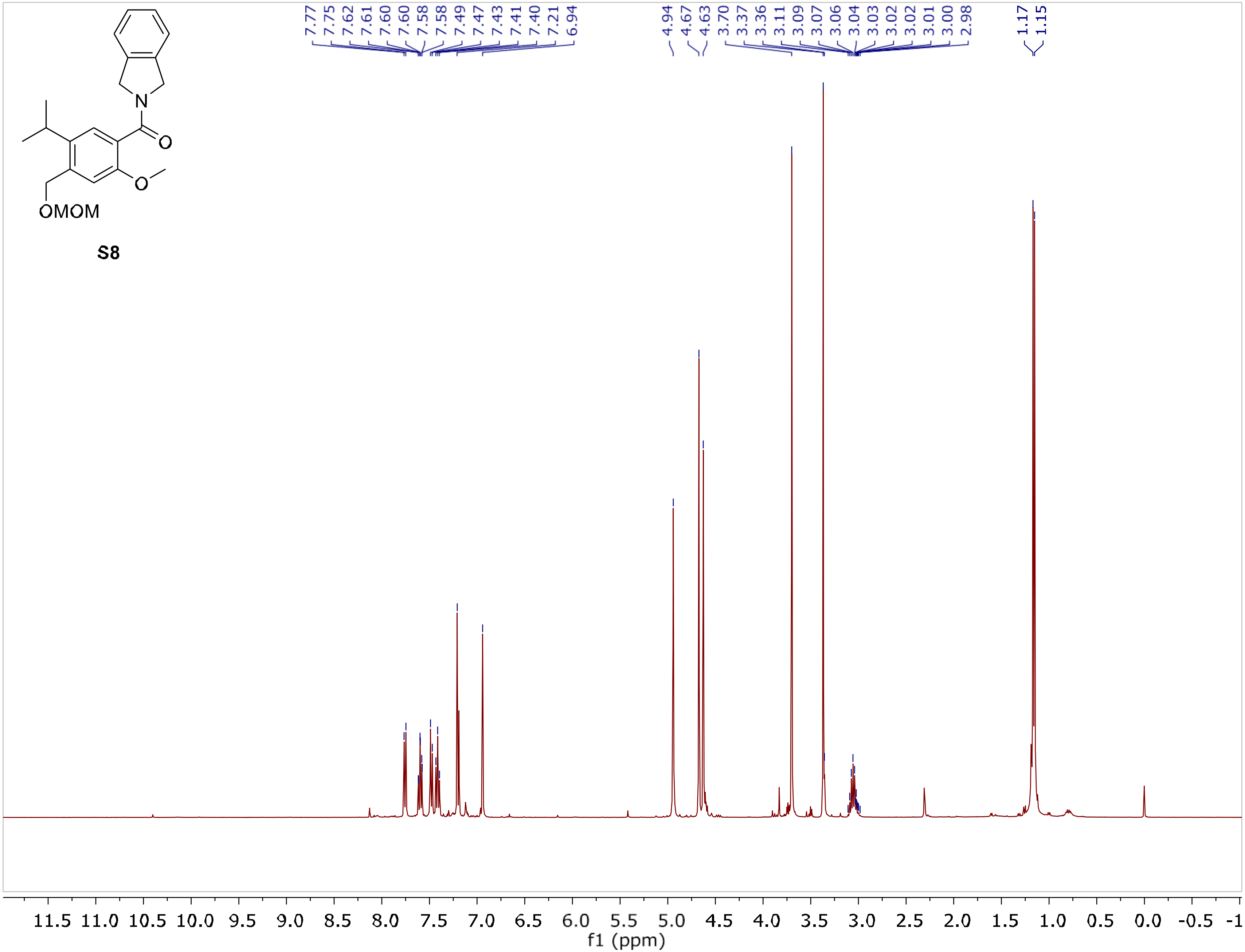

Supplemental Figure 35: H^1 NMR spectra of compound S9.

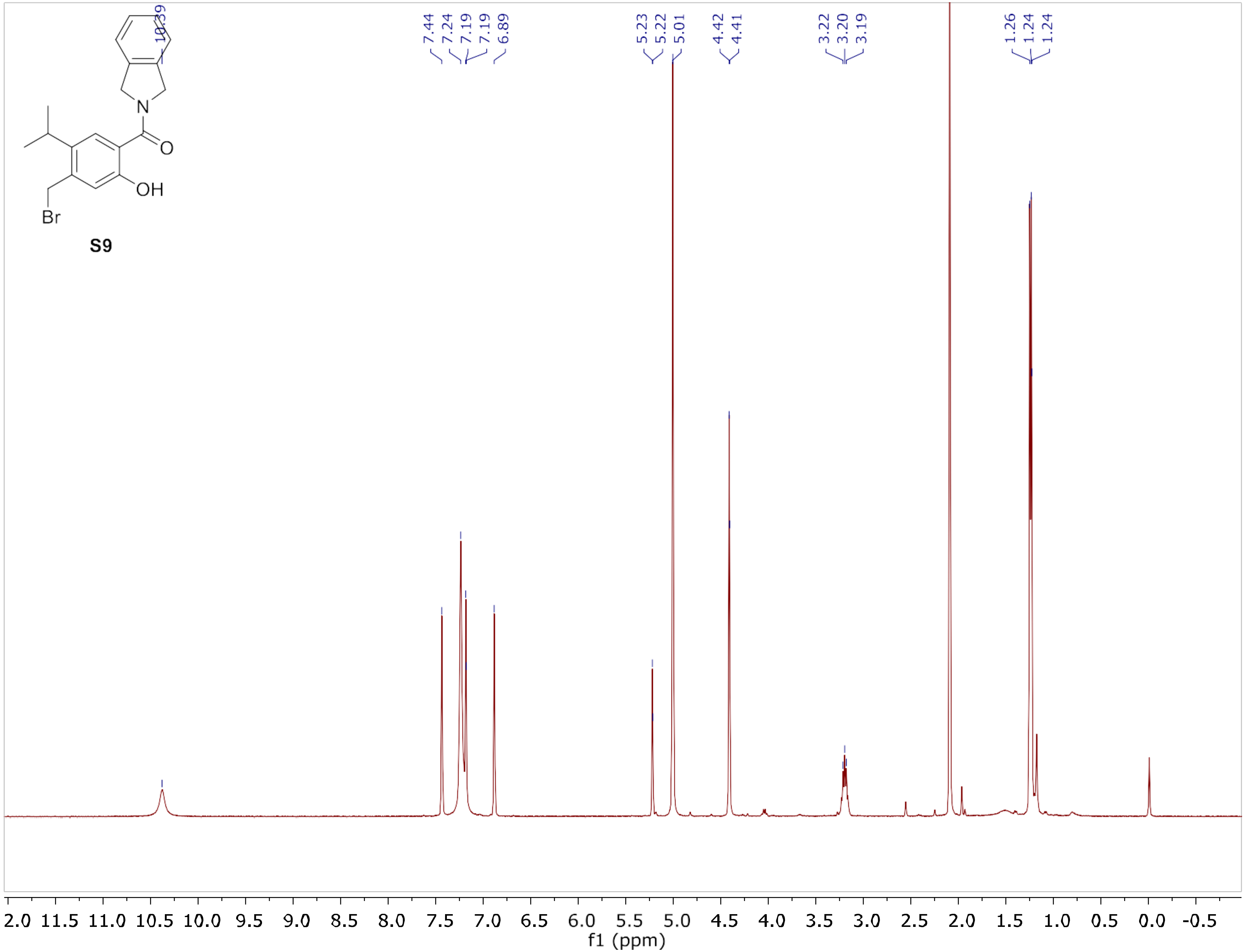

Supplemental Figure 36: C^{13} NMR spectra of compound S9.

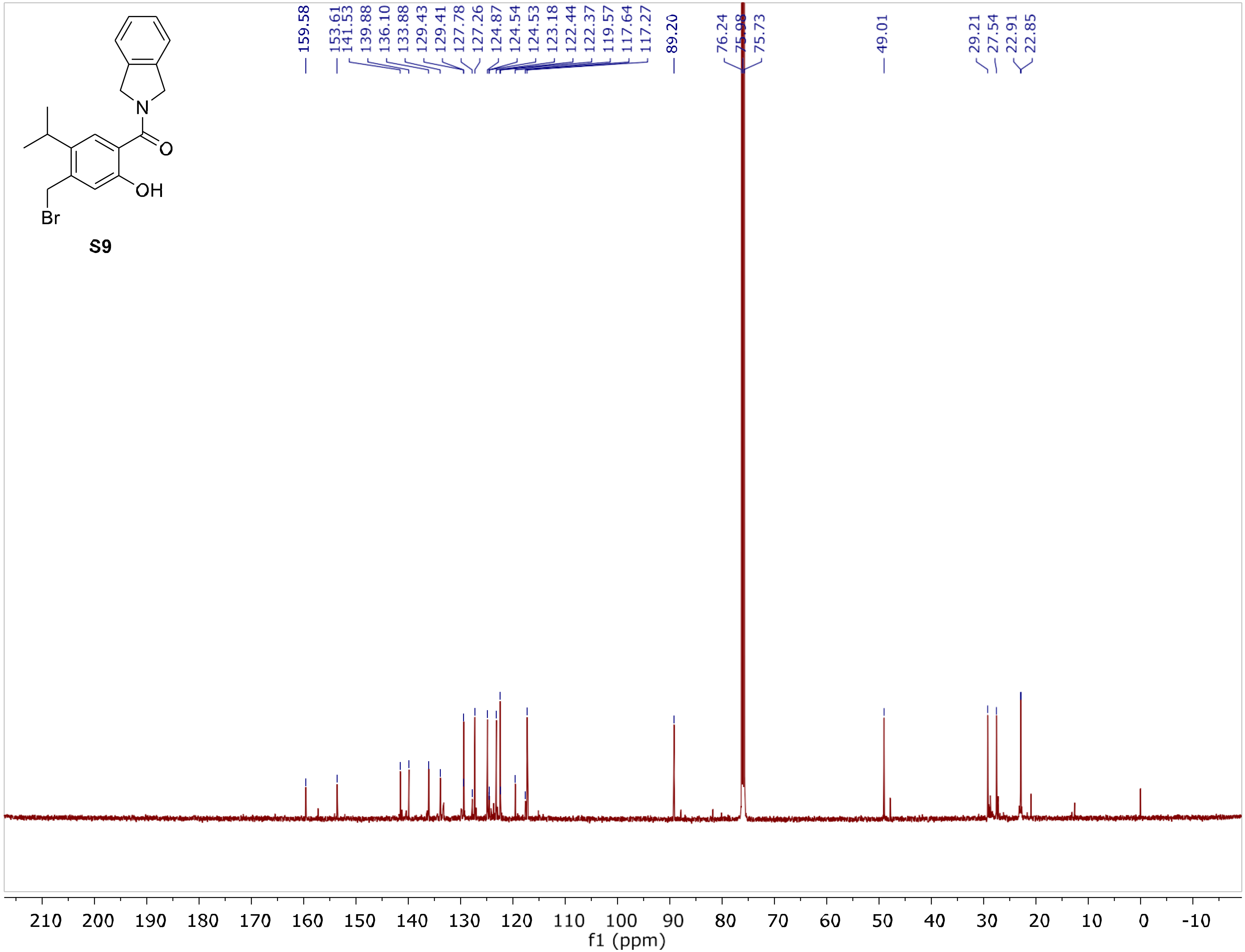

Supplemental Figure 37: H[1] NMR spectra of compound S10.

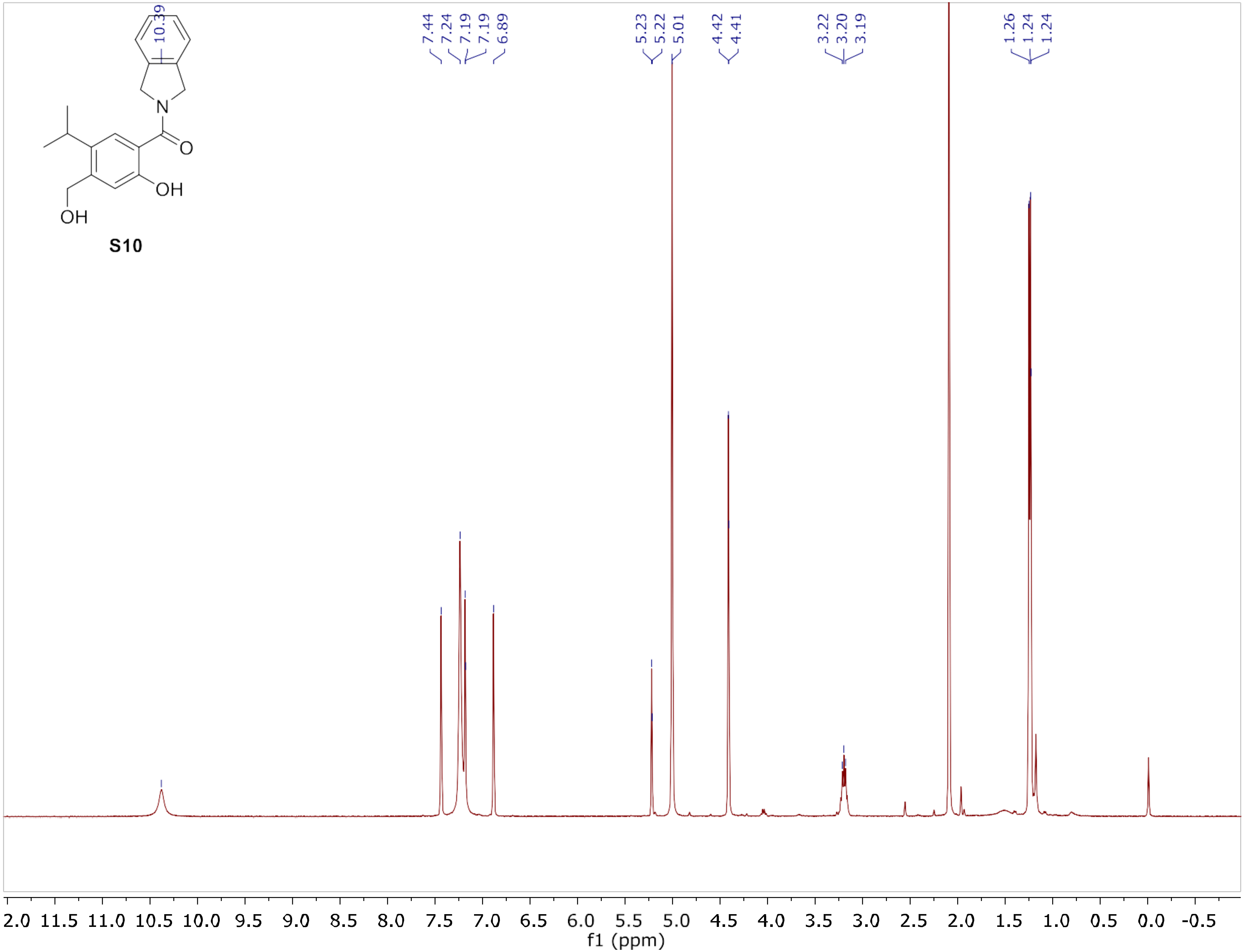

Supplemental Figure 38: C[13] NMR spectra of compound S10.

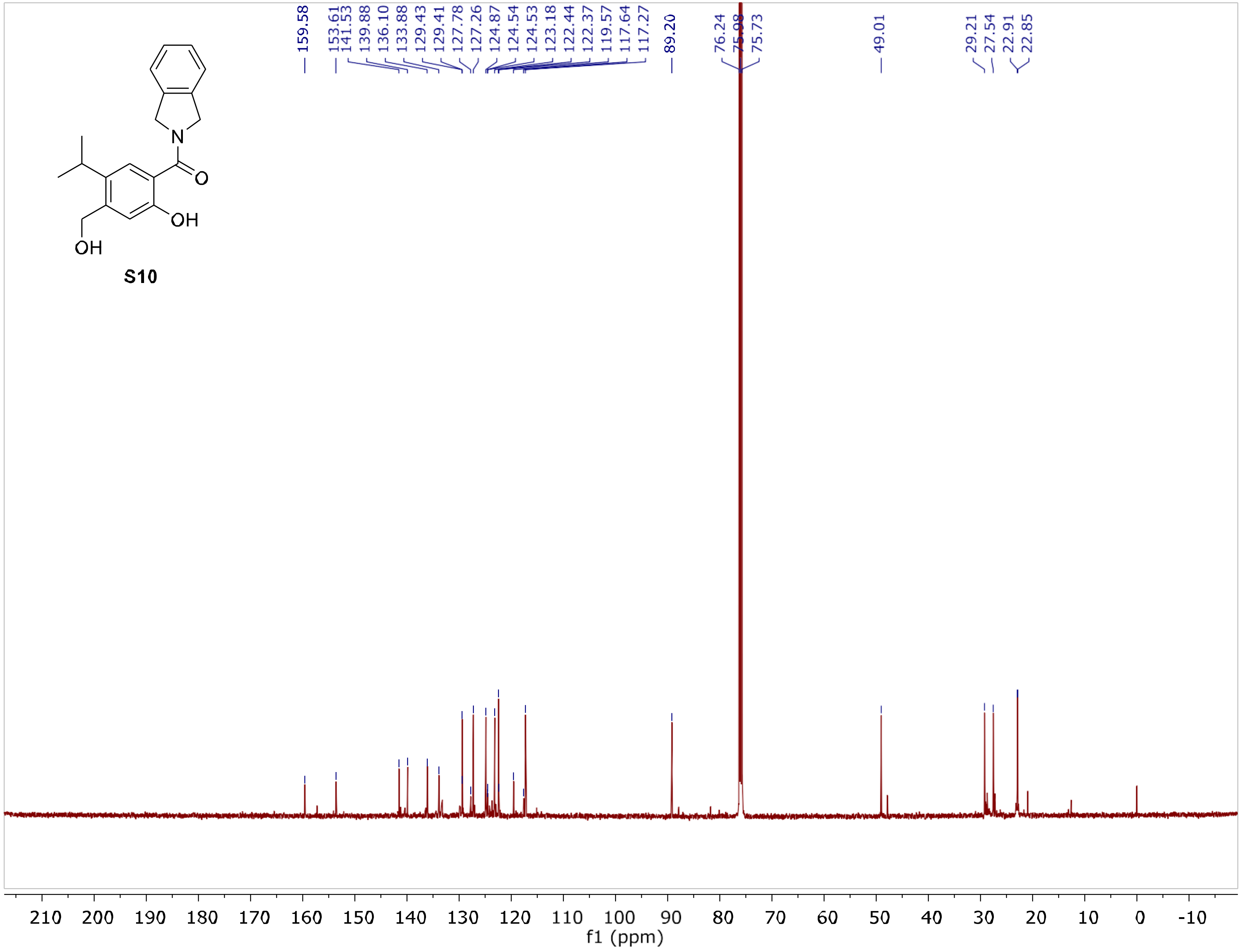

Supplemental Figure 39: H[1] NMR spectra of compound 3.

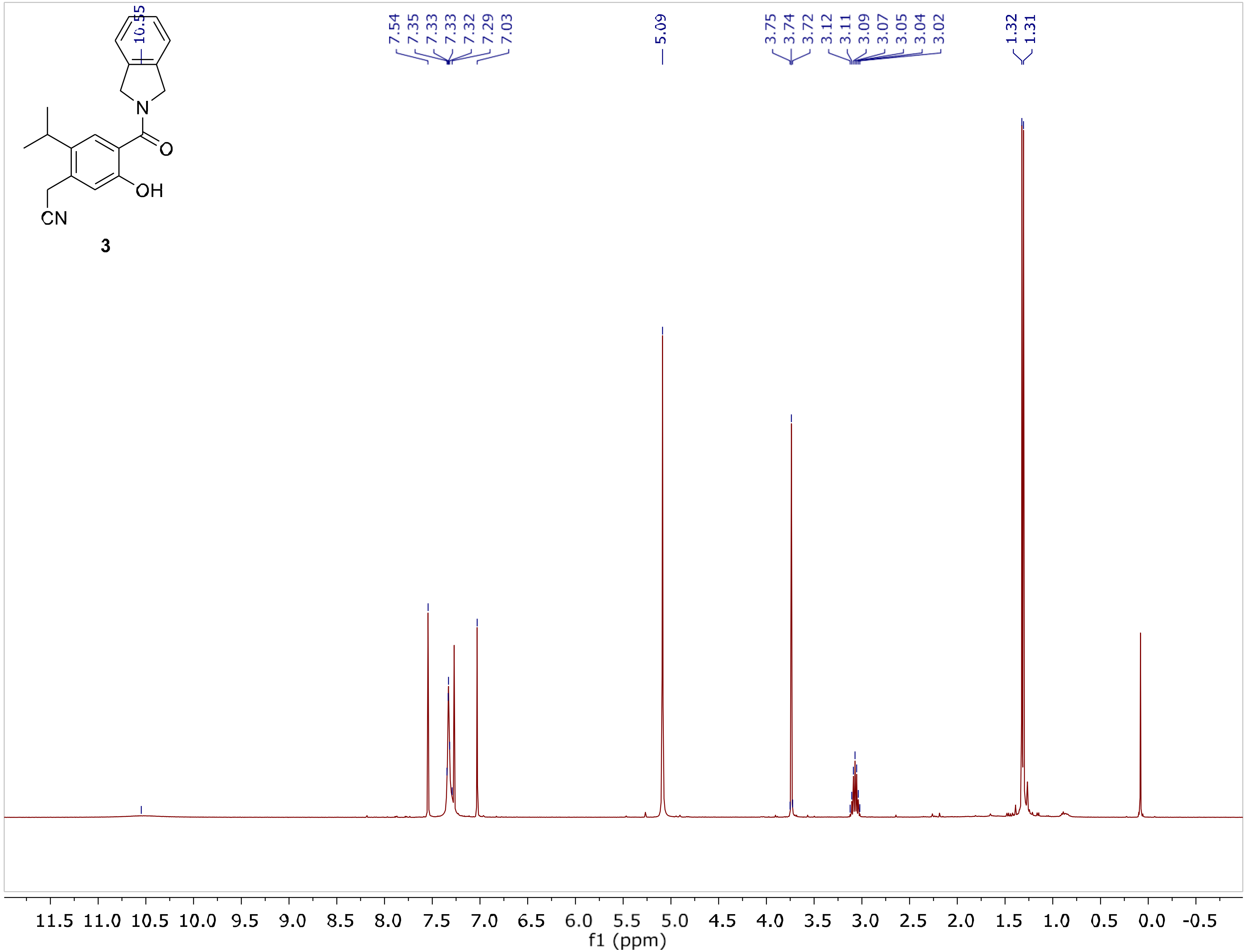

Supplemental Figure 40: C^{13} NMR spectra of compound 3.

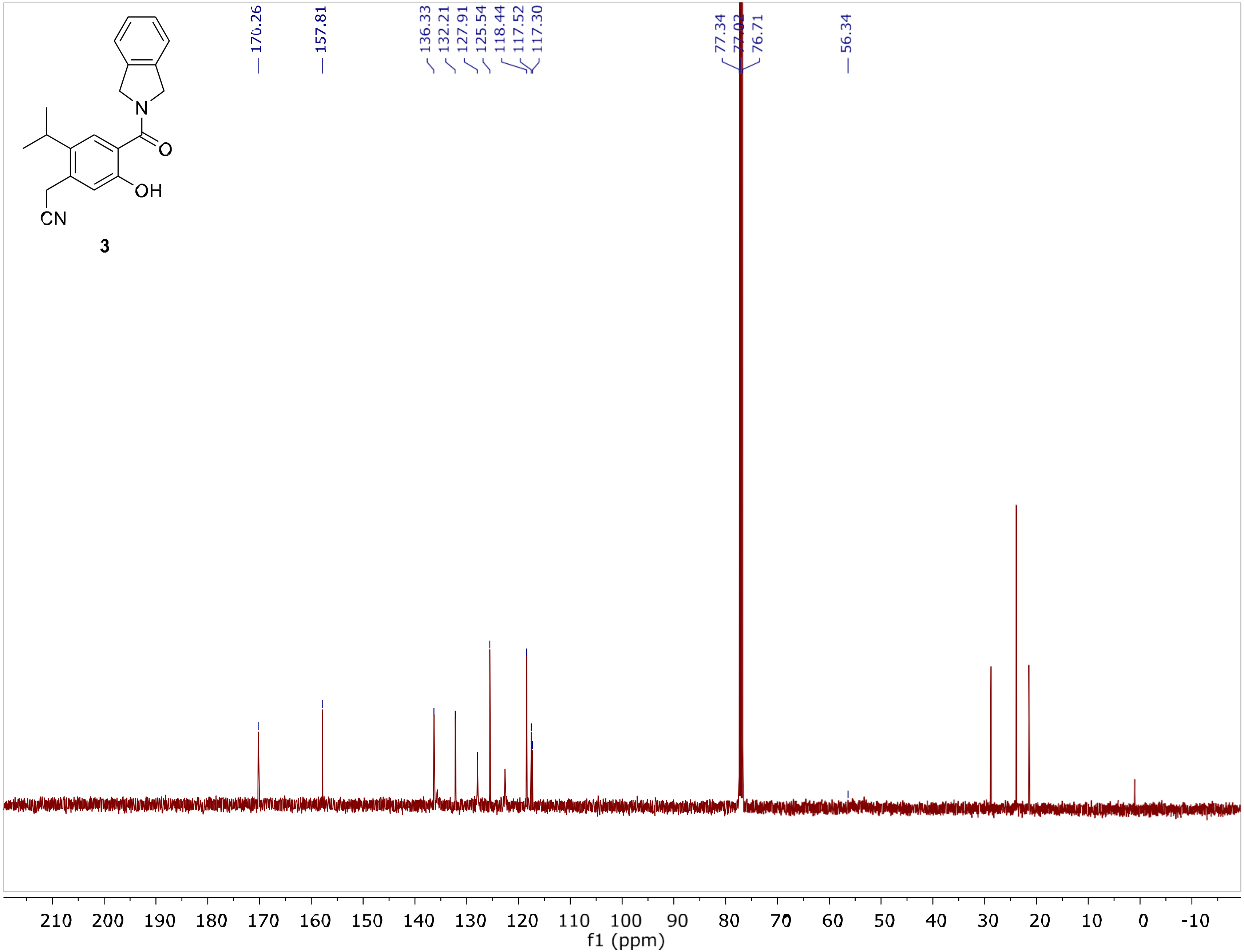

Supplemental Figure 41: H^1 NMR spectra of compound 2.

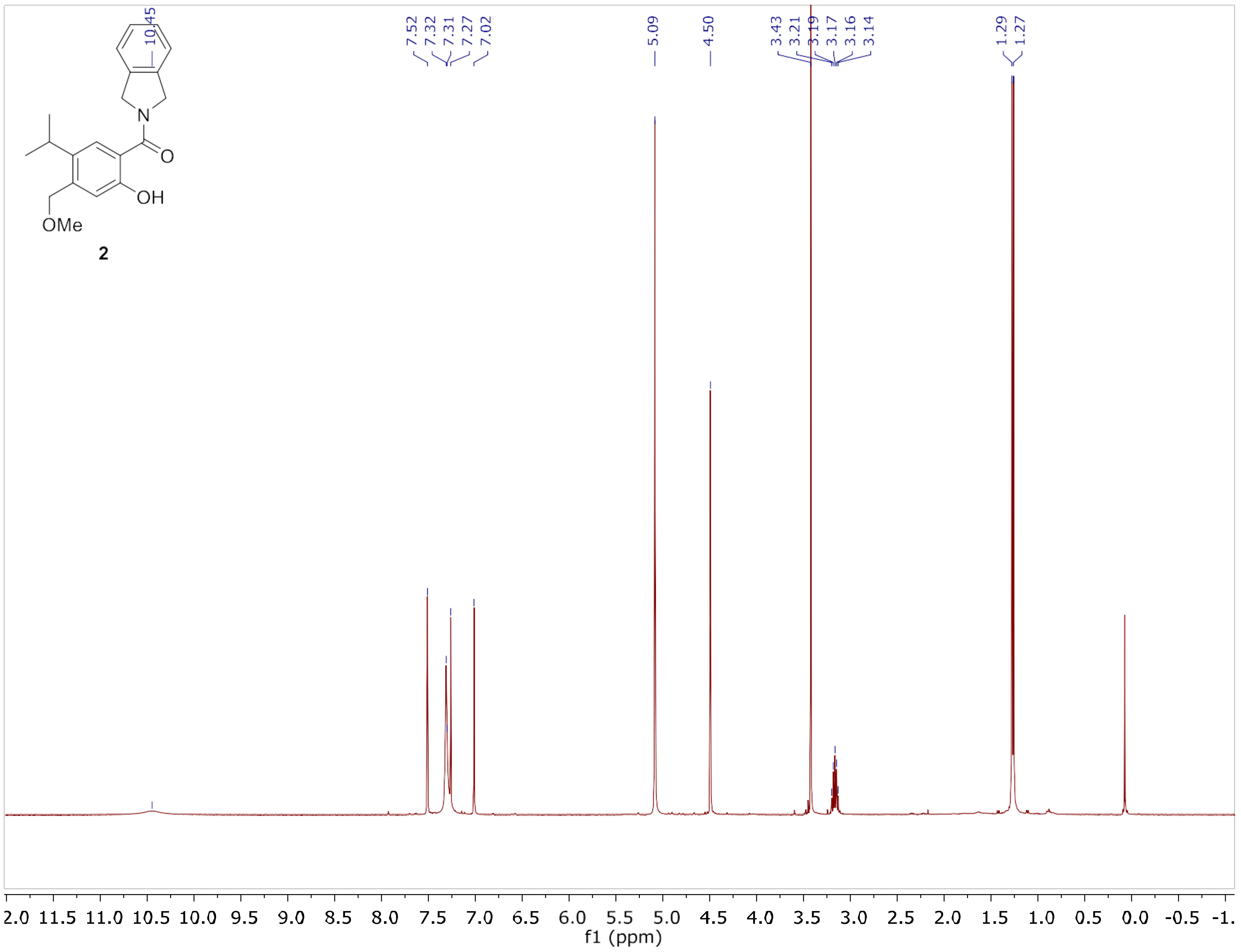

Supplemental Figure 42: C^{13} NMR spectra of compound 2.

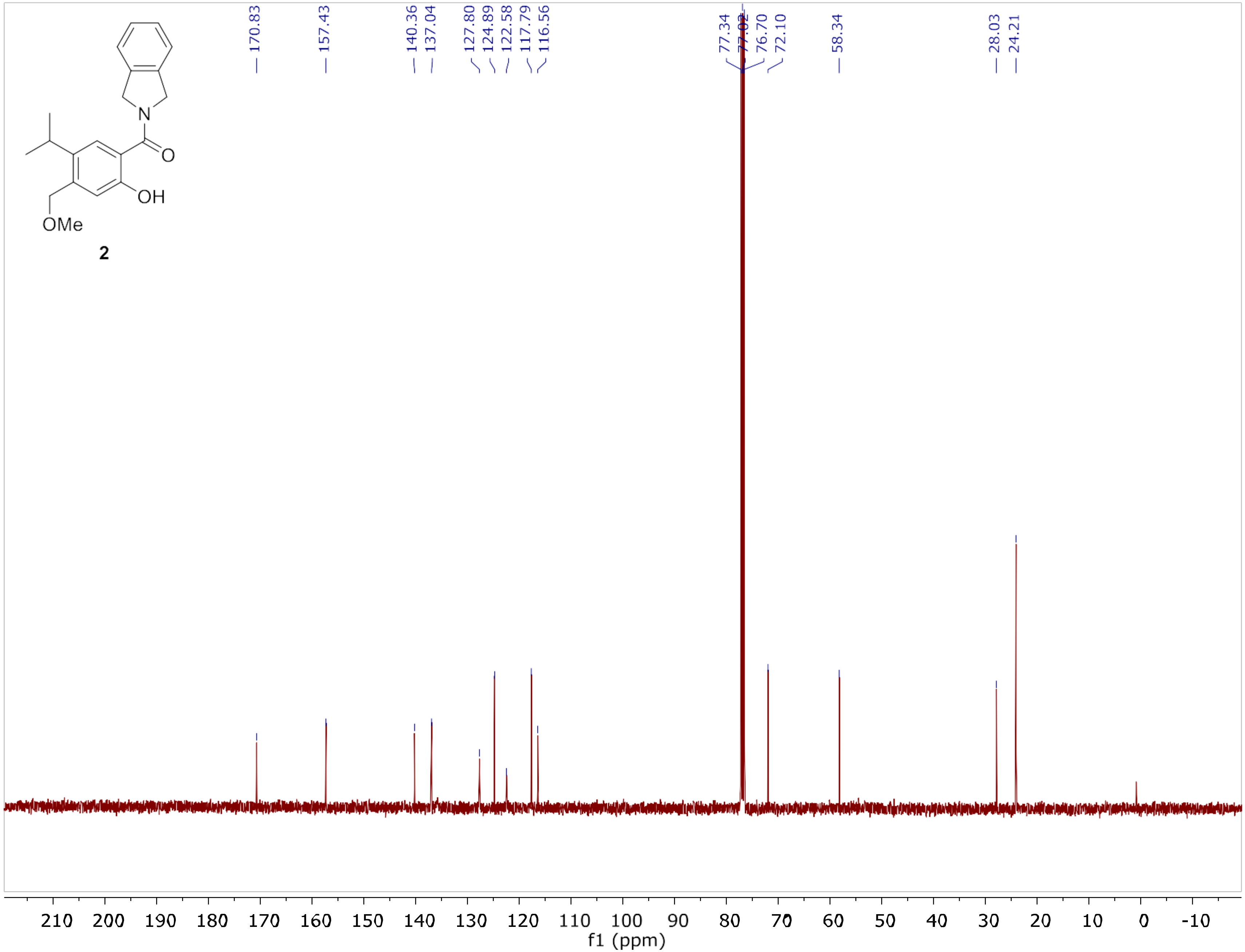

Supplemental Figure 43: H[1] NMR spectra of compound S11.

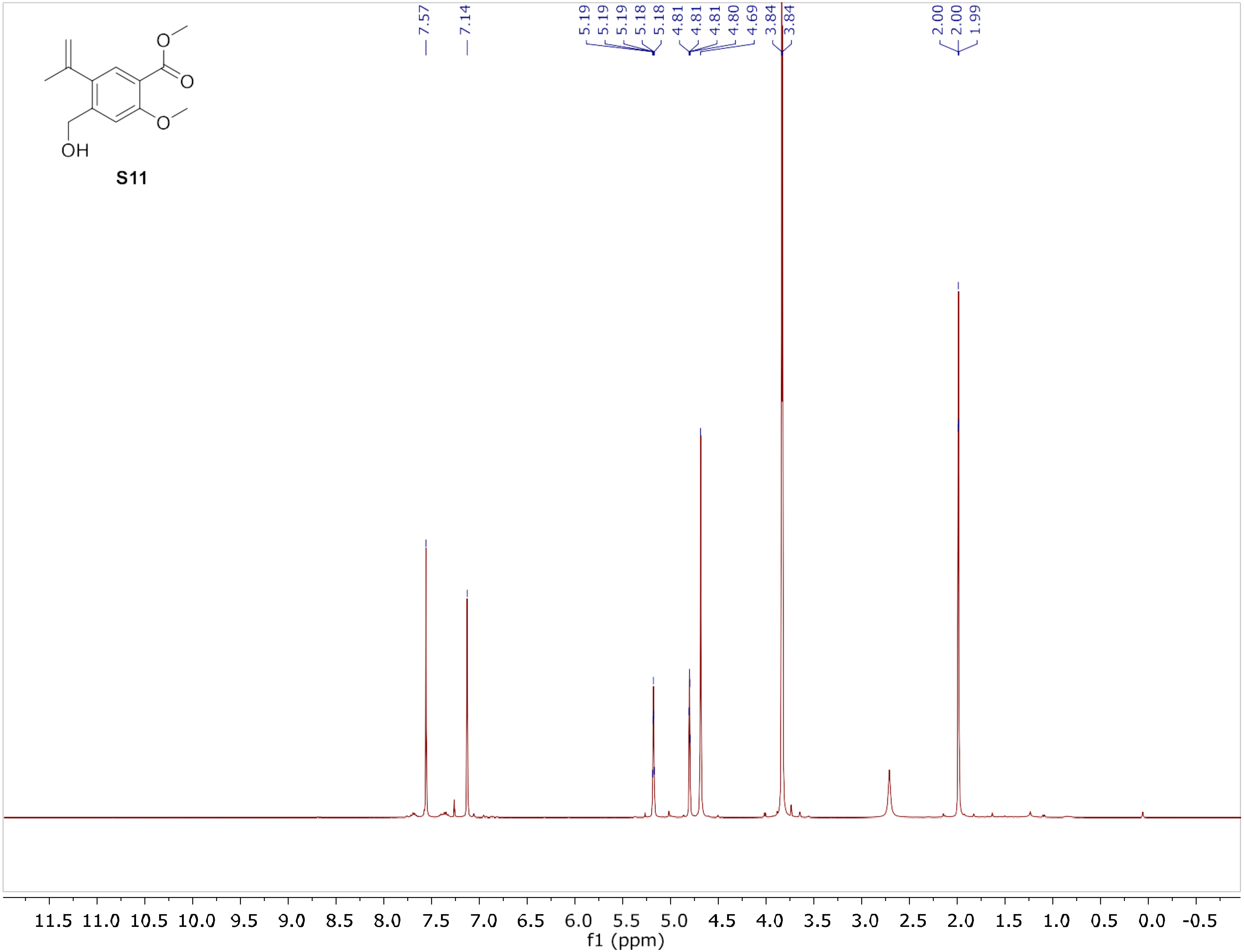

Supplemental Figure 44: C^{13} NMR spectra of compound S11.

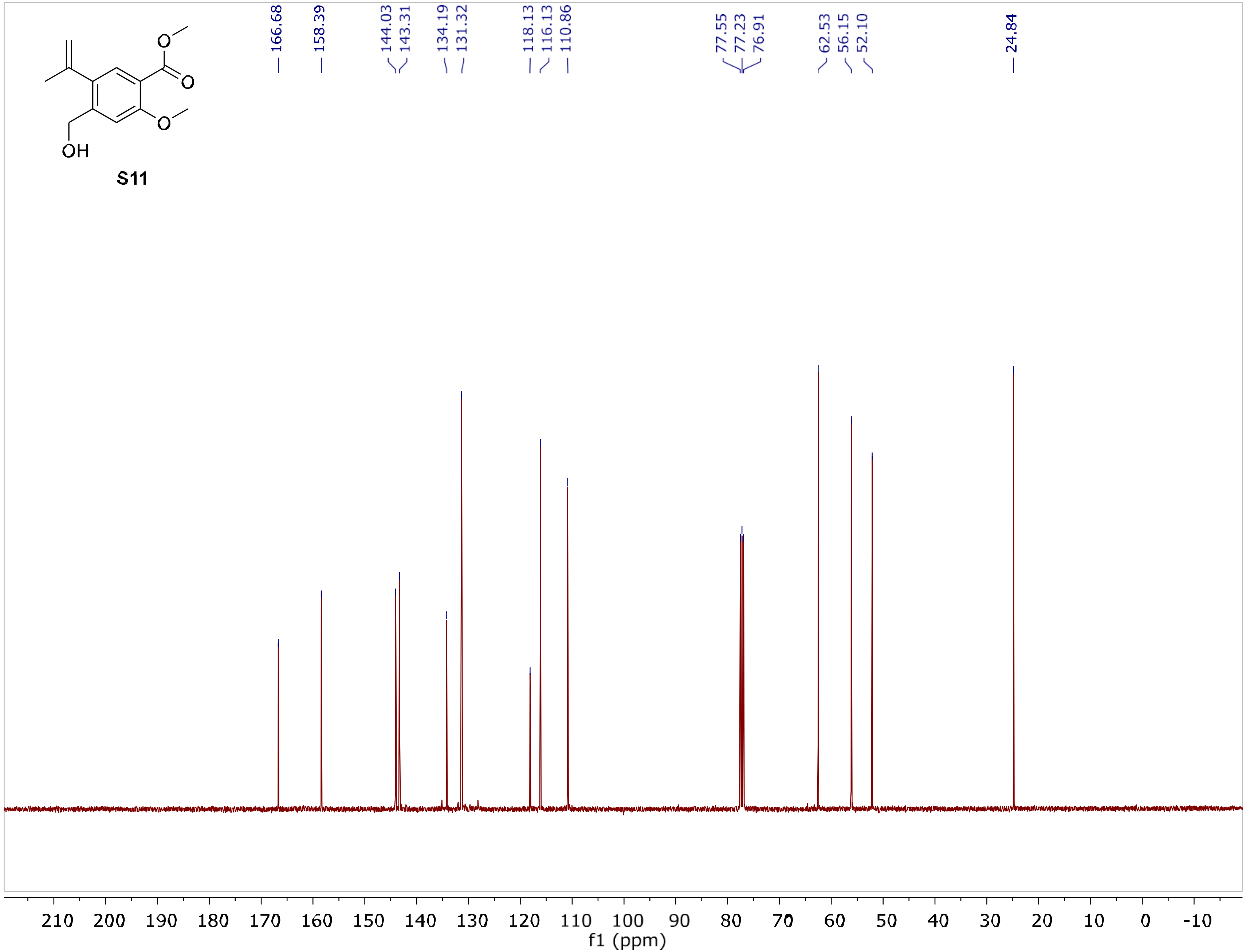

Supplemental Figure 45: H^1 NMR spectra of compound S12.

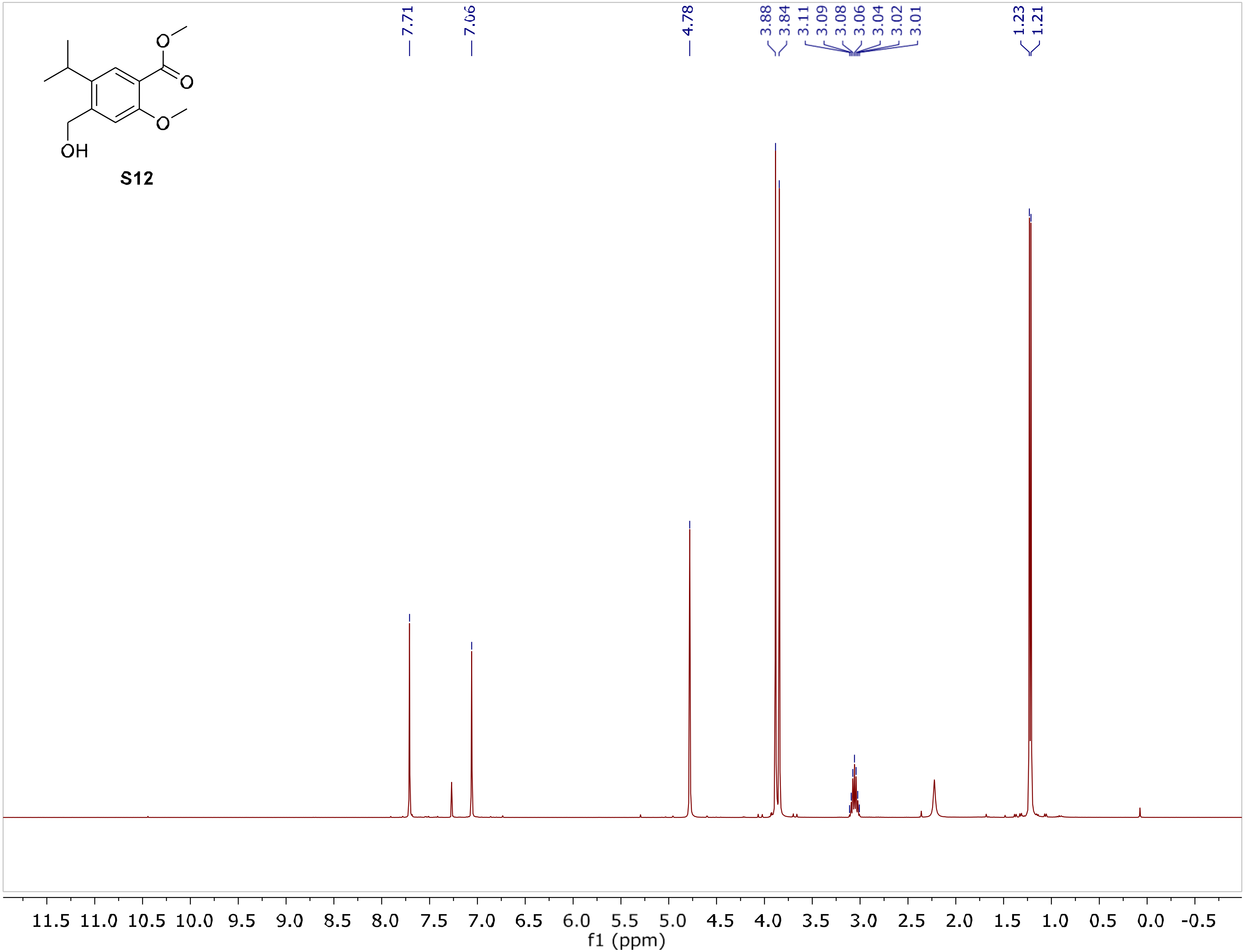

Supplemental Figure 46: C^{13} NMR spectra of compound S12.

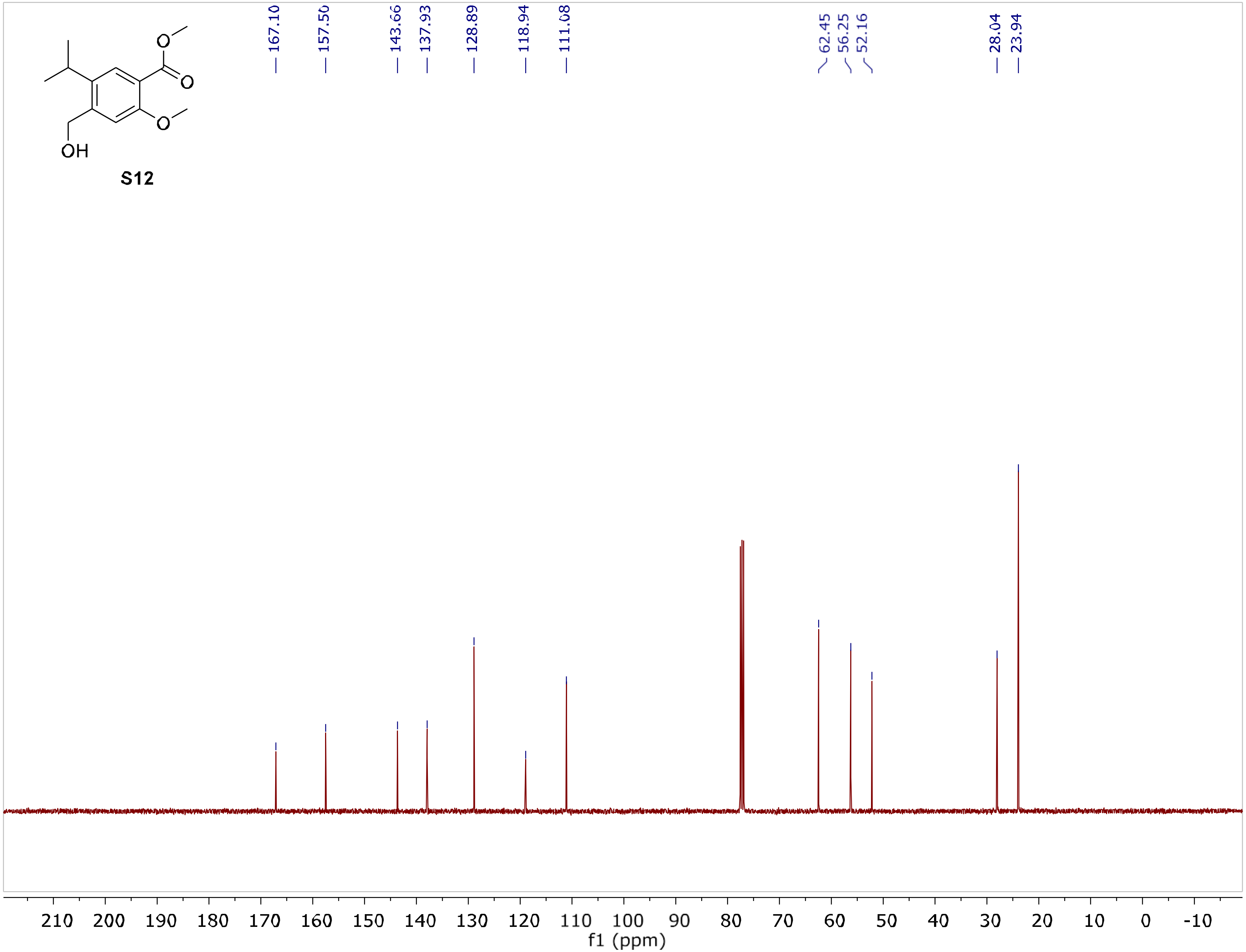

Supplemental Figure 47: H^1 NMR spectra of compound S13.

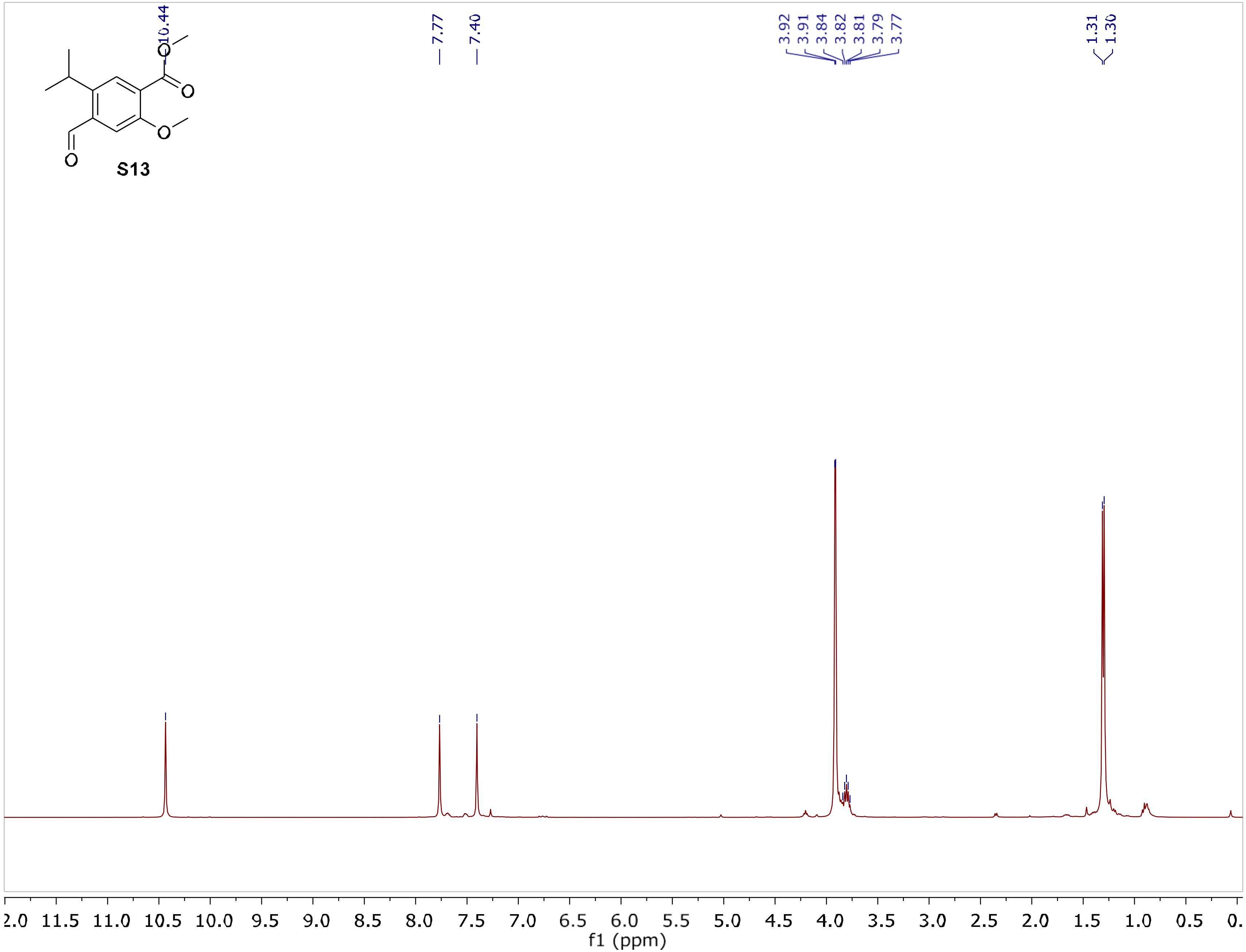

Supplemental Figure 48: C^{13} NMR spectra of compound S13.

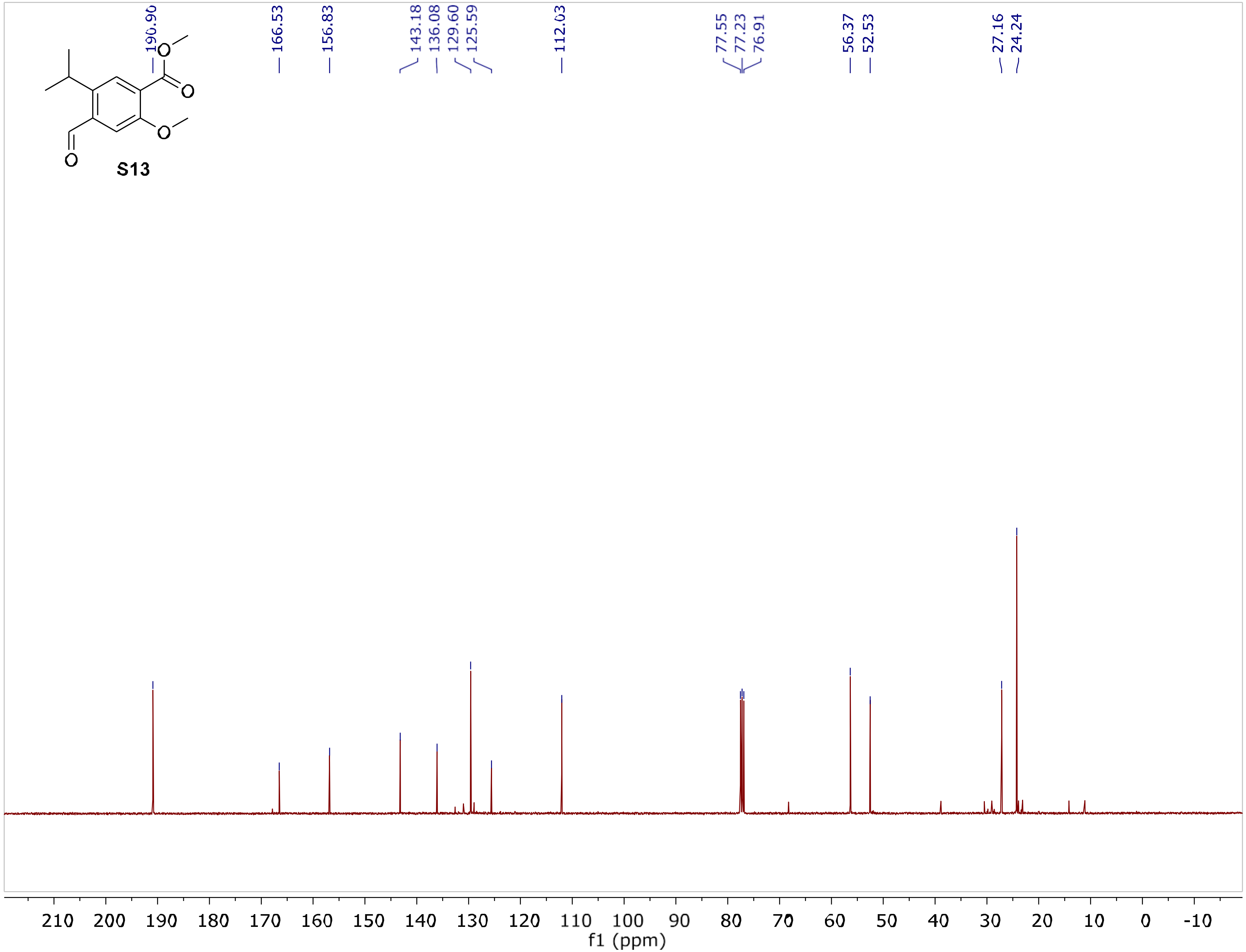

Supplemental Figure 49: H[1] NMR spectra of compound S14.

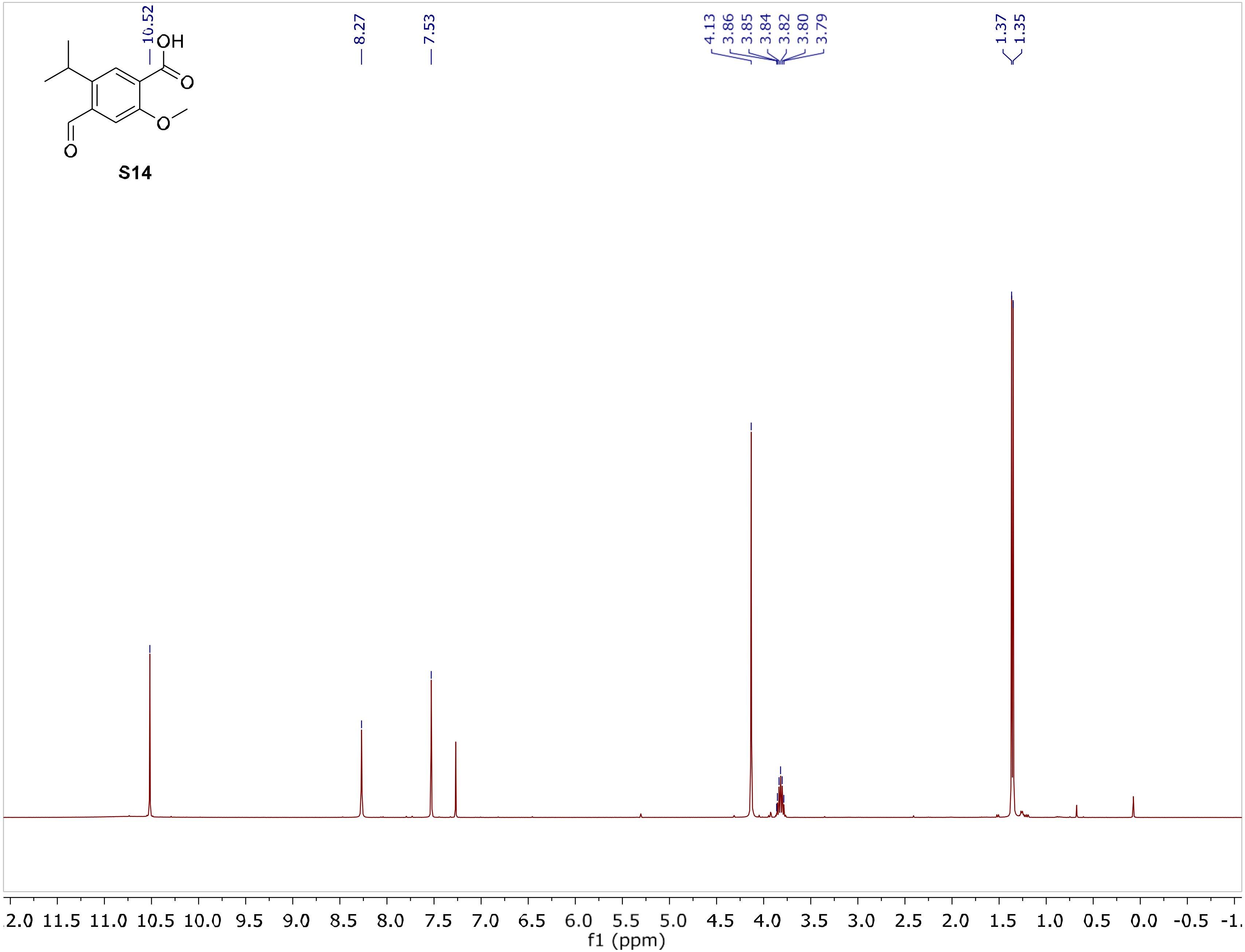

Supplemental Figure 50: C^{13} NMR spectra of compound S14.

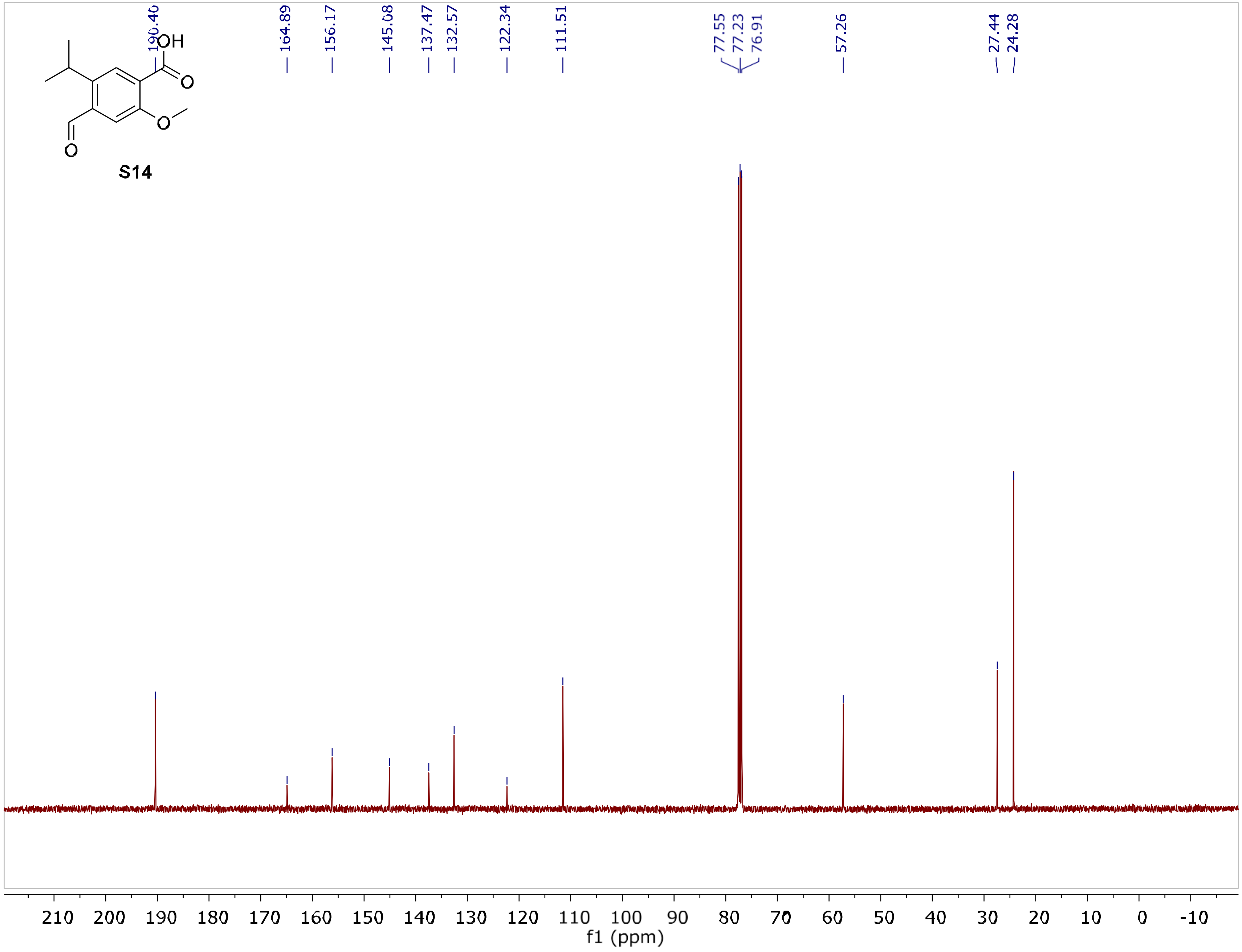

Supplemental Figure 51: H1 NMR spectra of compound S15.

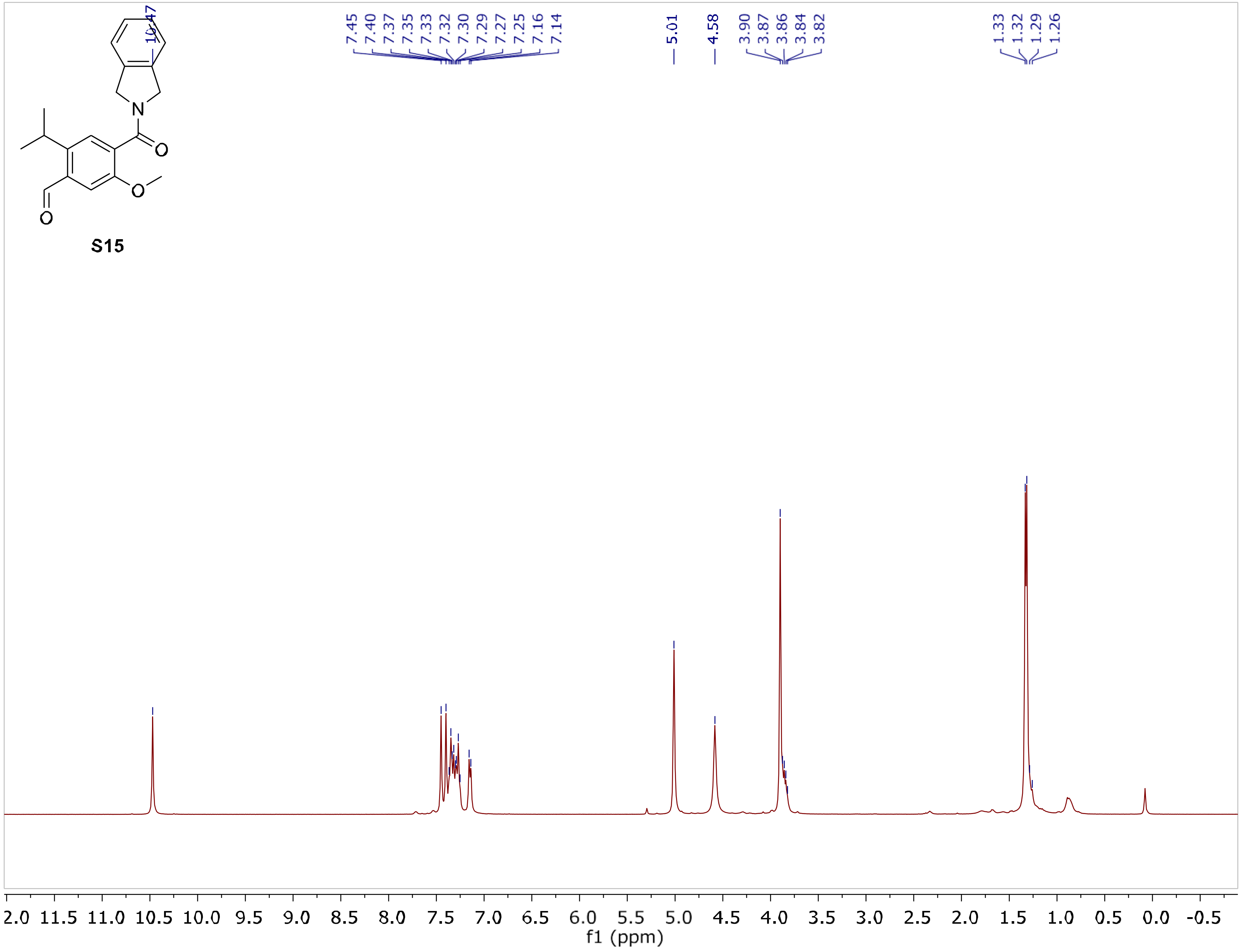

Supplemental Figure 52: C^{13} NMR spectra of compound S15.

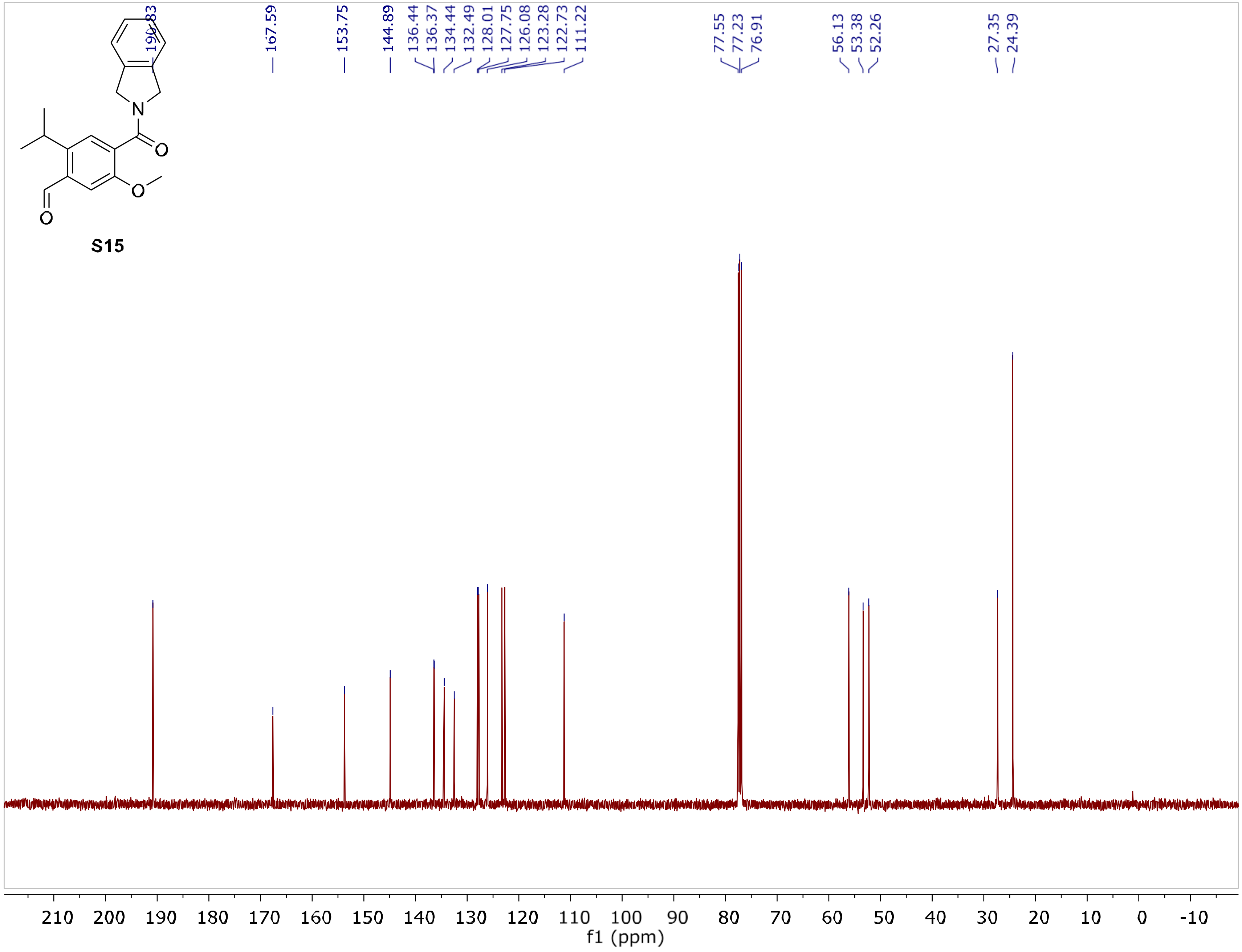

Supplemental Figure 53: H^1 NMR spectra of compound 4.

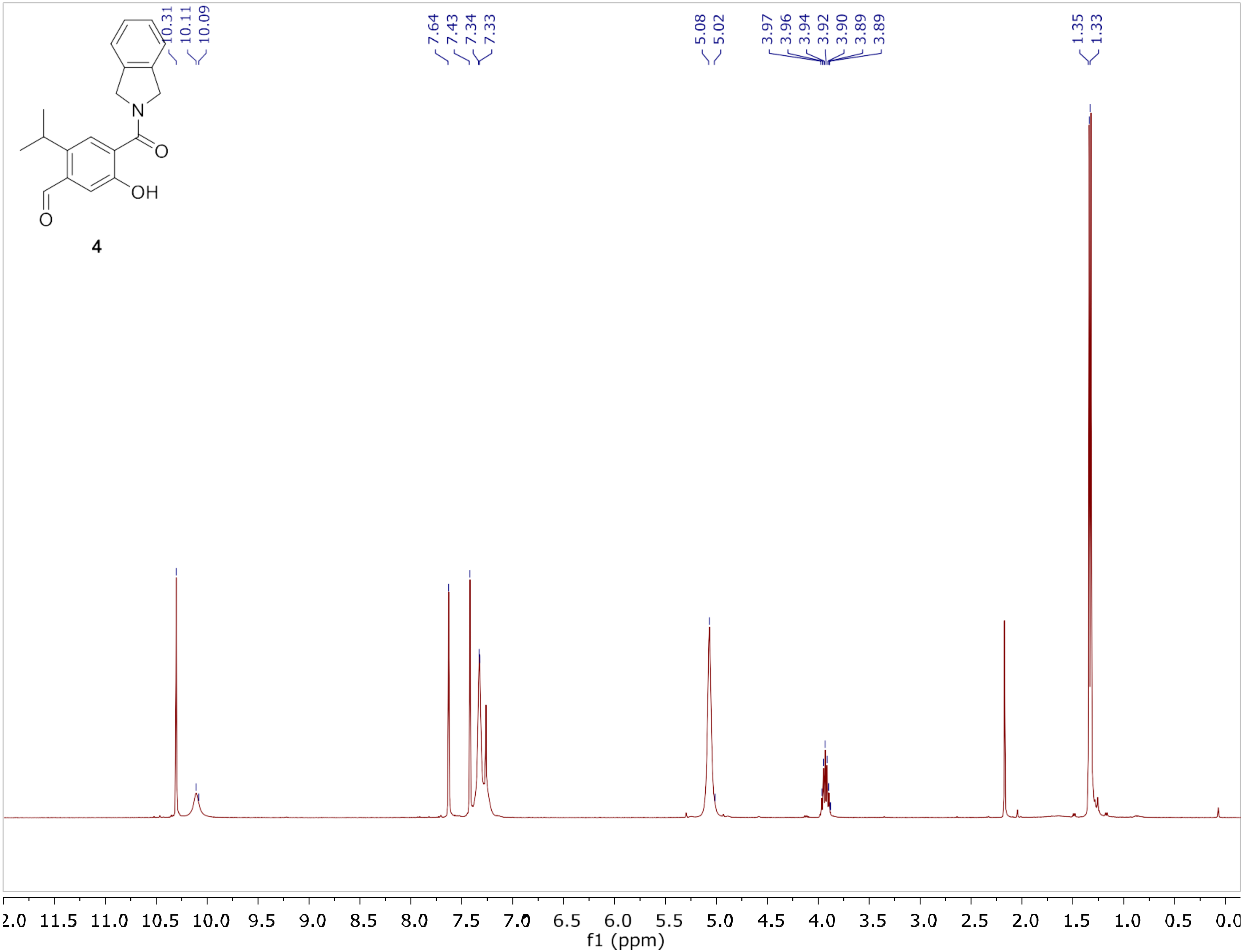

Supplemental Figure 54: H^1 NMR spectra of compound S17.

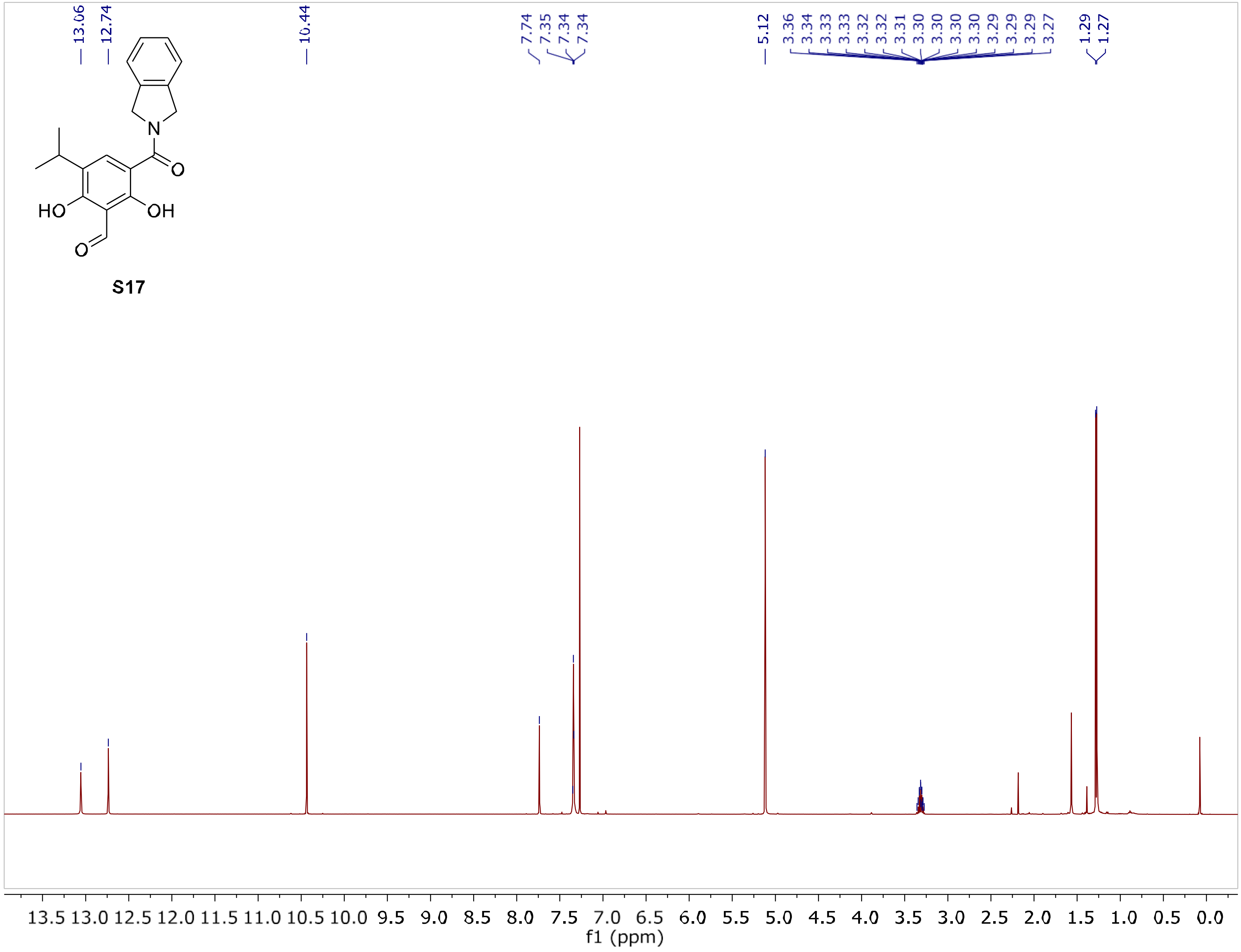

Supplemental Figure 55: C^{13} NMR spectra of compound S17.

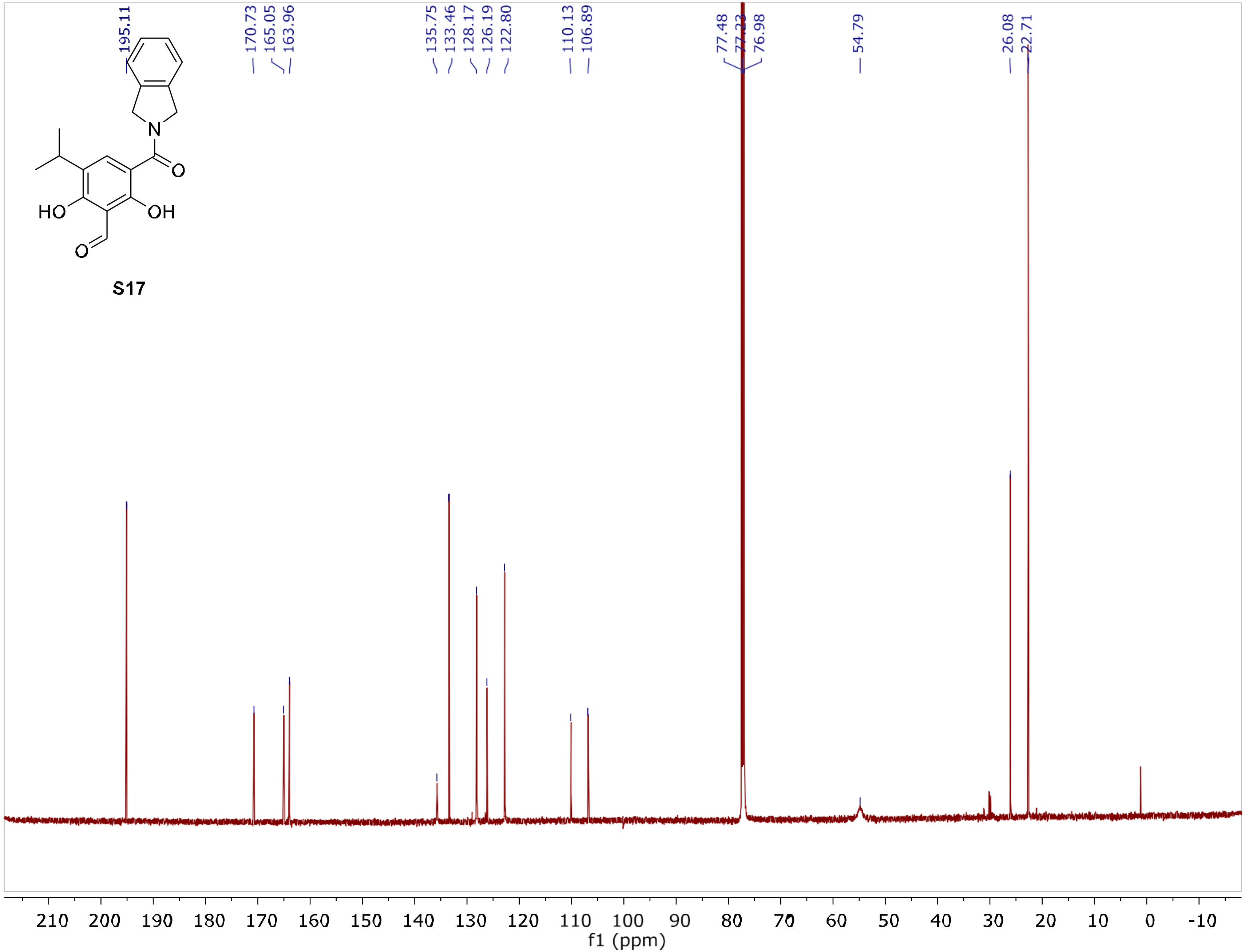

Supplemental Figure 56: H[1] NMR spectra of compound 5.

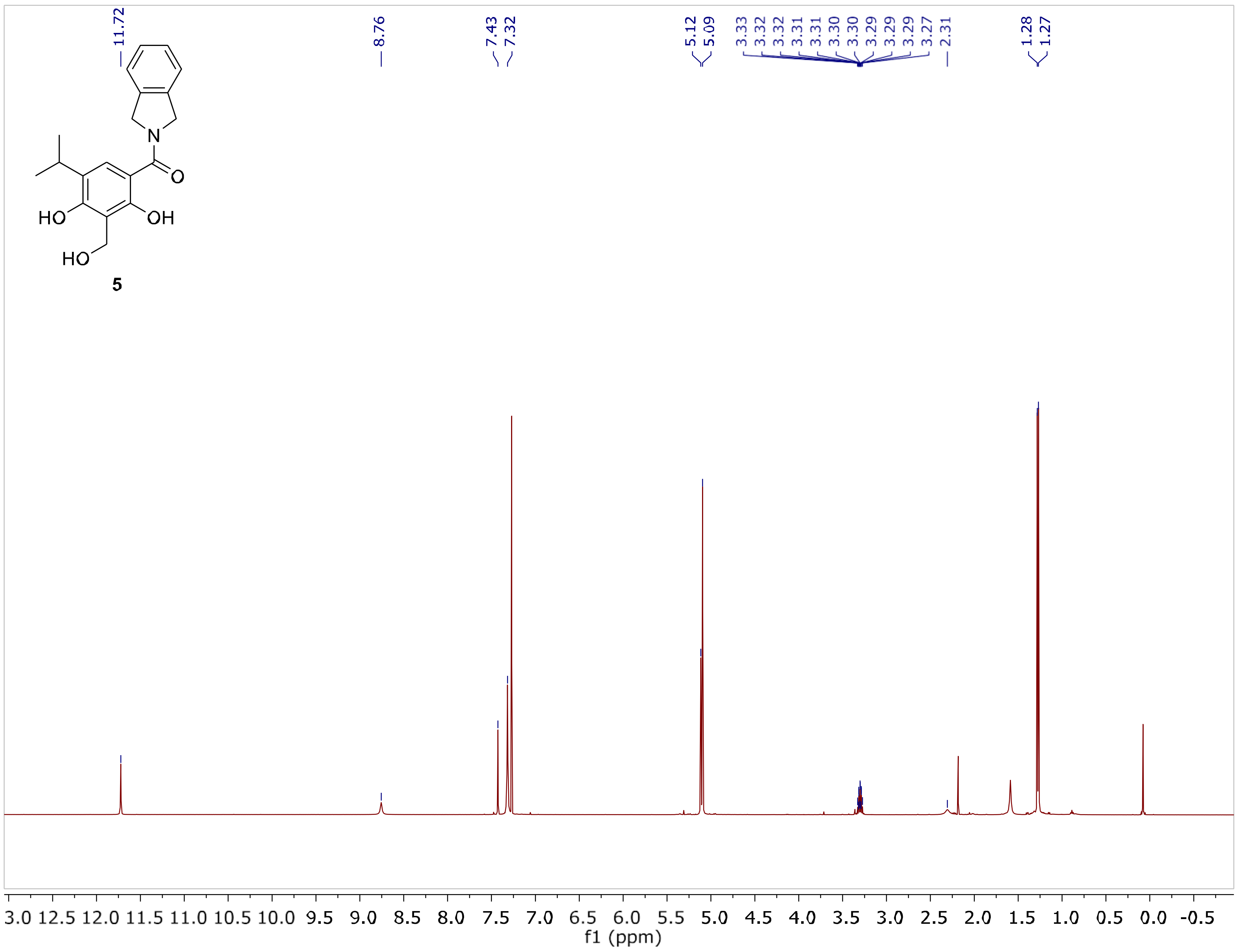

Supplemental Figure 57: C^{13} NMR spectra of compound 5.

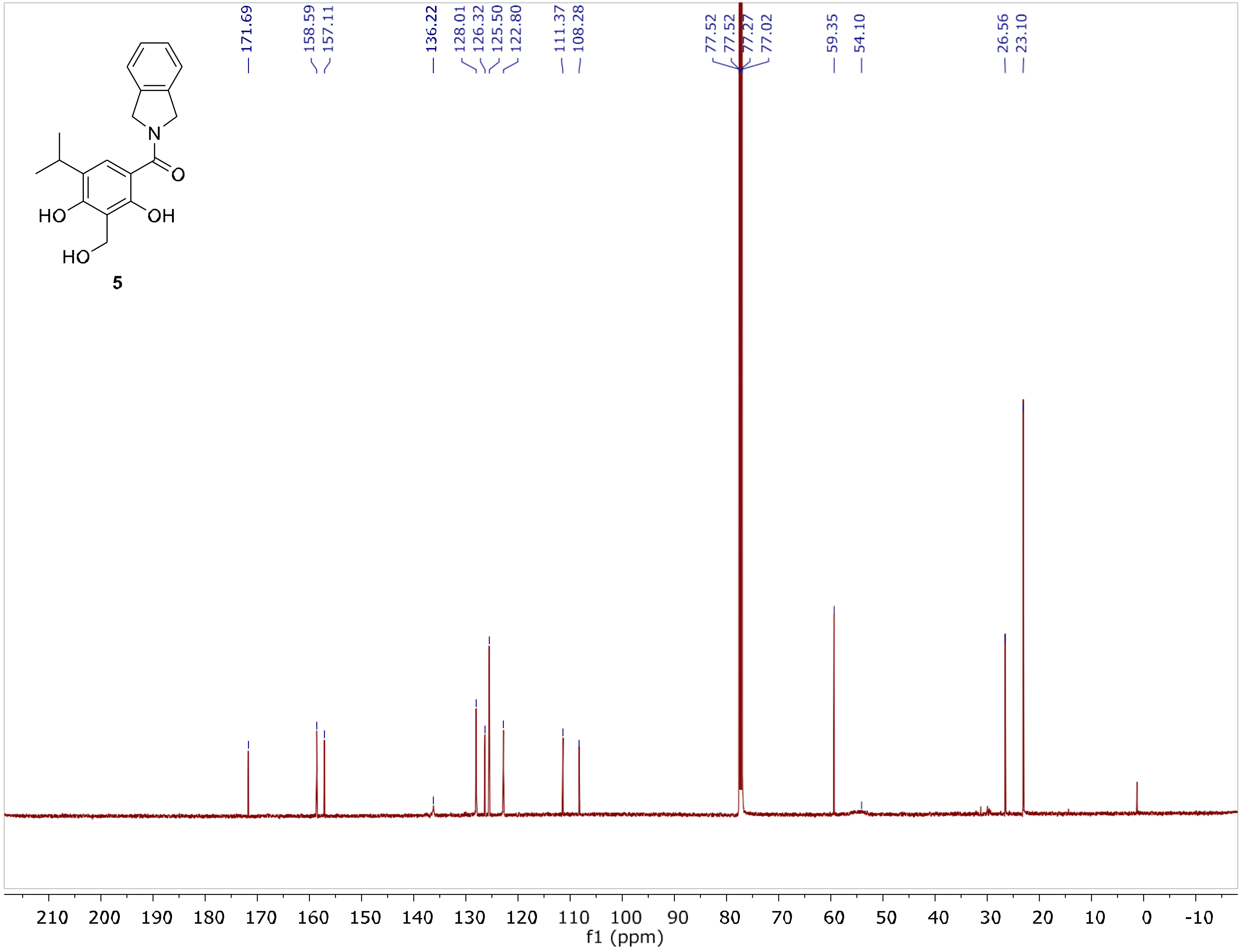

Supplemental Figure 58: H[1] NMR spectra of compound 6a.

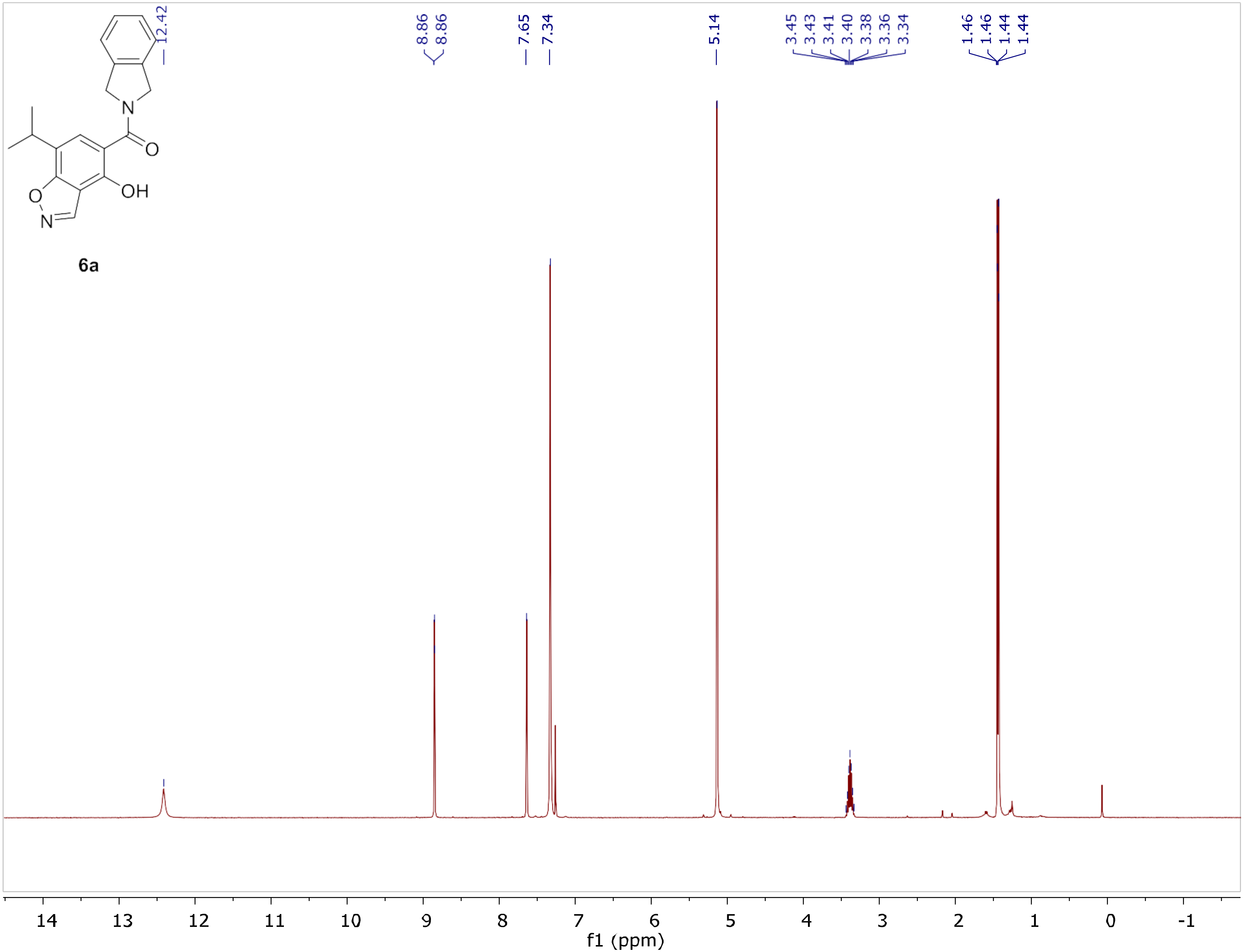

Supplemental Figure 59: C^{13} NMR spectra of compound 6a.

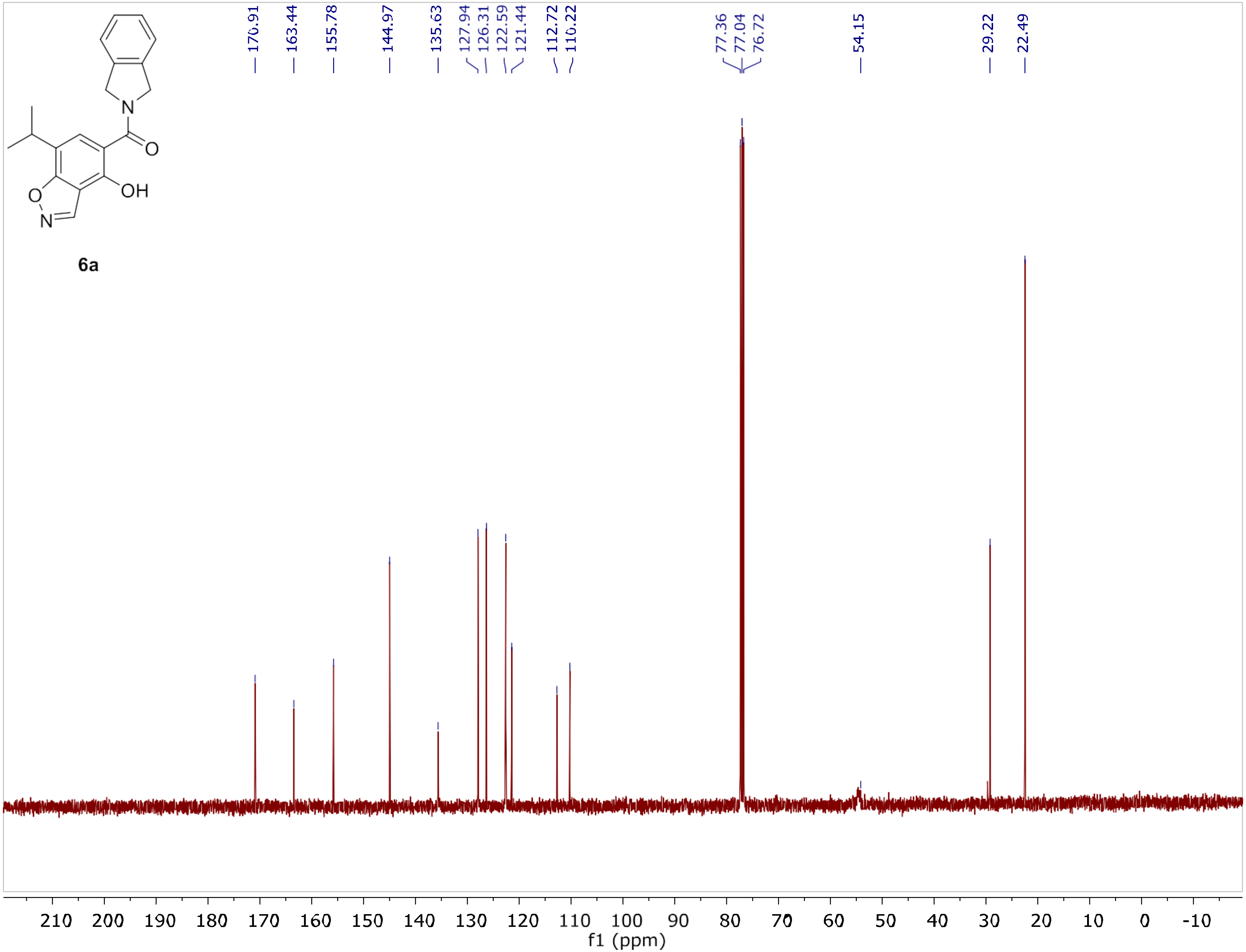

Supplemental Figure 60: H[1] NMR spectra of compound S8.

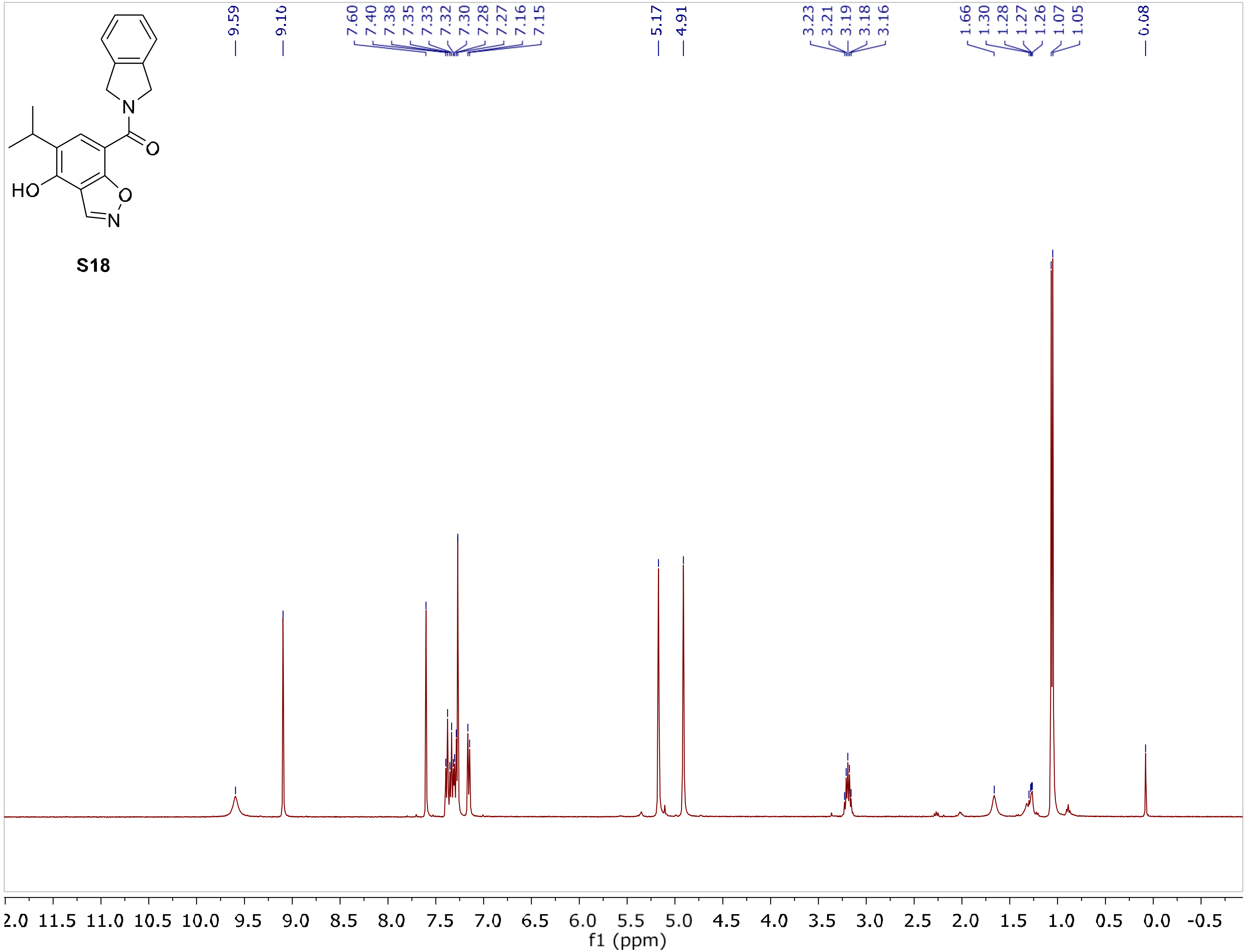

Supplemental Figure 61: C^{13} NMR spectra of compound S18.

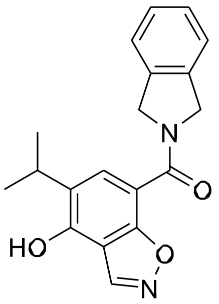

S18

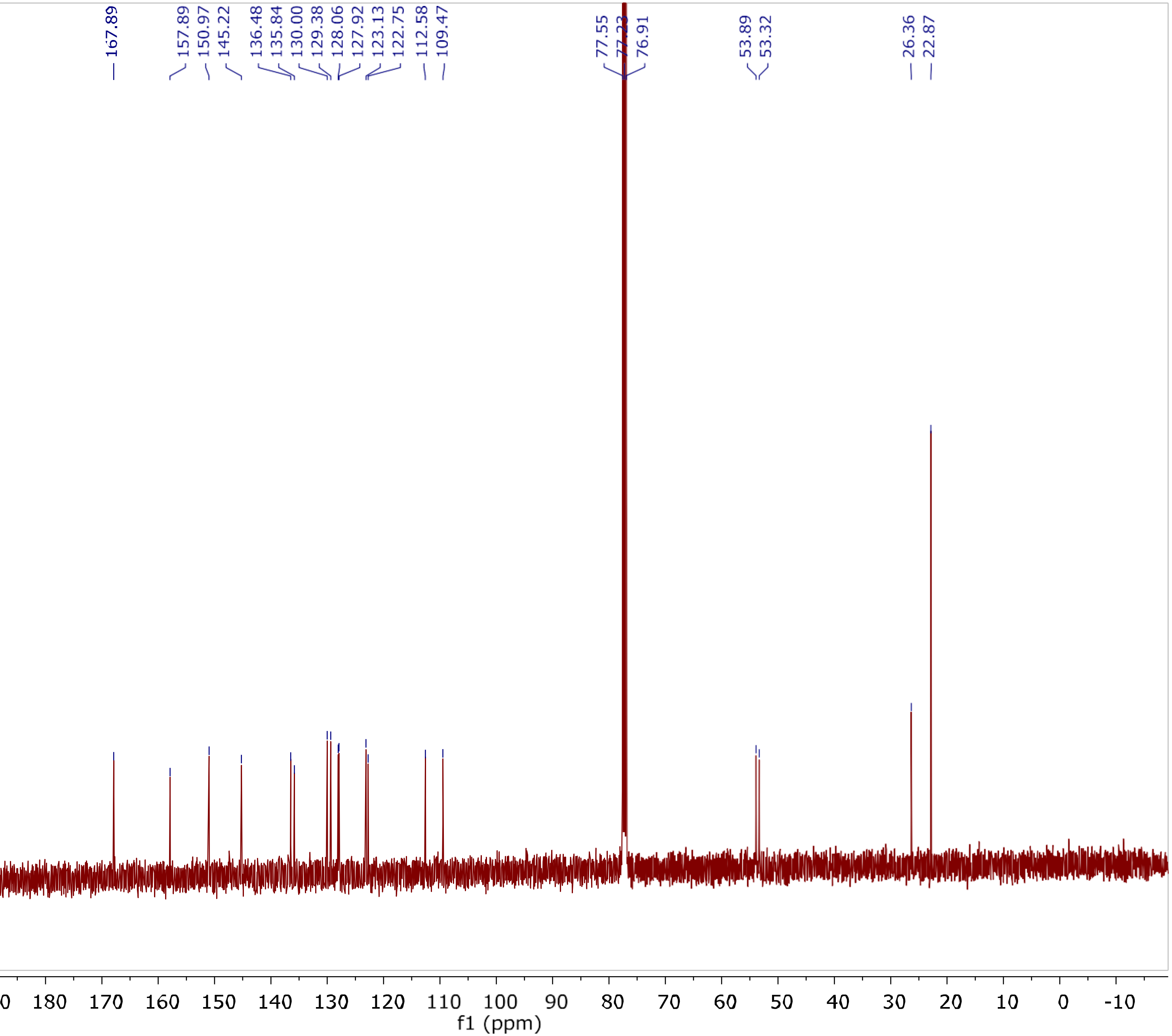

Supplemental Figure 62: H[1] NMR spectra of compound 6b.

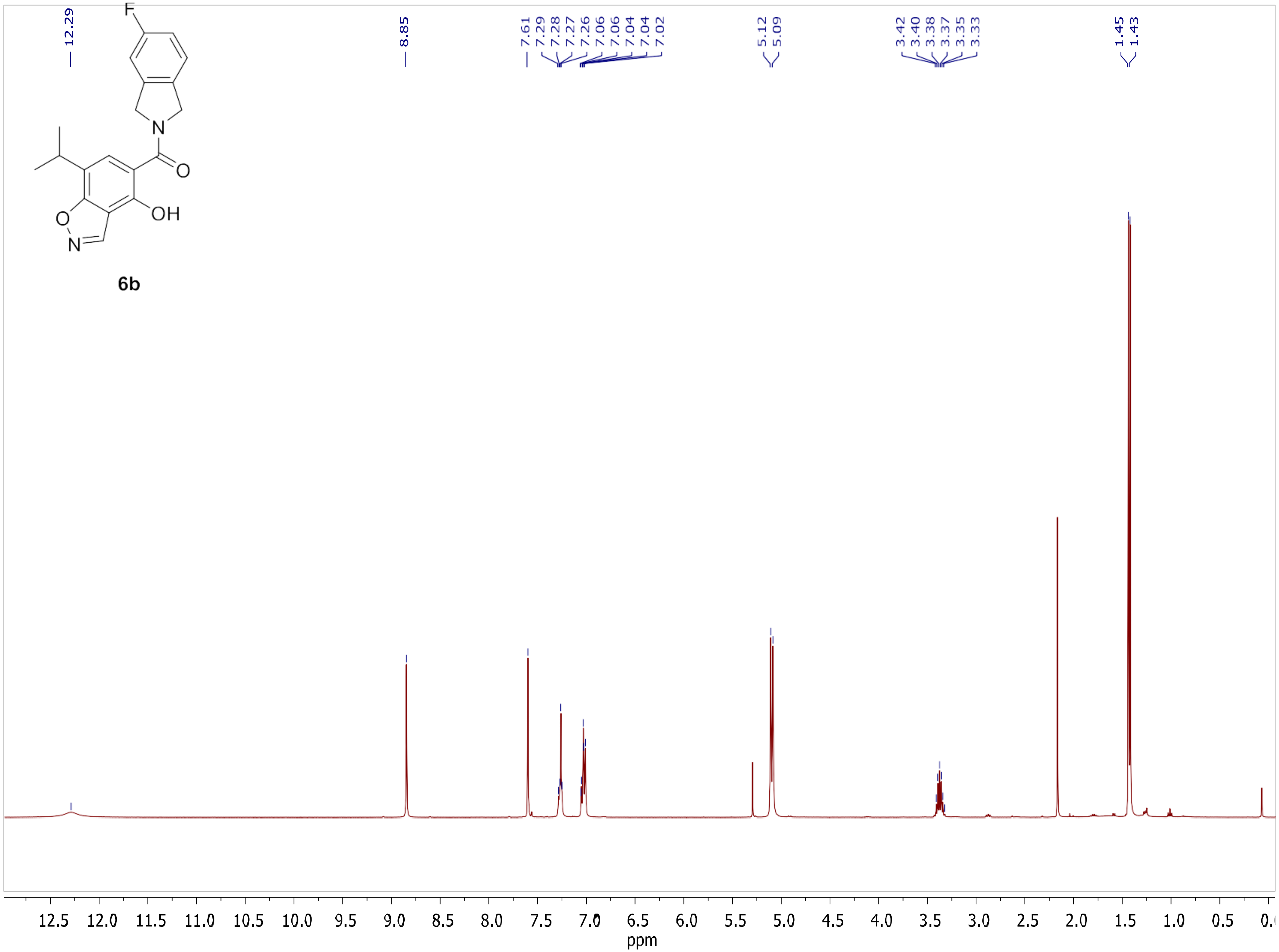

Supplemental Figure 63: C^{13} NMR spectra of compound 6b.

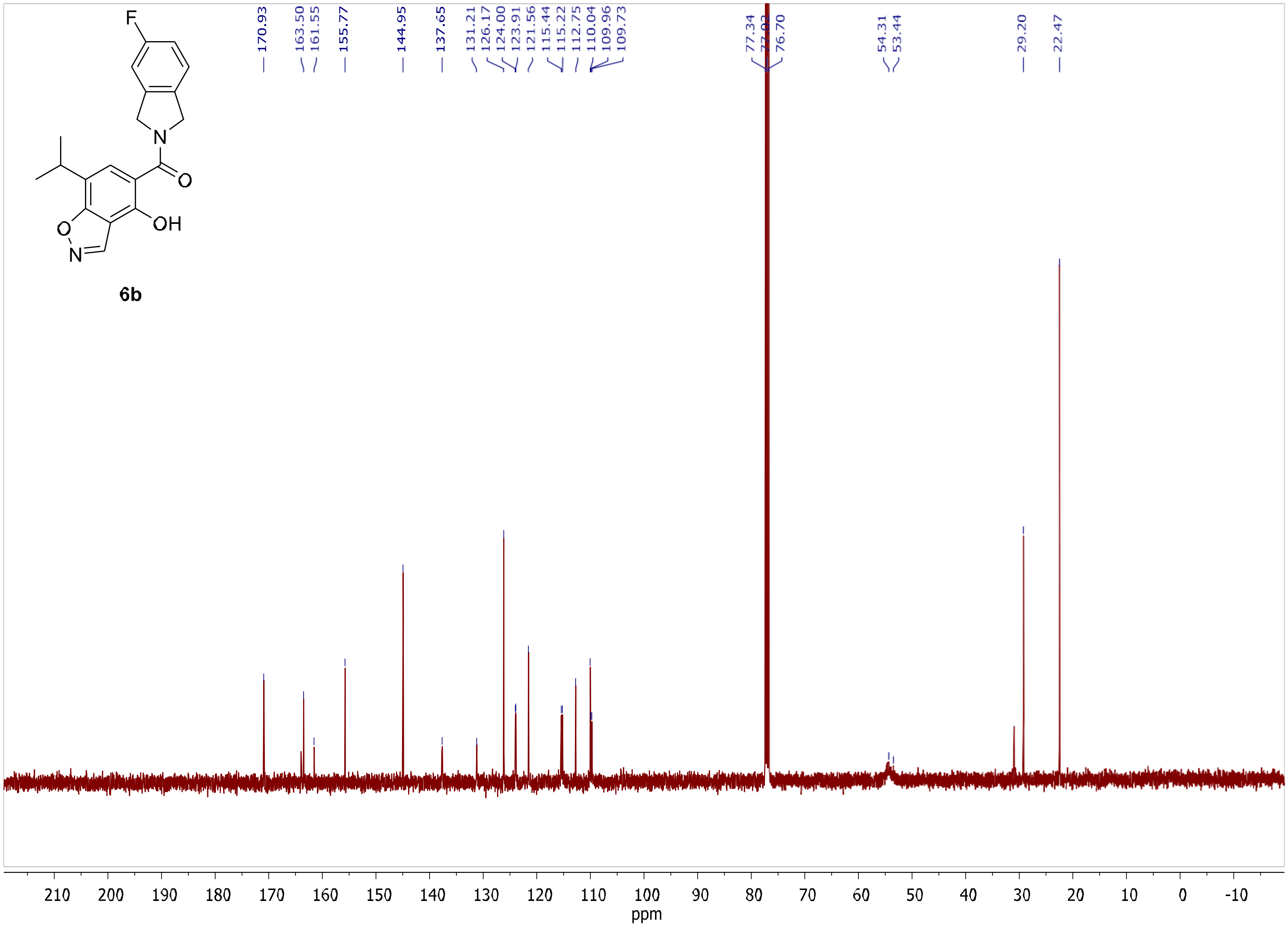

Supplementary References:

1. Data Collection: SMART Software in APEX2 v2014.11-0 Suite. Bruker-AXS, 5465 E. Cheryl Parkway, Madison, WI 53711-5373 USA.

2. Data Reduction: SAINT Software in APEX2 v2014.11-0 Suite. Bruker-AXS, 5465 E. Cheryl Parkway, Madison, WI 53711-5373 USA.

3. Refinement: SHELXTL Software in APEX2 v2014.11-0 Suite. Bruker-AXS, 5465 E. Cheryl Parkway, Madison, WI 53711-5373

4. Woodhead, A. J.; Angove, H.; Carr, M. G.; Chessari, G.; Congreve, M.; Coyle, J. E.; Cosme, J.; Graham, B.; Day, P. J.; Downham, R.; Fazal, L.; Feltell, R.; Figueroa, E.; Frederickson, M.; Lewis, J.; McMenamin, R.; Murray, C. W.; O'Brien, M. A.; Parra, L.; Patel, S.; Phillips, T.; Rees, D. C.; Rich, S.; Smith, D. M.; Trewartha, G.; Vinkovic, M.; Williams, B.; Woolford, A. J. Discovery of (2,4-dihydroxy-5-isopropylphenyl)-[5-(4- methylpiperazin-1-ylmethyl)-1,3-dihydrois oindol-2-yl]methanone (AT13387), a novel inhibitor of the molecular chaperone Hsp90 by fragment based drug design. *J. Med. Chem.* 2010, 53, 5956-5969.

5. Woodhead, A. J.; Angove, H.; Carr, M. G.; Chessari, G.; Congreve, M.; Coyle, J. E.; Cosme, J.; Graham, B.; Day, P. J.; Downham, R.; Fazal, L.; Feltell, R.; Figueroa, E.; Frederickson, M.; Lewis, J.; McMenamin, R.; Murray, C. W.; O'Brien, M. A.; Parra, L.; Patel, S.; Phillips, T.; Rees, D. C.; Rich, S.; Smith, D. M.; Trewartha, G.; Vinkovic, M.; Woolford, A. J. Discovery of (2,4-dihydroxy-5-isopropylphenyl)-[5-(4-methylpiperazin-1-ylmethyl)-1,3-dihydrois oindol-2-yl]methanone (AT13387), a novel inhibitor of the molecular chaperone Hsp90 by fragment based drug design. *J. Med. Chem.* 2010, 53, 5956-5969.

6. Itoh, Y.; Kitaguchi, R.; Ishikawa, M.; Naito, M.; Hashimoto, Y. Design, synthesis and biological evaluation of nuclear receptor-degradation inducers. *Bioorg. Med. Chem.* 2011, 19, 6768-6778

7. Rokade, B. V.; Prabhu, K. R. Chemoselective Schmidt Reaction Mediated by Triflic Acid: Selective Synthesis of Nitriles from Aldehydes. J. Org. Chem. 2012, 77 (12), 5364-70
